# Oncological outcomes of fertility-sparing surgery versus radical surgery in stage - epithelial ovarian cancer: a systematic review and meta-analysis

**DOI:** 10.1186/s12957-024-03440-3

**Published:** 2024-06-25

**Authors:** Ziting Guan, Changlin Zhang, Xinmei Lin, Jingwei Zhang, Tian Li, Jundong Li

**Affiliations:** 1 Department of Gynecology, The Seventh Affiliated Hospital of Sun Yat-Sen University, Shenzhen, China; 2 State Key Laboratory of Oncology in South China, Collaborative Innovation Center for Cancer Medicine, Sun Yat-Sen University Cancer Center, Guangzhou, China; 3 Department of Gynecological Oncology, Sun Yat-Sen University Cancer Center, Guangzhou, China

**Keywords:** Fertility sparing surgery, Radical surgery, Epithelial ovarian cancer, Oncological outcomes

## Abstract

**Background:**

The oncological outcomes of fertility-sparing surgery (FSS) compared to radical surgery (RS) in patients with stage I epithelial ovarian cancer (EOC) remain a subject of debate. We evaluated the risk ratios (RRs) for outcomes in patients with stage I EOC who underwent FSS versus RS.

**Methods:**

We conducted a systematic search of PubMed, Web of Science, and Embase for articles published up to November 29, 2023. Studies that did not involve surgical procedures or included pregnant patients were excluded. We calculated the RRs for disease-free survival, overall survival, and recurrence rate. The quality of the included studies was assessed using the Cochrane Risk of Bias in Nonrandomized Studies of Interventions (ROBINS-I) tool. The meta-analysis was registered on PROSPERO (CRD42024546460).

**Results:**

From the 5,529 potentially relevant articles, we identified 83 articles for initial screening and included 12 articles in the final meta-analysis, encompassing 2,906 patients with epithelial ovarian cancer. There were no significant differences between the two groups in disease-free survival (RR [95% confidence interval {CI}], 0.90 [0.51, 1.58]; *P* = 0.71), overall survival (RR [95% CI], 0.74 [0.53, 1.03]; *P* = 0.07), and recurrence rate (RR [95% CI], 1.10 [0.69, 1.76]; *P* = 0.68). In sensitivity analyses, the significant difference was observed only for overall survival (before exclusion: RR [95% CI], 0.74 [0.53–1.03], *P* = 0.07; after exclusion: RR [95% CI], 0.70 [0.50–0.99]; *P* = 0.04).

**Conclusions:**

This is the first and only individual patient data meta-analysis comparing disease-free survival, overall survival, and recurrence rate of patients with early-stage epithelial ovarian cancer undergoing FSS and RS. FSS was associated with similar disease-free survival and risk of recurrence as RS. We hypothesized that the decreased overall survival in the FSS group could not be attributed to distant metastases from epithelial ovarian cancer.

**Supplementary Information:**

The online version contains supplementary material available at 10.1186/s12957-024-03440-3.

## Background

Ovarian cancer is the fifth leading cause of cancer-related mortality in women of all ages and is a serious threat to women’s health [1]. EOC is the most common histological type of ovarian tumor, originating from the germinal epithelium on the surface of the ovary [2]. Early diagnosis and appropriate therapy for EOC would result in significant improvements in survival [3, 4].

The standard surgical treatment for early-stage EOC is radical surgery (RS), which typically involves bilateral salpingo-oophorectomies, hysterectomies, and omentectomies. Approximately 92% of patients with early-stage EOC do not experience recurrence for at least 5 years after treatment [5]. However, nearly 10% of patients with EOC are aged < 40 years [6]. In nulliparous patients, RS inevitably leads to a loss of reproductive potential, thereby resulting in a decreased quality of life. Thus, fertility-sparing surgery (FSS) was introduced to preserve reproductive function.

FSS is usually defined as the conservation of the uterus and at least part of one ovary, including unilateral cystectomy and unilateral salpingo-oophorectomy. The European guidelines propose that FSS can be safely performed in patients with stage IA and IC1 low-grade ovarian cancer [7]. However, whether the effect of FSS on patients’ survival and disease recurrence is consistent with that of RS remains controversial.

In this review, we extracted the results from previous studies that compared FSS with RS and performed a meta-analysis of the risk ratios (RRs) for disease-free survival (DFS), overall survival (OS), and recurrence rate.

## Methods

This meta-analysis was performed according to the Preferred Reporting Items for Systematic Reviews and Meta-Analyses guidelines [8] (Table S1 and Table S2). No ethical approval or patient consent was required for this meta-analysis because only previously published studies were included. Additionally, it was registered on PROSPERO (CRD42024546460), an international prospective database for reviews.

### Eligibility criteria

Eligible studies including any peer-reviewed research conducted on a population of adult patients with EOC that compared FSS with RS and reported OS and/or DFS as outcome variables. We included articles that provided sufficient data for comparison between FSS and RS. We excluded studies that were not relevant to EOC, did not include both FSS and RS, or involved pregnant patients during treatment. Reviews, case reports, study protocols, commentaries, letters, and abstracts were also excluded.

### Search strategy

A systematic search was conducted in electronic databases, including PubMed, Web of Science, and Embase, for studies published between their inception and November 29, 2023. Reference lists of relevant articles and general reviews were manually reviewed. Language restrictions were not imposed. The search terms combined Medical Subject Headings terms and free-text keywords, including EOC and FSS, and the detailed search terms for each database are presented in Table S3. During each literature search, the titles and abstracts were screened independently by two reviewers (GZT and ZCL). Potentially eligible articles were obtained and subsequently assessed using the full text. Disagreements were resolved by consensus.

### Data extraction

The following data were extracted directly from the studies: author name, country of the author, publication year, study design, sample size, median patient age, International Federation of Gynecology and Obstetrics stage, oncological outcomes, and median follow-up. Oncological outcomes included DFS, OS, and recurrence rate. DFS effectively reflects the clinical benefits of specific diseases, whereas OS also considers patients who died from causes other than EOC. In some studies, DFS has been referred to as cancer-specific survival or tumor-specific survival. Owing to subtle differences in the definition of DFS in various articles, we carefully read the entire text to determine whether death was due to EOC. Publication year, authors, demographic data, sample size, age, race, cancer stage and grade, histological type, tumor size, serum CA125 levels, intraoperative rupture, comorbidities, chemotherapy, study outcomes, and follow-up data were extracted.

### Quality assessment

As the included studies were all non-randomized, their methodological quality was assessed using the Cochrane Risk of Bias in Non-randomized Studies of Interventions (ROBINS-I) tool [9]. The tool covers seven distinct domains in which bias could be introduced, including confounding factors, selection of participants in the study, classification of interventions, deviations from intended interventions, missing data, measurement of outcomes, and selection of the reported result. Before completing the tool properly, we pre-specified potential confounders and co-interventions by considering the pathophysiology of EOC as well as evidence from previous studies. Finally, each domain was judged with “low risk,” “moderate risk,” “serious risk,” or “critical risk” of bias for each study and outcome. If any domain had a serious risk of bias, the study was considered to have an overall serious risk of bias. The detailed risk-of-bias judgements for each study are provided in Table S4 1. All assessments were conducted by two reviewers independently (GZT and ZCL), and discrepancies were resolved through discussion.

### Statistical analysis

Meta-analyses were performed using Review Manager (RevMan) [Computer program], Version 5.4, Cochrane Collaboration, 2020. We calculated the RRs and corresponding 95% confidence intervals (CIs) using a fixed-effects model and the Mantel–Haenszel method. As logRR = 0 corresponded to RR = 1, 95% CIs crossing 1 indicated no effect of the surgical methods on the overall outcome. Statistical significance was set at *P* < 0.05, which was considered significant. The RR and 95% CIs for each outcome are displayed using forest plots. Publication bias was investigated using funnel plots. Heterogeneity was evaluated using the I² statistic, with I² < 25% indicating low heterogeneity. Meta-analyses were also performed for subgroups stratified according to cancer stage. Sensitivity analyses were performed by removing studies item by item and repeating a meta-analysis to evaluate the stability of the results.

## Results

### Characteristics of the included studies

**Fig. 1:**
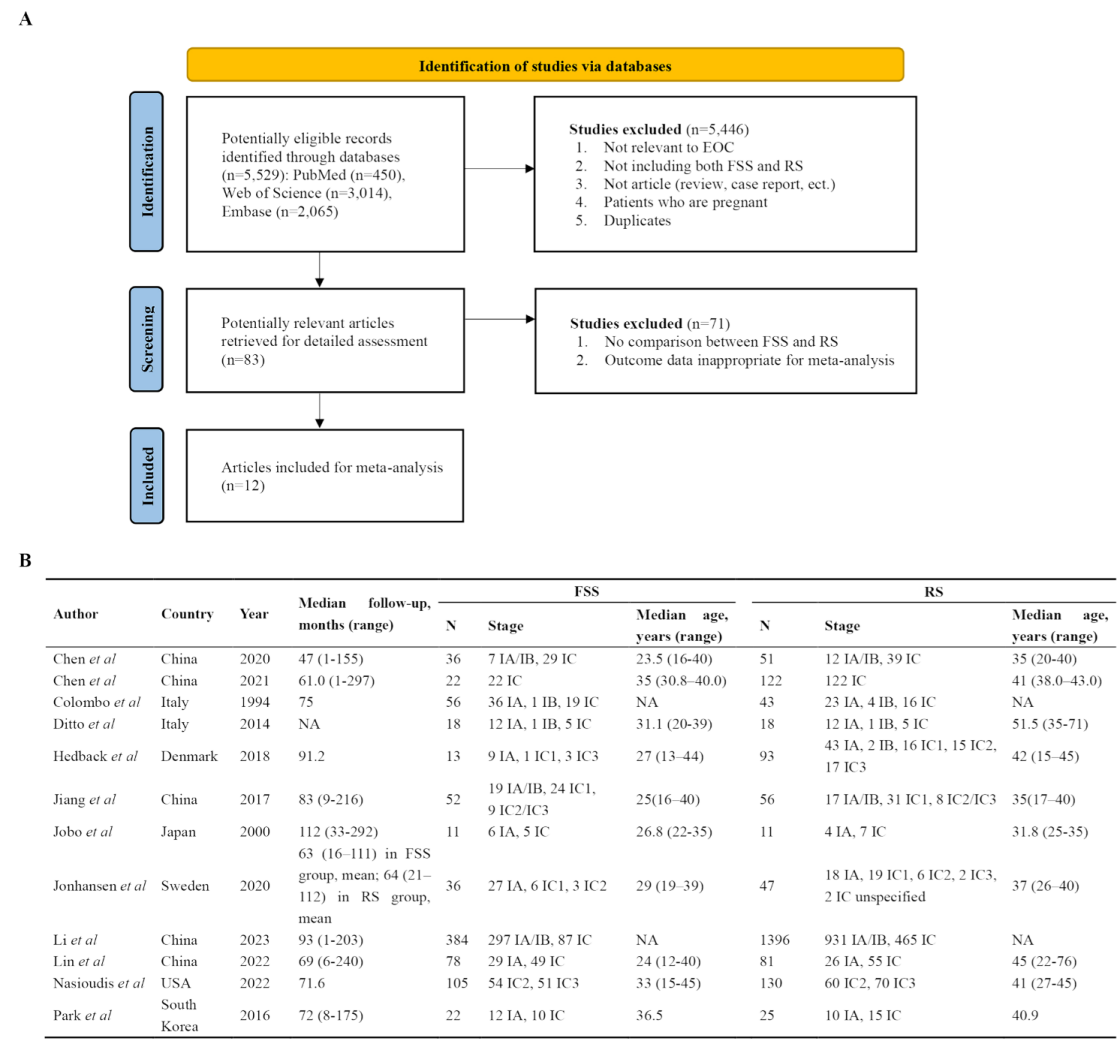
Flowchart and clinical characteristics of included studies. FSS, fertility sparing surgery; RS, radical surgery. (**A**) From an initial 5,529 potentially relevant articles, we identified 83 articles for initial screening and included 12 articles in the final meta-analysis. (**B**) Clinical characteristics of patients with epithelial ovarian cancer undergoing FSS or RS. The age of patients undergoing FSS was significantly lower compared to those undergoing RS

From an initial 5,529 potentially relevant articles, we identified 83 articles for initial screening and included 12 articles in the final meta-analysis [10–21] (Fig. 1A). The included studies comprised patients from China (*n* = 5), Italy (*n* = 2), the United States (*n* = 1), Sweden (*n* = 1), Denmark (*n* = 1), South Korea (*n* = 1), and Japan (*n* = 1). Eleven of the 12 included studies were retrospective, and only one was prospective [17]. All the studies were published in English. The number of patients who underwent FSS ranged from 11 to 384, and the number of patients who underwent RS ranged from 11 to 1,396. All studies included patients with stage I EOC. Detailed clinical characteristics of these studies are shown in Fig. 1B. The age of patients who underwent FSS was significantly lower than that of patients who underwent RS.

### Quality assessment

We evaluated the quality of all included studies and outcomes using ROBINS-I in Fig. S5. The color of each cell indicates the risk of bias for each study and outcome. Overall, the reporting of studies was good: the selection of participants, classification of interventions, management of missing data, measurement of outcomes, and selection of reported results were generally clearly stated. One study was evaluated as having a moderate risk of bias, and seven studies were evaluated as having a serious risk of bias due to incomplete consideration of confounding factors and inappropriate deviations from intended interventions. Further details of the quality assessment are provided in Table S2.

### Disease-free survival

**Fig. 2:**
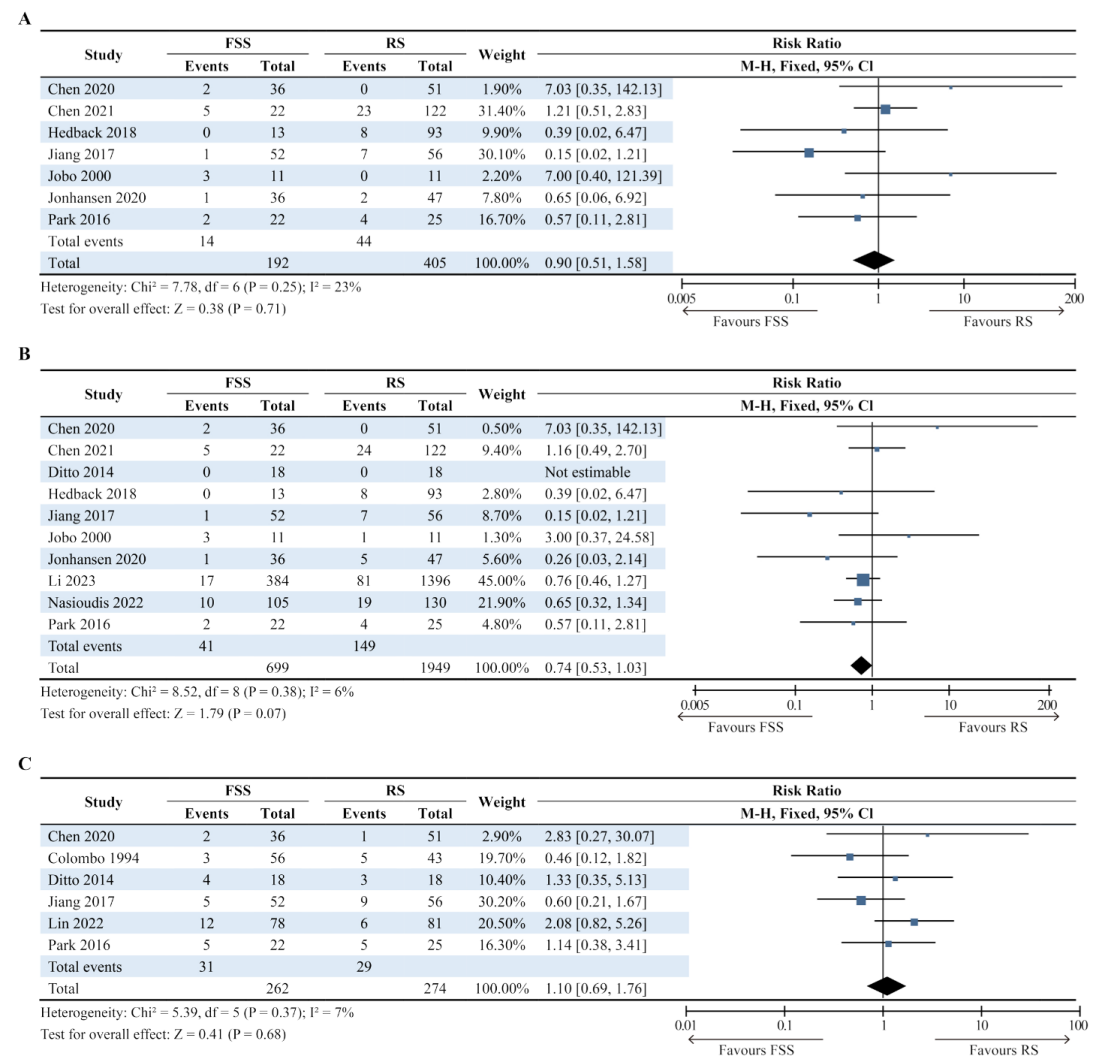
Forest plot of relative risks (RRs) for three outcomes. FSS, fertility sparing surgery; RS, radical surgery. (**A**) Disease-free survival was reported from seven studies. Death occurred in 14 (7.3%) patients in the FSS group and 44 (10.9%) patients in the RS group. There was no significant difference between the two groups (RR [95% confidence interval {CI}], 0.90 [0.51, 1.58]; *P* = 0.71). Heterogeneity was low across studies (I² = 23%, *P* = 0.25). (**B**) In ten studies, 41 (5.9%) participants died in the FSS group and 149 (7.6%) participants died in the RS group. The results showed no significant difference between the two groups (RR [95% CI], 0.74 [0.53, 1.03]; *P* = 0.07) and inter-study heterogeneity was minimal (I² = 6%, *P* = 0.38). (**C**) In six studies, 31 (11.8%) individuals in the FSS group and 29 (10.6%) in the RS group developed recurrence. No significant difference was observed in the recurrence rate between the two groups (RR [95% CI], 1.10 [0.69, 1.76]; *P* = 0.68). There was also low heterogeneity across studies (I² =7%, *P* = 0.37)

DFS was reported from seven studies covering 192 patients in the FSS group and 405 patients in the RS group (Fig. 2A) [10, 11, 14–17, 21]. Death occurred in 14 (7.3%) and 44 (10.9%) patients in the FSS and RS groups, respectively. There was no significant difference between the two groups (RR [95% CI], 0.90 [0.51, 1.58]; *P* = 0.71). Heterogeneity was low across studies (I² = 23%, *P* = 0.25).

### Overall survival

Ten studies involving 699 patients in the FSS group and 1,949 patients in the RS group reported OS (Fig. 2B) [10, 11, 13–18, 20, 21]. In one study [13], the RR could not be calculated because no patients died during follow-up. In total, 41 (5.9%) participants were in the FSS group and 149 (7.6%) were in the RS group. The results showed no significant differences between the two groups (RR [95% CI], 0.74 [0.53, 1.03]; *P* = 0.07), and the inter-study heterogeneity was minimal (I² = 6%, *P* = 0.38).

### Recurrence rate

Recurrence was obtained from six studies, covering 262 patients undergoing FSS and 274 patients undergoing RS (Fig. 2C) [10, 12, 13, 15, 19, 21]. Specifically, 31 (11.8%) individuals in the FSS group and 29 (10.6%) in the RS group experienced recurrence. No significant difference was observed in the recurrence rate between the two groups (RR [95% CI], 1.10 [0.69, 1.76]; *P* = 0.68). There was also low heterogeneity across the studies (I² =7%, *P* = 0.37).

### Subgroup analyses

**Fig. 3:**
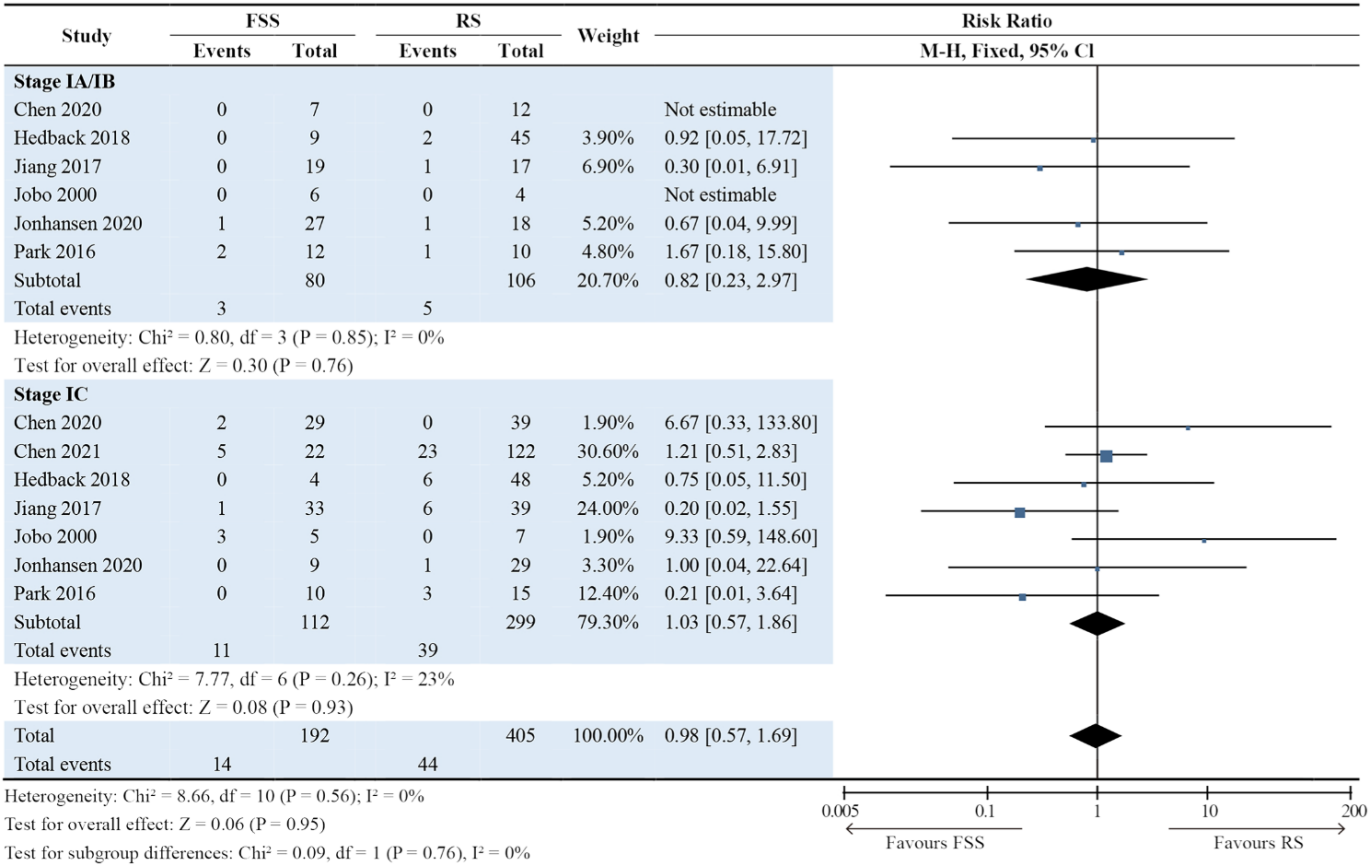
Forest plot presenting subgroup analyses for disease-free survival. The results showed that the International Federation of Gynecology and Obstetrics stage was not associated with surgery procedures in disease-free survival (*P* = 0.95)

**Fig. 4:**
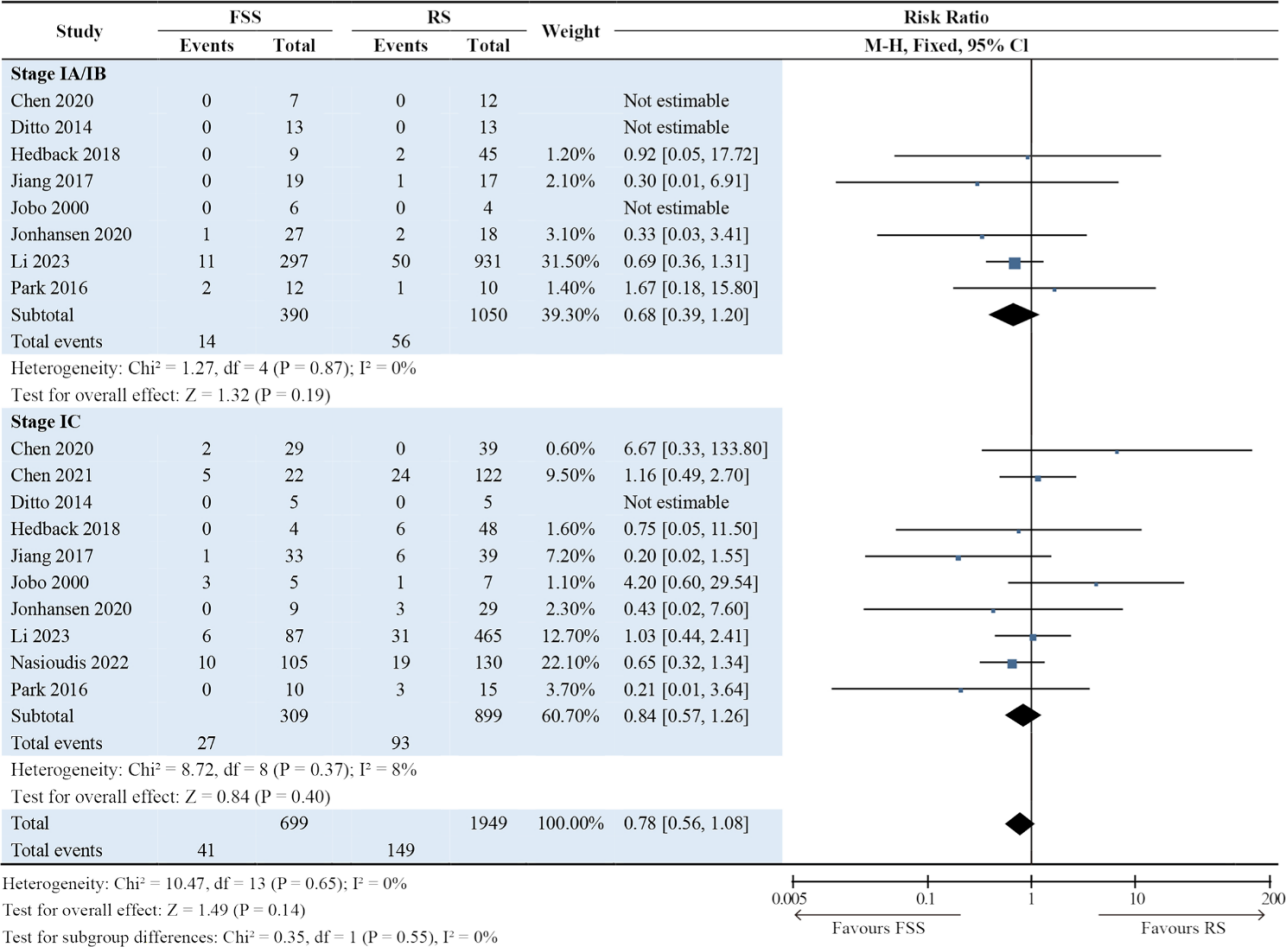
Forest plot presenting subgroup analyses for overall survival. The results showed that the International Federation of Gynecology and Obstetrics stage was not associated with surgery procedures in overall survival (*P* = 0.14)

**Fig. 5:**
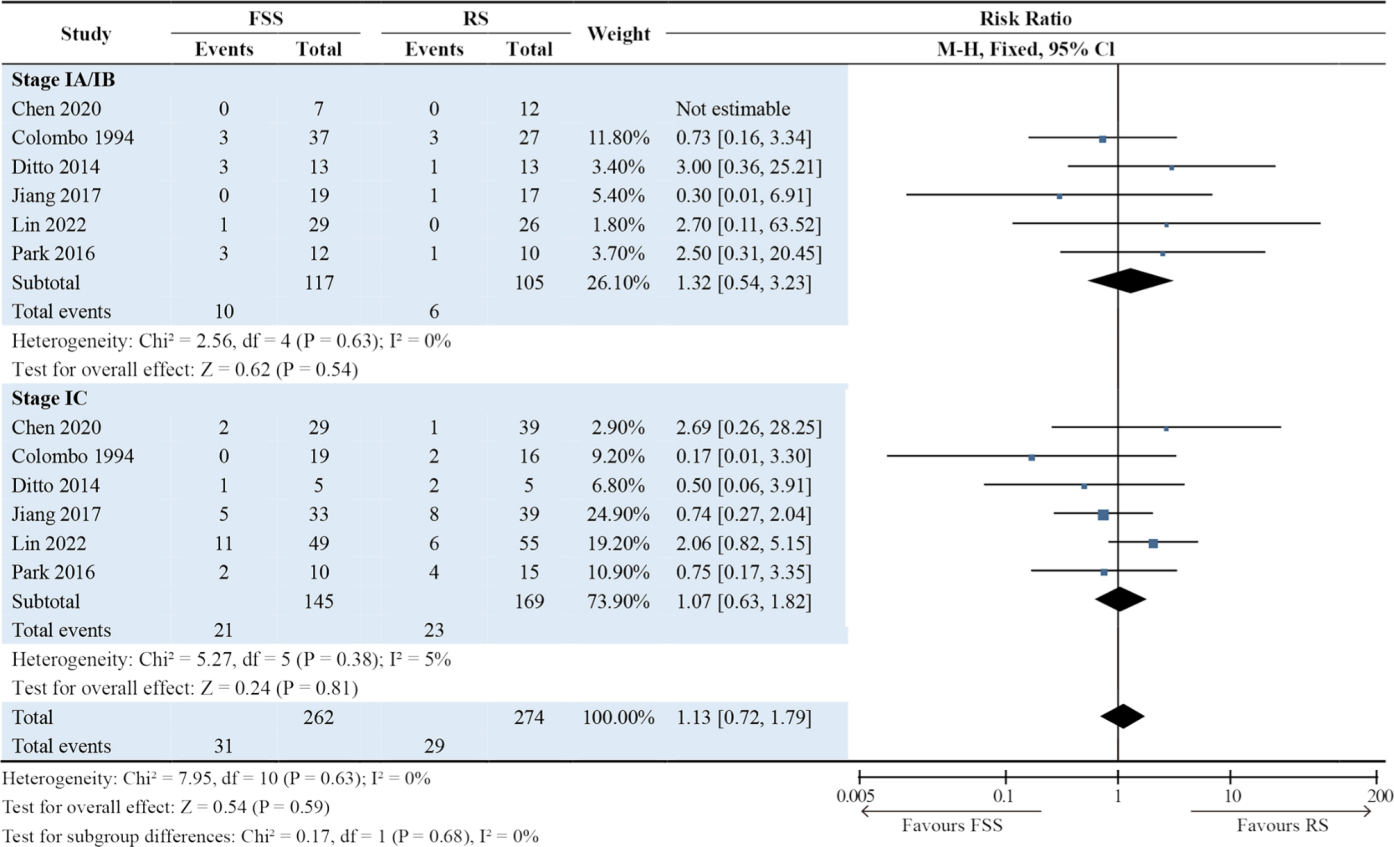
Forest plot presenting subgroup analyses for recurrence rate. The results showed that the International Federation of Gynecology and Obstetrics stage was not associated with surgery procedures in the recurrence rate (*P* = 0.59)

Subgroup analyses were performed by separating the patients in each study into stage IA/IB and IC groups. The results showed that the International Federation of Gynecology and Obstetrics stage was not associated with surgical procedures in terms of DFS (*P* = 0.95; Fig. 3), OS (*P* = 0.14; Fig. 4), and recurrence rate (*P* = 0.59; Fig. 5). For DFS, RRs in the stage IA/IB group and stage IC group were 0.82 (95% CI: 0.23–2.97; *P* = 0.76) and 1.03 (95% CI: 0.57–1.86; *P* = 0.93). For OS, RRs in the stage IA/IB group and stage IC group were 0.68 (95% CI: 0.39–1.20; *P* = 0.19) and 0.84 (95% CI: 0.57–1.26; *P* = 0.40). For recurrence rate, RRs in the stage IA/IB group and stage IC group were 1.32 (95% CI: 0.54–3.23; *P* = 0.54) and 1.07 (95% CI: 0.63–1.82; *P* = 0.81). Subgroup differences were all minimal (DFS: *P* = 0.76; OS: *P* = 0.55; recurrence: *P* = 0.68) (Figs. 3 and 4, and Fig. 5).

### Sensitivity analyses

**Fig. 6:**
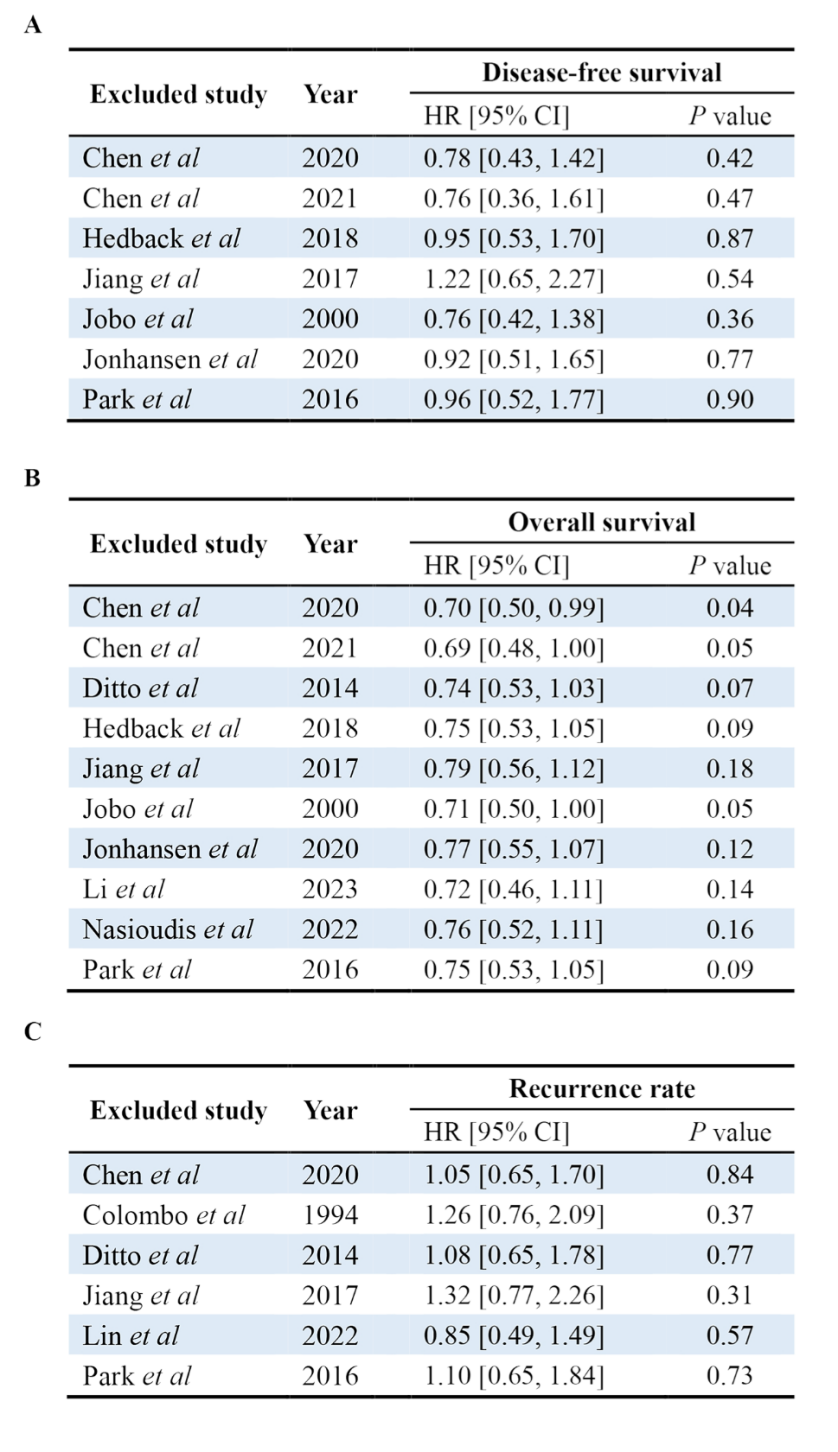
Sensitivity analyses for disease-free survival, overall survival, and recurrence rate. (**A**) The results showed no significant difference for disease-free survival. (**B**) A significant difference in risk ratios was observed when excluding one study for overall survival. Removing this study resulted in a statistically significant difference between the two surgical procedures (before exclusion: relative risk [RR] [95% confidence interval {CI}], 0.74 [0.53–1.03], *P* = 0.07; after exclusion: RR [95% CI], 0.70 [0.50–0.99]; *P* = 0.04). (**C**) The results showed no significant difference for the recurrence rate

Sensitivity analyses were performed by sequentially excluding studies and repeating a meta-analysis to estimate the effect of each study on the outcomes. Figure 6A, 6B, and 6C list the DFS, OS, and recurrence rate recalculated after removing each study, respectively. A significant difference in RR was observed only when one study was excluded for OS [10]. Because the heterogeneity of the original meta-analysis was low (I² = 6%, *P* = 0.38; Fig. 3B), the reduction in heterogeneity (I² = 0%, *P* = 0.49) was not statistically significant. However, removing this study resulted in a statistically significant difference between the two surgical procedures (before exclusion: RR [95% CI], 0.74 [0.53–1.03], *P* = 0.07; after exclusion: RR [95% CI], 0.70 [0.50–0.99]; *P* = 0.04).

### Publication bias

The results showed no evidence of publication bias in the meta-analyses or subgroup analyses for DFS, OS, and recurrence rate. The funnel plots are symmetrical, as shown in Fig. S6.

## Discussion

Due to the delayed childbearing age, young women initially diagnosed with EOC are often nulliparous and desire to preserve their reproductive functions. Fertility-sparing surgery of epithelial ovarian cancer was based on the conservation of the uterus and at least part of one ovary and complete surgical staging. It was initially proposed for young women with early-stage invasive tumors and a lower risk of recurrence. Current guidelines and related reviews suggested that early-stage EOC could be treated with FSS [22, 23]. However, compared with radical surgery, whether it could lead to worse oncological outcomes remains inconclusive.

Our study suggested that patients with early-stage epithelial ovarian cancer who had the need and willingness to preserve fertility could undergo conservative surgery with confidence regardless of the substages. There was no significant difference in DFS, OS and recurrence rate between FSS and RS. Substages in early-stage EOC were also not related with oncological results. Both physical health and reproductive ability are of great importance for young women. Our conclusion has positive implications for promoting family happiness and social development.

We have extensively collected relevant literature and conducted comprehensive analyses of included studies. Sensitivity analyses indicated that the conclusion was robust and reliable in analyses of DFS and recurrence rate. However, it came to an opposite conclusion in analyses of OS when removing one study and repeating the meta-analysis [10]. We suggest that this was because the significance with the upper limit of the CI crossed only one value (RR [95% CI], 0.74 [0.53–1.03]; *P* = 0.07) (Fig. 2B). Although the weight in this study was only 0.5%, its significance changed after its removal. After exclusion, the upper limit of CI was very close but did not cross one value (RR [95% CI], 0.70 [0.50–0.99]; *P* = 0.04) (Fig. 6B), indicating patients who underwent FSS had worse OS than those who underwent RS.

Since the results for DFS and recurrence rate showed no significant differences, we assumed that for patients undergoing FSS, the decrease in overall survival rate could not be attributed to the metastasis of ovarian cancer. A prospective study [17] providing detailed information on recurrence and death cause indicated that some individuals died of lung cancer, jejunal cancer, or unknown causes without recurrence. Since analyses of overall survival were not as robust as analyses of DFS and recurrence, additional clinical trials are needed for further validation. Moreover, the subgroup effects divided by stage IA/IB and stage IC were not significant for any of the three outcomes. No publication bias was evident in either the meta-analyses or the subgroup analyses.

Regarding the clinical characteristics of all the patients, individuals in the FSS group were younger than those in the RS group. However, no consensus has been reached regarding the influence of age on survival and recurrence. Some studies have suggested that younger age is associated with a better prognosis [6, 15, 24], while others found that age, independently related to prognosis and surgical procedure, had no significant effect on outcomes [24]. In most of the included studies, age was not adjusted for in the FSS and RS groups. Considering that young women are more likely to preserve their fertility, we treated age as a confounding factor that acted as an indicator for evaluating the quality of a study. We believe that this could have guiding significance in clinical work.

Some hot topics of the oncological outcomes mainly include the following points. Firstly, if the tumor grading could influence the risk of recurrence remains unclear. Patients with low-grade tumor tended to have more favorable outcomes than those with high-grade tumor [15, 18]. Secondly, oncological outcomes in substages of stage IC are still controversial [25]. In the new FIGO staging system, stage IC ovarian cancer is further subdivided in stage IC1, IC2 and IC3. Patients who diagnosed with stage IC2 or IC3 EOC have a higher risk of recurrence compared with those with stage IC1. Thirdly, “high risk” histologic subtypes, particularly clear-cell ovarian tumors, could negatively influence survival and recurrence [26].

Currently, minimally invasive surgical approaches have also received widespread attention. In the past decade, minimally invasive surgery has completely changed the surgical management of gynecological malignancies, including early ovarian cancer [27, 28]. Previous studies showed that there was no difference between different surgical approaches in long-term oncological outcomes with regard to tumor grading, histology and final FIGO stage [29, 30]. It is a valuable therapeutic option with advantages of less postoperative pain, less complications and faster recovery. Further clinical researches are needed to confirm its safety and reliability.

This study mainly explores whether patients with early EOC can undergo conservation surgery, which is essentially about the issue of individualized treatment. It is of great significance to make a careful evaluation and provide a personalized treatment in particular in case of elderly women. For instance, some patients with distant metastasis and high tumor load can still benefit from high complex multi-visceral surgery and the incidence associated with surgery is acceptable [31]. Besides, patients with advanced ovarian cancer have a higher risk of lymph node metastases. But there still exists controversy over the therapeutic role of lymphadenectomy [32–35]. A recent review comprehensively discussed the survival benefits in patients undergoing lymphadenectomy [36], suggesting that in some cases retroperitoneal staging was not related to better prognosis but increased postoperative complications.

Our study had some limitations. First, it was based on evidence from non-randomized controlled trials (non-RCTs) and provided a lower level of evidence than RCTs. Only one study was prospective, and the other 11 were retrospective. The development of surgical procedures for patients with early-stage EOC should fully consider the patients’ desire to preserve birth function. RCTs are challenging to perform in this context and may raise ethical concerns. Additionally, the data collection on confounding factors and intended interventions were not sufficiently detailed in most included studies. Some studies did not differentiate between the use and nonuse of chemotherapy, which may have influenced our conclusions.

To our knowledge, this is the first and only individual patient data meta-analysis to use the ROBINS-I tool to comprehensively evaluate the quality of observational studies, summarizing the DFS, OS, and recurrence rate of patients with early-stage EOC who underwent FSS and RS. Strengths include a rigorous study protocol with prespecified analyses, standardized outcome definitions across all included trials, selection of confounding factors and intended interventions, and prespecified subgroup analyses. We suggest that all included studies clearly state their selection of participants, classification of interventions, management of missing data, measurement of outcomes, and selection of reported results.

## Conclusions

This is the first and only individual patient data meta-analysis comparing disease-free survival, overall survival, and recurrence rate of patients with early-stage epithelial ovarian cancer undergoing FSS and radical surgery. FSS was associated with a similar disease-free survival and risk of recurrence as radical surgery. Decreased overall survival in the FSS group could not be attributed to distant metastases from epithelial ovarian cancer. We believe that this could have guiding significance in clinical work.

### Electronic supplementary material

Below is the link to the electronic supplementary material.

Supplementary Material 1

Supplementary Material 2

Supplementary Material 3

Supplementary Material 4

Supplementary Material 5

## Data Availability

Data from the included studies were obtained from original articles for the specific purpose of conducting this systematic review and meta-analysis. Any request from other researchers for these data should be directed to the authors of the corresponding article.

## Declarations

### Generative AI and AI-assisted technologies in the writing process

We did not use artificial intelligence or AI-assisted technologies in the writing process.

## Abbreviations

FSS: Fertility-sparing surgery
RS: Radical surgery
CI: Confidence interval
RR: Risk ratio
EOC: Epithelial ovarian cancer
DFS: Disease-free survival
OS: Overall survival
ROBINS-I: Risk of Bias in Non-randomized Studies of Interventions
