# Supplementary material for: Oncological outcomes of fertility-sparing surgery versus radical surgery in stage - epithelial ovarian cancer: a systematic review and meta-analysis
Source: World J Surg Oncol. 2024 Jun 25;22:170. doi: 10.1186/s12957-024-03440-3 (PMC11201297; doi:10.1186/s12957-024-03440-3)
Supplement: Supplementary file 1 — Supplementary Material 1 [file 12957_2024_3440_MOESM1_ESM.pdf]

ROBINS-I tool (Stage I): At protocol stage

Participants	Patients with epithelial ovarian carcinoma
Experimental intervention	FSS
Comparator	RS
Outcomes	Recurrence rate, DFS, OS, CSS, TSS
Confounding domains	Age, Race, Stage, Grade, Histology, Tumor size, Elevated serum CA125, Intraoperative rupture, Co-morbidities
Co-interventions	Chemotherapy

**Unique ID**	Chen 2020	**Ref or Label**	Current Medical Science 2020; 40(4):745-752	**Design**	Individually randomized
**Participants**	Patients with epithelial ovarian carcinoma	**Experimental**	FSS	**Comparator**	RS
**Aim**	assignment to intervention (the 'intention-to-treat' effect)	**Outcome**	DFS	**Result**	Hazard ratio, 2.07

**Confounding domains listed in the review protocol**	**Measured variable(s)**	**Is there evidence that controlling for this variable was unnecessary?**	**Is the confounding domain measured validly and reliably by this variable?**	
Age	NA	No	Yes	
Race	NA	No	Yes	
Stage	Pathology report	No	Yes	
Grade	Pathology report	No	Yes	
Histology	Pathology report	No	Yes	
Tomor size	Clinical data	No	Yes	
Elevated serum CA125	Clinical data	No	Yes	
Intraoperative rupture	Clinical data	No	Yes	
Co-morbidities	Clinical data	No	Yes	

**Additional confounding domains**	**Measured variable(s)**	**Is there evidence that controlling for this variable was unnecessary?**	**Is the confounding domain measured validly and reliably by this variable?**	**OPTIONAL: Is failure to adjust for this variable (alone) expected to favour the experimental intervention or the comparator?**
NA	NA	NA	NA	NA

**Co-interventions listed in the review protocol**	**Is there evidence that controlling for this co-intervention was unnecessary (e.g. because it was not administered)?**	**Is presence of this co-intervention likely to favour outcomes in the experimental intervention or the comparator**
Chemotherapy	No	No information

**Additional co-interventions**	**Is there evidence that controlling for this co-intervention was unnecessary (e.g. because it was not administered)?**	**Is presence of this co-intervention likely to favour outcomes in the experimental intervention or the comparator**
NA	NA	NA

**Domain**	**Signalling questions**	**Response options**	**Description**
**Bias due to confounding**	1.1 Is there potential for confounding of the effect of intervention in this study?	Y	Differentiation grade and histological type were significantly different between FSS and radical surgery groups.
	1.2 If Y/PY to 1.1: Was the analysis based on splitting participants' follow up time according to intervention received?	N	
	1.3 If Y/PY to 1.2: Were intervention discontinuations or switches likely to be related to factors that are prognostic for the outcome?	NA	
	**Questions relating to baseline confounding only**		
	1.4 If N/PN to 1.2 or 1.3: Did the authors use an appropriate analysis method that controlled for all the important confounding domains?	Y	P value: Age (years) <0.001; FIGO stage 0.650; Differentiation grade 0.002; Histological type <0.001. However, as depicted in fig. 1, the DFS and OS was analyzed by the Kaplan-Meier curve. When stratified by FIGO stage, histological subtype, and differentiation grade, there were also no significant differences
	1.5 If Y/PY to 1.4: Were confounding domains that were controlled for measured validly and reliably by the variables available in this study?	Y	
	1.6. If N/PN to 1.2 or 1.3: Did the authors control for any post-intervention variables that could have been affected by the intervention?	Y	
	**Questions relating to baseline and time-varying confounding**		
	1.7. If Y/PY to 1.3: Did the authors use an appropriate analysis method that controlled for all the important confounding domains and for time-varying confounding?	NA	
	1.8. If Y/PY to 1.7: Were confounding domains that were controlled for measured validly and reliably by the variables available in this study?	NA	
	**Risk of bias judgement**	**Low**	
**Bias in selection of participants into the study**	2.1 Was selection of participants into the study (or into the analysis) based on participant characteristics observed after the start of intervention?	N	
	2.2 If Y/PY to 2.1: Were the post-intervention variables that influenced selection likely to be associated with intervention?	NA	
	2.3 If Y/PY to 2.2: Were the post-intervention variables that influenced selection likely to be influenced by the outcome or a cause of the outcome?	NA	
	2.4 If N/PN to 2.1: Do start of follow-up and start of intervention coincide for most participants?	PY	
	2.5. If Y/PY to 2.2 and 2.3, or N/PN to 2.4: Were adjustment techniques used that are likely to correct for the presence of selection biases?	NA	
	**Risk of bias judgement**	**Low**	
**Bias in classification of interventions**	3.1 Were intervention groups clearly defined?	Y	FSS was defined as surgery sparing the uterus and contralateral ovarian tissue to allow natural conception in the future. Women who had undergone hysterectomy and/or removal of the contralateral ovary were included in the radical surgery group.
	3.2 Was the information used to define intervention groups recorded at the start of the intervention?	Y	
	3.3 Could classification of intervention status have been affected by knowledge of the outcome or risk of the outcome?	N	
	**Risk of bias judgement**	**Low**	
**Bias due to deviations from intended interventions**	**If your aim for this study is to assess the effect of assignment to intervention, answer questions 4.1 and 4.2**		
	4.1. Were there deviations from the intended intervention beyond what would be expected in usual practice?	Y	Other clinicopathological factors, surgical procedures, and postoperative adjuvant therapy were similar between these groups.
	4.2. If Y/PY to 4.1: Were these deviations from intended intervention unbalanced between groups and likely to have affected the outcome?	N	
	**If your aim for this study is to assess the effect of starting and adhering to intervention, answer questions 4.3 to 4.6**		
	4.3. Were important co-interventions balanced across intervention groups?	NA	
	4.4. Was the intervention implemented successfully for most participants?	NA	
	4.5. Did study participants adhere to the assigned intervention regimen?	NA	
	4.6. If N/PN to 4.3, 4.4 or 4.5: Was an appropriate analysis used to estimate the effect of starting and adhering to the intervention?	NA	
	**Risk of bias judgement**	**Low**	
**Bias due to missing data**	5.1 Were outcome data available for all, or nearly all, participants?	Y	Missing data were completed by telephone interview or by mail sent multiple times during follow-up.
	5.2 Were participants excluded due to missing data on intervention status?	PN	
	5.3 Were participants excluded due to missing data on other variables needed for the analysis?	PN	
	5.4 If PN/N to 5.1, or Y/PY to 5.2 or 5.3: Are the proportion of participants and reasons for missing data similar across interventions?	NA	

	5.5 If PN/N to 5.1, or Y/PY to 5.2 or 5.3: Is there evidence that results were robust to the presence of missing data?	NA	
	**Risk of bias judgement**	**Low**	
**Bias in measurement of the outcome**	6.1 Could the outcome measure have been influenced by knowledge of the intervention received?	N	DFS was measured from the start of treatment until date of detection of recurrence.
	6.2 Were outcome assessors aware of the intervention received by study participants?	PY	
	6.3 Were the methods of outcome assessment comparable across intervention groups?	Y	
	6.4 Were any systematic errors in measurement of the outcome related to intervention received?	PN	
	**Risk of bias judgement**	**Low**	
**Bias in selection of the reported result**	Is the reported effect estimate likely to be selected, on the basis of the results, from…		
	7.1. ... multiple outcome measurements within the outcome domain?	PN	
	7.2 ... multiple analyses of the intervention-outcome relationship?	PN	
	7.3 ... different subgroups?	PN	
	**Risk of bias judgement**	**Low**	
**Overall bias**	**Risk of bias judgement**	**Low**	

**Unique ID**	Chen 2020	**Ref or Label**	Current Medical Science 2020; 40(4):745-752	**Design**	Individually randomized
**Participants**	Patients with epithelial ovarian carcinoma	**Experimental**	FSS	**Comparator**	RS
**Aim**	assignment to intervention (the 'intention-to-treat' effect)	**Outcome**	Recurrence rate	**Result**	5.56% in FSS and 1.96% in RS.

**Confounding domains listed in the review protocol**	**Measured variable(s)**	**Is there evidence that controlling for this variable was unnecessary?**	**Is the confounding domain measured validly and reliably by this variable?**	
Age	NA	No	Yes	
Race	NA	No	Yes	
Stage	Pathology report	No	Yes	
Grade	Pathology report	No	Yes	
Histology	Pathology report	No	Yes	
Tomor size	Clinical data	No	Yes	
Elevated serum CA125	Clinical data	No	Yes	
Intraoperative rupture	Clinical data	No	Yes	
Co-morbidities	Clinical data	No	Yes	
**Additional confounding domains**	**Measured variable(s)**	**Is there evidence that controlling for this variable was unnecessary?**	**Is the confounding domain measured validly and reliably by this variable?**	**OPTIONAL: Is failure to adjust for this variable (alone) expected to favour the experimental intervention or the comparator?**
NA	NA	NA	NA	NA

**Co-interventions listed in the review protocol**	**Is there evidence that controlling for this co-intervention was unnecessary (e.g. because it was not administered)?**	**Is presence of this co-intervention likely to favour outcomes in the experimental intervention or the comparator**
Chemotherapy	No	No information
**Additional co-interventions**	**Is there evidence that controlling for this co-intervention was unnecessary (e.g. because it was not administered)?**	**Is presence of this co-intervention likely to favour outcomes in the experimental intervention or the comparator**
NA	NA	NA

**Domain**	**Signalling questions**	**Response options**	**Description**
**Bias due to confounding**	1.1 Is there potential for confounding of the effect of intervention in this study?	Y	Differentiation grade and histological type were significantly different between FSS and radical surgery groups.
	1.2 If Y/PY to 1.1: Was the analysis based on splitting participants' follow up time according to intervention received?	N	
	1.3 If Y/PY to 1.2: Were intervention discontinuations or switches likely to be related to factors that are prognostic for the outcome?	NA	
	**Questions relating to baseline confounding only**		
	1.4 If N/PN to 1.2 or 1.3: Did the authors use an appropriate analysis method that controlled for all the important confounding domains?	Y	P value: Age (years) <0.001; FIGO stage 0.650; Differentiation grade 0.002; Histological type <0.001. However, as depicted in fig. 1, the DFS and OS was analyzed by the Kaplan-Meier curve. When stratified by FIGO stage, histological subtype, and differentiation grade, there were also no significant differences
	1.5 If Y/PY to 1.4: Were confounding domains that were controlled for measured validly and reliably by the variables available in this study?	Y	
	1.6. If N/PN to 1.2 or 1.3: Did the authors control for any post-intervention variables that could have been affected by the intervention?	Y	
	**Questions relating to baseline and time-varying confounding**		
	1.7. If Y/PY to 1.3: Did the authors use an appropriate analysis method that controlled for all the important confounding domains and for time-varying confounding?	NA	
	1.8. If Y/PY to 1.7: Were confounding domains that were controlled for measured validly and reliably by the variables available in this study?	NA	
	**Risk of bias judgement**	**Low**	
**Bias in selection of participants into the study**	2.1 Was selection of participants into the study (or into the analysis) based on participant characteristics observed after the start of intervention?	N	
	2.2 If Y/PY to 2.1: Were the post-intervention variables that influenced selection likely to be associated with intervention?	NA	
	2.3 If Y/PY to 2.2: Were the post-intervention variables that influenced selection likely to be influenced by the outcome or a cause of the outcome?	NA	
	2.4 If N/PN to 2.1: Do start of follow-up and start of intervention coincide for most participants?	PY	
	2.5. If Y/PY to 2.2 and 2.3, or N/PN to 2.4: Were adjustment techniques used that are likely to correct for the presence of selection biases?	NA	
	**Risk of bias judgement**	**Low**	
**Bias in classification of interventions**	3.1 Were intervention groups clearly defined?	Y	FSS was defined as surgery sparing the uterus and contralateral ovarian tissue to allow natural conception in the future. Women who had undergone hysterectomy and/or removal of the contralateral ovary were included in the radical surgery group.
	3.2 Was the information used to define intervention groups recorded at the start of the intervention?	Y	
	3.3 Could classification of intervention status have been affected by knowledge of the outcome or risk of the outcome?	N	

	**Risk of bias judgement**	**Low**	
**Bias due to deviations from intended interventions**	**If your aim for this study is to assess the effect of assignment to intervention, answer questions 4.1 and 4.2**		
	4.1. Were there deviations from the intended intervention beyond what would be expected in usual practice?	Y	Other clinicopathological factors, surgical procedures, and postoperative adjuvant therapy were similar between these groups.
	4.2. If Y/PY to 4.1: Were these deviations from intended intervention unbalanced between groups and likely to have affected the outcome?	N	
	**If your aim for this study is to assess the effect of starting and adhering to intervention, answer questions 4.3 to 4.6**		
	4.3. Were important co-interventions balanced across intervention groups?	NA	
	4.4. Was the intervention implemented successfully for most participants?	NA	
	4.5. Did study participants adhere to the assigned intervention regimen?	NA	
	4.6. If N/PN to 4.3, 4.4 or 4.5: Was an appropriate analysis used to estimate the effect of starting and adhering to the intervention?	NA	
	**Risk of bias judgement**	**Low**	
**Bias due to missing data**	5.1 Were outcome data available for all, or nearly all, participants?	Y	Missing data were completed by telephone interview or by mail sent multiple times during follow-up.
	5.2 Were participants excluded due to missing data on intervention status?	PN	
	5.3 Were participants excluded due to missing data on other variables needed for the analysis?	PN	
	5.4 If PN/N to 5.1, or Y/PY to 5.2 or 5.3: Are the proportion of participants and reasons for missing data similar across interventions?	NA	
	5.5 If PN/N to 5.1, or Y/PY to 5.2 or 5.3: Is there evidence that results were robust to the presence of missing data?	NA	
	**Risk of bias judgement**	**Low**	
**Bias in measurement of the outcome**	6.1 Could the outcome measure have been influenced by knowledge of the intervention received?	N	
	6.2 Were outcome assessors aware of the intervention received by study participants?	PY	
	6.3 Were the methods of outcome assessment comparable across intervention groups?	Y	
	6.4 Were any systematic errors in measurement of the outcome related to intervention received?	PN	
	**Risk of bias judgement**	**Low**	
**Bias in selection of the reported result**	Is the reported effect estimate likely to be selected, on the basis of the results, from…		
	7.1. ... multiple outcome measurements within the outcome domain?	PN	
	7.2 ... multiple analyses of the intervention-outcome relationship?	PN	
	7.3 ... different subgroups?	PN	
	**Risk of bias judgement**	**Low**	
**Overall bias**	**Risk of bias judgement**	**Low**	

**Unique ID**	Chen 2021	**Ref or Label**	Arch Gynecol Obstet. 2021;304(2):521-529.	**Design**	Individually randomized
**Participants**	Patients with epithelial ovarian carcinoma	**Experimental**	FSS	**Comparator**	RS
**Aim**	assignment to intervention (the 'intention-to-treat' effect)	**Outcome**	CSS	**Result**	The 5-year CSS rates for patients who did and did not receive FSS were 77.5% and 81.3%, respectively (P = 0.523).

**Confounding domains listed in the review protocol**	**Measured variable(s)**	**Is there evidence that controlling for this variable was unnecessary?**	**Is the confounding domain measured validly and reliably by this variable?**	
Age	NA	No	Yes	
Race	NA	No	Yes	
Stage	Pathology report	No	Yes	
Grade	Pathology report	No	Yes	
Histology	Pathology report	No	Yes	
Tomor size	Clinical data	No	Yes	
Elevated serum CA125	Clinical data	No	Yes	
Intraoperative rupture	Clinical data	No	Yes	
Co-morbidities	Clinical data	No	Yes	

**Additional confounding domains**	**Measured variable(s)**	**Is there evidence that controlling for this variable was unnecessary?**	**Is the confounding domain measured validly and reliably by this variable?**	**OPTIONAL: Is failure to adjust for this variable (alone) expected to favour the experimental intervention or the comparator?**
NA	NA	NA	NA	NA

**Co-interventions listed in the review protocol**	**Is there evidence that controlling for this co-intervention was unnecessary (e.g. because it was not administered)?**	**Is presence of this co-intervention likely to favour outcomes in the experimental intervention or the comparator**
Chemotherapy	No	No information

**Additional co-interventions**	**Is there evidence that controlling for this co-intervention was unnecessary (e.g. because it was not administered)?**	**Is presence of this co-intervention likely to favour outcomes in the experimental intervention or the comparator**
NA	NA	NA

**Domain**	**Signalling questions**	**Response options**	**Description**
**Bias due to confounding**	1.1 Is there potential for confounding of the effect of intervention in this study?	Y	
	1.2 If Y/PY to 1.1: Was the analysis based on splitting participants' follow up time according to intervention received?	N	
	1.3 If Y/PY to 1.2: Were intervention discontinuations or switches likely to be related to factors that are prognostic for the outcome?	NA	
	**Questions relating to baseline confounding only**		
	1.4 If N/PN to 1.2 or 1.3: Did the authors use an appropriate analysis method that controlled for all the important confounding domains?	N	As presented in Table 2, patients who received chemotherapy were younger (P = 0.001), with higher grade (P= 0.017) and with larger tumor size (P < 0.001).
	1.5 If Y/PY to 1.4: Were confounding domains that were controlled for measured validly and reliably by the variables available in this study?	NA	
	1.6. If N/PN to 1.2 or 1.3: Did the authors control for any post-intervention variables that could have been affected by the intervention?	Y	
	**Questions relating to baseline and time-varying confounding**		
	1.7. If Y/PY to 1.3: Did the authors use an appropriate analysis method that controlled for all the important confounding domains and for time-varying confounding?	NA	
	1.8. If Y/PY to 1.7: Were confounding domains that were controlled for measured validly and reliably by the variables available in this study?	NA	
	**Risk of bias judgement**	**Serious**	
**Bias in selection of participants into the study**	2.1 Was selection of participants into the study (or into the analysis) based on participant characteristics observed after the start of intervention?	N	
	2.2 If Y/PY to 2.1: Were the post-intervention variables that influenced selection likely to be associated with intervention?	NA	
	2.3 If Y/PY to 2.2: Were the post-intervention variables that influenced selection likely to be influenced by the outcome or a cause of the outcome?	NA	
	2.4 If N/PN to 2.1: Do start of follow-up and start of intervention coincide for most participants?	PY	
	2.5. If Y/PY to 2.2 and 2.3, or N/PN to 2.4: Were adjustment techniques used that are likely to correct for the presence of selection biases?	NA	
	**Risk of bias judgement**	**Low**	
**Bias in classification of interventions**	3.1 Were intervention groups clearly defined?	Y	Among patients aged between 18 and 45 years old, we selected those with unilateral salpingo-oophorectomy and uterine preservation (FSS group) and those with bilateral salpingo-oophorectomy or hysterectomy (non-FSS) based on surgery codes of ovarian cancer to perform subgroup analysis.
	3.2 Was the information used to define intervention groups recorded at the start of the intervention?	Y	
	3.3 Could classification of intervention status have been affected by knowledge of the outcome or risk of the outcome?	N	
	**Risk of bias judgement**	**Low**	
**Bias due to deviations from intended interventions**	**If your aim for this study is to assess the effect of assignment to intervention, answer questions 4.1 and 4.2**		
	4.1. Were there deviations from the intended intervention beyond what would be expected in usual practice?	N	There was no significant regarding the proportion of patients receiving chemotherapy (P = 0.479) or LN dissection (P = 0.396) between these two groups (Table 4).
	4.2. If Y/PY to 4.1: Were these deviations from intended intervention unbalanced between groups and likely to have affected the outcome?	NA	
	**If your aim for this study is to assess the effect of starting and adhering to intervention, answer questions 4.3 to 4.6**		
	4.3. Were important co-interventions balanced across intervention groups?	NA	
	4.4. Was the intervention implemented successfully for most participants?	NA	
	4.5. Did study participants adhere to the assigned intervention regimen?	NA	
	4.6. If N/PN to 4.3, 4.4 or 4.5: Was an appropriate analysis used to estimate the effect of starting and adhering to the intervention?	NA	
	**Risk of bias judgement**	**Low**	
**Bias due to missing data**	5.1 Were outcome data available for all, or nearly all, participants?	Y	
	5.2 Were participants excluded due to missing data on intervention status?	PN	
	5.3 Were participants excluded due to missing data on other variables needed for the analysis?	PN	
	5.4 If PN/N to 5.1, or Y/PY to 5.2 or 5.3: Are the proportion of participants and reasons for missing data similar across interventions?	NA	

	5.5 If PN/N to 5.1, or Y/PY to 5.2 or 5.3: Is there evidence that results were robust to the presence of missing data?	NA	
	**Risk of bias judgement**	**Low**	
**Bias in measurement of the outcome**	6.1 Could the outcome measure have been influenced by knowledge of the intervention received?	N	CSS was considered as the time from diagnosis to death due to OCCC.
	6.2 Were outcome assessors aware of the intervention received by study participants?	PY	
	6.3 Were the methods of outcome assessment comparable across intervention groups?	Y	
	6.4 Were any systematic errors in measurement of the outcome related to intervention received?	PN	
	**Risk of bias judgement**	**Low**	
**Bias in selection of the reported result**	Is the reported effect estimate likely to be selected, on the basis of the results, from…		
	7.1. ... multiple outcome measurements within the outcome domain?	PN	
	7.2 ... multiple analyses of the intervention-outcome relationship?	PN	
	7.3 ... different subgroups?	PN	
	**Risk of bias judgement**	**Low**	
**Overall bias**	**Risk of bias judgement**	**Serious**	

**Unique ID**	Chen 2021	**Ref or Label**	Arch Gynecol Obstet. 2021;304(2):521-529.	**Design**	Individually randomized
**Participants**	Patients with epithelial ovarian carcinoma	**Experimental**	FSS	**Comparator**	RS
**Aim**	assignment to intervention (the 'intention-to-treat' effect)	**Outcome**	OS	**Result**	The 5-year OS rates for patients who did and did not receive FSS were 77.5% and 80.6%, respectively (P = 0.523).
**Confounding domains listed in the review protocol**		**Measured variable(s)**	**Is there evidence that controlling for this variable was unnecessary?**	**Is the confounding domain measured validly and reliably by this variable?**	
Age		NA	No	Yes	
Race		NA	No	Yes	
Stage		Pathology report	No	Yes	
Grade		Pathology report	No	Yes	
Histology		Pathology report	No	Yes	
Tomor size		Clinical data	No	Yes	
Elevated serum CA125		Clinical data	No	Yes	
Intraoperative rupture		Clinical data	No	Yes	
Co-morbidities		Clinical data	No	Yes	
**Additional confounding domains**		**Measured variable(s)**	**Is there evidence that controlling for this variable was unnecessary?**	**Is the confounding domain measured validly and reliably by this variable?**	**OPTIONAL: Is failure to adjust for this variable (alone) expected to favour the experimental intervention or the comparator?**
NA		NA	NA	NA	NA
**Co-interventions listed in the review protocol**		**Is there evidence that controlling for this co-intervention was unnecessary (e.g. because it was not administered)?**		**Is presence of this co-intervention likely to favour outcomes in the experimental intervention or the comparator**	
Chemotherapy		No		No information	
**Additional co-interventions**		**Is there evidence that controlling for this co-intervention was unnecessary (e.g. because it was not administered)?**		**Is presence of this co-intervention likely to favour outcomes in the experimental intervention or the comparator**	
NA		NA		NA	

**Domain**	**Signalling questions**	**Response options**	**Description**
**Bias due to confounding**	1.1 Is there potential for confounding of the effect of intervention in this study?	Y	
	1.2 If Y/PY to 1.1: Was the analysis based on splitting participants' follow up time according to intervention received?	N	
	1.3 If Y/PY to 1.2: Were intervention discontinuations or switches likely to be related to factors that are prognostic for the outcome?	NA	
	**Questions relating to baseline confounding only**		
	1.4 If N/PN to 1.2 or 1.3: Did the authors use an appropriate analysis method that controlled for all the important confounding domains?	N	As presented in Table 2, patients who received chemotherapy were younger (P = 0.001), with higher grade (P= 0.017) and with larger tumor size (P < 0.001).
	1.5 If Y/PY to 1.4: Were confounding domains that were controlled for measured validly and reliably by the variables available in this study?	NA	
	1.6. If N/PN to 1.2 or 1.3: Did the authors control for any post-intervention variables that could have been affected by the intervention?	Y	
	**Questions relating to baseline and time-varying confounding**		
	1.7. If Y/PY to 1.3: Did the authors use an appropriate analysis method that controlled for all the important confounding domains and for time-varying confounding?	NA	
	1.8. If Y/PY to 1.7: Were confounding domains that were controlled for measured validly and reliably by the variables available in this study?	NA	
	**Risk of bias judgement**	**Serious**	
**Bias in selection of participants into the study**	2.1 Was selection of participants into the study (or into the analysis) based on participant characteristics observed after the start of intervention?	N	
	2.2 If Y/PY to 2.1: Were the post-intervention variables that influenced selection likely to be associated with intervention?	NA	
	2.3 If Y/PY to 2.2: Were the post-intervention variables that influenced selection likely to be influenced by the outcome or a cause of the outcome?	NA	
	2.4 If N/PN to 2.1: Do start of follow-up and start of intervention coincide for most participants?	PY	
	2.5. If Y/PY to 2.2 and 2.3, or N/PN to 2.4: Were adjustment techniques used that are likely to correct for the presence of selection biases?	NA	
	**Risk of bias judgement**	**Low**	
**Bias in classification of interventions**	3.1 Were intervention groups clearly defined?	Y	Among patients aged between 18 and 45 years old, we selected those with unilateral salpingo-oophorectomy and uterine preservation (FSS group) and those with bilateral salpingo-oophorectomy or hysterectomy (non-FSS) based on surgery codes of ovarian cancer to perform subgroup analysis
	3.2 Was the information used to define intervention groups recorded at the start of the intervention?	Y	
	3.3 Could classification of intervention status have been affected by knowledge of the outcome or risk of the outcome?	N	

	**Risk of bias judgement**	**Low**	
**Bias due to deviations from intended interventions**	**If your aim for this study is to assess the effect of assignment to intervention, answer questions 4.1 and 4.2**		
	4.1. Were there deviations from the intended intervention beyond what would be expected in usual practice?	N	There was no significant regarding the proportion of patients receiving chemotherapy (P = 0.479) or LN dissection (P = 0.396) between these two groups (Table 4).
	4.2. If Y/PY to 4.1: Were these deviations from intended intervention unbalanced between groups and likely to have affected the outcome?	NA	
	**If your aim for this study is to assess the effect of starting and adhering to intervention, answer questions 4.3 to 4.6**		
	4.3. Were important co-interventions balanced across intervention groups?	NA	
	4.4. Was the intervention implemented successfully for most participants?	NA	
	4.5. Did study participants adhere to the assigned intervention regimen?	NA	
	4.6. If N/PN to 4.3, 4.4 or 4.5: Was an appropriate analysis used to estimate the effect of starting and adhering to the intervention?	NA	
	**Risk of bias judgement**	**Low**	
**Bias due to missing data**	5.1 Were outcome data available for all, or nearly all, participants?	Y	
	5.2 Were participants excluded due to missing data on intervention status?	PN	
	5.3 Were participants excluded due to missing data on other variables needed for the analysis?	PN	
	5.4 If PN/N to 5.1, or Y/PY to 5.2 or 5.3: Are the proportion of participants and reasons for missing data similar across interventions?	NA	
	5.5 If PN/N to 5.1, or Y/PY to 5.2 or 5.3: Is there evidence that results were robust to the presence of missing data?	NA	
	**Risk of bias judgement**	**Low**	
**Bias in measurement of the outcome**	6.1 Could the outcome measure have been influenced by knowledge of the intervention received?	N	OS was defined as the time from diagnosis to death for any cause or to the last contact.
	6.2 Were outcome assessors aware of the intervention received by study participants?	PY	
	6.3 Were the methods of outcome assessment comparable across intervention groups?	Y	
	6.4 Were any systematic errors in measurement of the outcome related to intervention received?	PN	
	**Risk of bias judgement**	**Low**	
**Bias in selection of the reported result**	Is the reported effect estimate likely to be selected, on the basis of the results, from…		
	7.1. ... multiple outcome measurements within the outcome domain?	PN	
	7.2 ... multiple analyses of the intervention-outcome relationship?	PN	
	7.3 ... different subgroups?	PN	
	**Risk of bias judgement**	**Low**	
**Overall bias**	**Risk of bias judgement**	**Serious**	

Unique ID	Colombo 1994	Ref or Label	Gynecol Oncol. 1994;55(3 Pt 2):S47-S51.	Design	Individually randomized
**Participants**	Patients with epithelial ovarian carcinoma	**Experimental**	FSS	**Comparator**	RS
**Aim**	assignment to intervention (the 'intention-to-treat' effect)	**Outcome**	Recurrence rate	**Result**	See Table 4 in the original article

Confounding domains listed in the review protocol	Measured variable(s)	Is there evidence that controlling for this variable was unnecessary?	Is the confounding domain measured validly and reliably by this variable?	
Age	NA	No	Yes	
Race	NA	No	Yes	
Stage	Pathology report	No	Yes	
Grade	Pathology report	No	Yes	
Histology	Pathology report	No	Yes	
Tomor size	Clinical data	No	Yes	
Elevated serum CA125	Clinical data	No	Yes	
Intraoperative rupture	Clinical data	No	Yes	
Co-morbidities	Clinical data	No	Yes	
**Additional confounding domains**	**Measured variable(s)**	**Is there evidence that controlling for this variable was unnecessary?**	**Is the confounding domain measured validly and reliably by this variable?**	**OPTIONAL: Is failure to adjust for this variable (alone) expected to favour the experimental intervention or the comparator?**
NA	NA	NA	NA	NA
**Co-interventions listed in the review protocol**	**Is there evidence that controlling for this co-intervention was unnecessary (e.g. because it was not administered)?**		**Is presence of this co-intervention likely to favour outcomes in the experimental intervention or the comparator**	
Chemotherapy	No		No information	
**Additional co-interventions**	**Is there evidence that controlling for this co-intervention was unnecessary (e.g. because it was not administered)?**		**Is presence of this co-intervention likely to favour outcomes in the experimental intervention or the comparator**	
NA	NA		NA	

Domain	Signalling questions	Response options	Description
**Bias due to confounding**	1.1 Is there potential for confounding of the effect of intervention in this study?	Y	
	1.2 If Y/PY to 1.1: Was the analysis based on splitting participants' follow up time according to intervention received?	N	
	1.3 If Y/PY to 1.2: Were intervention discontinuations or switches likely to be related to factors that are prognostic for the outcome?	NA	
	**Questions relating to baseline confounding only**		
	1.4 If N/PN to 1.2 or 1.3: Did the authors use an appropriate analysis method that controlled for all the important confounding domains?	PN	
	1.5 If Y/PY to 1.4: Were confounding domains that were controlled for measured validly and reliably by the variables available in this study?	NA	
	1.6. If N/PN to 1.2 or 1.3: Did the authors control for any post-intervention variables that could have been affected by the intervention?	Y	
	**Questions relating to baseline and time-varying confounding**		
	1.7. If Y/PY to 1.3: Did the authors use an appropriate analysis method that controlled for all the important confounding domains and for time-varying confounding?	NA	
	1.8. If Y/PY to 1.7: Were confounding domains that were controlled for measured validly and reliably by the variables available in this study?	NA	
	**Risk of bias judgement**	**Serious**	
**Bias in selection of participants into the study**	2.1 Was selection of participants into the study (or into the analysis) based on participant characteristics observed after the start of intervention?	N	
	2.2 If Y/PY to 2.1: Were the post-intervention variables that influenced selection likely to be associated with intervention?	NA	
	2.3 If Y/PY to 2.2: Were the post-intervention variables that influenced selection likely to be influenced by the outcome or a cause of the outcome?	NA	
	2.4 If N/PN to 2.1: Do start of follow-up and start of intervention coincide for most participants?	PY	
	2.5. If Y/PY to 2.2 and 2.3, or N/PN to 2.4: Were adjustment techniques used that are likely to correct for the presence of selection biases?	NA	
	**Risk of bias judgement**	**Low**	
**Bias in classification of interventions**	3.1 Were intervention groups clearly defined?	Y	In most cases conservative surgery consisted of unilateral salpingo-oophorectomy with or without biopsy of controlateral ovary.
	3.2 Was the information used to define intervention groups recorded at the start of the intervention?	Y	
	3.3 Could classification of intervention status have been affected by knowledge of the outcome or risk of the outcome?	N	
	**Risk of bias judgement**	**Low**	
**Bias due to deviations from intended interventions**	**If your aim for this study is to assess the effect of assignment to intervention, answer questions 4.1 and 4.2**		
	4.1. Were there deviations from the intended intervention beyond what would be expected in usual practice?	Y	Adjuvant chemotherapy was given to only 16 patients.
	4.2. If Y/PY to 4.1: Were these deviations from intended intervention unbalanced between groups and likely to have affected the outcome?	Y	
	**If your aim for this study is to assess the effect of starting and adhering to intervention, answer questions 4.3 to 4.6**		
	4.3. Were important co-interventions balanced across intervention groups?	NA	
	4.4. Was the intervention implemented successfully for most participants?	NA	
	4.5. Did study participants adhere to the assigned intervention regimen?	NA	
	4.6. If N/PN to 4.3, 4.4 or 4.5: Was an appropriate analysis used to estimate the effect of starting and adhering to the intervention?	NA	
	**Risk of bias judgement**	**Serious**	
**Bias due to missing data**	5.1 Were outcome data available for all, or nearly all, participants?	Y	
	5.2 Were participants excluded due to missing data on intervention status?	PN	
	5.3 Were participants excluded due to missing data on other variables needed for the analysis?	PN	
	5.4 If PN/N to 5.1, or Y/PY to 5.2 or 5.3: Are the proportion of participants and reasons for missing data similar across interventions?	NA	

	5.5 If PN/N to 5.1, or Y/PY to 5.2 or 5.3: Is there evidence that results were robust to the presence of missing data?	NA	
	**Risk of bias judgement**	**Low**	
**Bias in measurement of the outcome**	6.1 Could the outcome measure have been influenced by knowledge of the intervention received?	N	
	6.2 Were outcome assessors aware of the intervention received by study participants?	PY	
	6.3 Were the methods of outcome assessment comparable across intervention groups?	Y	
	6.4 Were any systematic errors in measurement of the outcome related to intervention received?	PN	
	**Risk of bias judgement**	**Low**	
**Bias in selection of the reported result**	Is the reported effect estimate likely to be selected, on the basis of the results, from…		
	7.1. ... multiple outcome measurements within the outcome domain?	PN	
	7.2 ... multiple analyses of the intervention-outcome relationship?	PN	
	7.3 ... different subgroups?	PN	
	**Risk of bias judgement**	**Low**	
**Overall bias**	**Risk of bias judgement**	**Serious**	

**Unique ID**	Ditto 2014	**Ref or Label**	J Gynecol Oncol. 2014;25(4):320-327.	**Design**	Individually randomized
**Participants**	Patients with epithelial ovarian carcinoma	**Experimental**	FSS	**Comparator**	RS
**Aim**	assignment to intervention (the 'intention-to-treat' effect)	**Outcome**	Recurrence rate	**Result**	Seven patients experienced a recurrence (Table 2): four (22%) in the FSS group and three (16%) in the control group (p=not significant)

Confounding domains listed in the review protocol	Measured variable(s)	Is there evidence that controlling for this variable was unnecessary?	Is the confounding domain measured validly and reliably by this variable?	
Age	NA	No	Yes	
Race	NA	No	Yes	
Stage	Pathology report	No	Yes	
Grade	Pathology report	No	Yes	
Histology	Pathology report	No	Yes	
Tomor size	Clinical data	No	Yes	
Elevated serum CA125	Clinical data	No	Yes	
Intraoperative rupture	Clinical data	No	Yes	
Co-morbidities	Clinical data	No	Yes	

Additional confounding domains	Measured variable(s)	Is there evidence that controlling for this variable was unnecessary?	Is the confounding domain measured validly and reliably by this variable?	
NA	NA	NA	NA	

Co-interventions listed in the review protocol	Is there evidence that controlling for this co-intervention was unnecessary (e.g. because it was not administered)?	Is presence of this co-intervention likely to favour outcomes in the experimental intervention or the comparator
Chemotherapy	No	No information

Additional co-interventions	Is there evidence that controlling for this co-intervention was unnecessary (e.g. because it was not administered)?	Is presence of this co-intervention likely to favour outcomes in the experimental intervention or the comparator
NA	NA	NA

Domain	Signalling questions	Response options	Description
**Bias due to confounding**	1.1 Is there potential for confounding of the effect of intervention in this study?	PN	A total of 24 women underwent FSS for eEOC. Eighteen out of these were one-to-one matched and balanced for stage, histologic type, and grading with a group of patients who
	1.2 If Y/PY to 1.1: Was the analysis based on splitting participants' follow up time according to intervention received?	NA	
	1.3 If Y/PY to 1.2: Were intervention discontinuations or switches likely to be related to factors that are prognostic for the outcome?	NA	
	**Questions relating to baseline confounding only**		
	1.4 If N/PN to 1.2 or 1.3: Did the authors use an appropriate analysis method that controlled for all the important confounding domains?	NA	
	1.5 If Y/PY to 1.4: Were confounding domains that were controlled for measured validly and reliably by the variables available in this study?	NA	
	1.6. If N/PN to 1.2 or 1.3: Did the authors control for any post-intervention variables that could have been affected by the intervention?	NA	
	**Questions relating to baseline and time-varying confounding**		
	1.7. If Y/PY to 1.3: Did the authors use an appropriate analysis method that controlled for all the important confounding domains and for time-varying confounding?	NA	
	1.8. If Y/PY to 1.7: Were confounding domains that were controlled for measured validly and reliably by the variables available in this study?	NA	
	**Risk of bias judgement**	**Low**	
**Bias in selection of participants into the study**	2.1 Was selection of participants into the study (or into the analysis) based on participant characteristics observed after the start of intervention?	N	
	2.2 If Y/PY to 2.1: Were the post-intervention variables that influenced selection likely to be associated with intervention?	NA	
	2.3 If Y/PY to 2.2: Were the post-intervention variables that influenced selection likely to be influenced by the outcome or a cause of the outcome?	NA	
	2.4 If N/PN to 2.1: Do start of follow-up and start of intervention coincide for most participants?	PY	
	2.5. If Y/PY to 2.2 and 2.3, or N/PN to 2.4: Were adjustment techniques used that are likely to correct for the presence of selection biases?	NA	
	**Risk of bias judgement**	**Low**	
**Bias in classification of interventions**	3.1 Were intervention groups clearly defined?	Y	
	3.2 Was the information used to define intervention groups recorded at the start of the intervention?	Y	
	3.3 Could classification of intervention status have been affected by knowledge of the outcome or risk of the outcome?	N	
	**Risk of bias judgement**	**Low**	
**Bias due to deviations from intended interventions**	**If your aim for this study is to assess the effect of assignment to intervention, answer questions 4.1 and 4.2**		
	4.1. Were there deviations from the intended intervention beyond what would be expected in usual practice?	N	Adjuvant chemotherapy, P>0.99.
	4.2. If Y/PY to 4.1: Were these deviations from intended intervention unbalanced between groups and likely to have affected the outcome?	NA	
	**If your aim for this study is to assess the effect of starting and adhering to intervention, answer questions 4.3 to 4.6**		
	4.3. Were important co-interventions balanced across intervention groups?	NA	
	4.4. Was the intervention implemented successfully for most participants?	NA	
	4.5. Did study participants adhere to the assigned intervention regimen?	NA	
	4.6. If N/PN to 4.3, 4.4 or 4.5: Was an appropriate analysis used to estimate the effect of starting and adhering to the intervention?	NA	
	**Risk of bias judgement**	**Low**	
**Bias due to missing data**	5.1 Were outcome data available for all, or nearly all, participants?	Y	
	5.2 Were participants excluded due to missing data on intervention status?	PN	
	5.3 Were participants excluded due to missing data on other variables needed for the analysis?	PN	
	5.4 If PN/N to 5.1, or Y/PY to 5.2 or 5.3: Are the proportion of participants and reasons for missing data similar across interventions?	NA	

	5.5 If PN/N to 5.1, or Y/PY to 5.2 or 5.3: Is there evidence that results were robust to the presence of missing data?	NA	
	**Risk of bias judgement**	**Low**	
**Bias in measurement of the outcome**	6.1 Could the outcome measure have been influenced by knowledge of the intervention received?	N	
	6.2 Were outcome assessors aware of the intervention received by study participants?	PY	
	6.3 Were the methods of outcome assessment comparable across intervention groups?	Y	Recurrences were diagnosed during regular follow-up visit and confirmed on computed tomographic and/or magnetic resonance imaging scans. Whenever possible, histologic or
	6.4 Were any systematic errors in measurement of the outcome related to intervention received?	PN	
	**Risk of bias judgement**	**Low**	
**Bias in selection of the reported result**	Is the reported effect estimate likely to be selected, on the basis of the results, from…		
	7.1. ... multiple outcome measurements within the outcome domain?	PN	
	7.2 ... multiple analyses of the intervention-outcome relationship?	PN	
	7.3 ... different subgroups?	PN	
	**Risk of bias judgement**	**Low**	
**Overall bias**	**Risk of bias judgement**	**Low**	

**Unique ID**	Ditto 2014	**Ref or Label**	J Gynecol Oncol. 2014;25(4):320-327.	**Design**	Individually randomized
**Participants**	Patients with epithelial ovarian carcinoma	**Experimental**	FSS	**Comparator**	RS
**Aim**	assignment to intervention (the 'intention-to-treat' effect)	**Outcome**	DFS	**Result**	The DFS curves did not show any significant difference between the two groups (p=0.422).

**Confounding domains listed in the review protocol**	**Measured variable(s)**	**Is there evidence that controlling for this variable was unnecessary?**	**Is the confounding domain measured validly and reliably by this variable?**	
Age	NA	No	Yes	
Race	NA	No	Yes	
Stage	Pathology report	No	Yes	
Grade	Pathology report	No	Yes	
Histology	Pathology report	No	Yes	
Tomor size	Clinical data	No	Yes	
Elevated serum CA125	Clinical data	No	Yes	
Intraoperative rupture	Clinical data	No	Yes	
Co-morbidities	Clinical data	No	Yes	
**Additional confounding domains**	**Measured variable(s)**	**Is there evidence that controlling for this variable was unnecessary?**	**Is the confounding domain measured validly and reliably by this variable?**	
NA	NA	NA	NA	

**Co-interventions listed in the review protocol**	**Is there evidence that controlling for this co-intervention was unnecessary (e.g. because it was not administered)?**	**Is presence of this co-intervention likely to favour outcomes in the experimental intervention or the comparator**
Chemotherapy	No	No information
**Additional co-interventions**	**Is there evidence that controlling for this co-intervention was unnecessary (e.g. because it was not administered)?**	**Is presence of this co-intervention likely to favour outcomes in the experimental intervention or the comparator**
NA	NA	NA

**Domain**	**Signalling questions**	**Response options**	**Description**
**Bias due to confounding**	1.1 Is there potential for confounding of the effect of intervention in this study?	PN	A total of 24 women underwent FSS for eEOC. Eighteen out of these were one-to-one matched and balanced for stage, histologic type, and grading with a group of patients who
	1.2 If Y/PY to 1.1: Was the analysis based on splitting participants' follow up time according to intervention received?	NA	
	1.3 If Y/PY to 1.2: Were intervention discontinuations or switches likely to be related to factors that are prognostic for the outcome?	NA	
	**Questions relating to baseline confounding only**		
	1.4 If N/PN to 1.2 or 1.3: Did the authors use an appropriate analysis method that controlled for all the important confounding domains?	NA	
	1.5 If Y/PY to 1.4: Were confounding domains that were controlled for measured validly and reliably by the variables available in this study?	NA	
	1.6. If N/PN to 1.2 or 1.3: Did the authors control for any post-intervention variables that could have been affected by the intervention?	NA	
	**Questions relating to baseline and time-varying confounding**		
	1.7. If Y/PY to 1.3: Did the authors use an appropriate analysis method that controlled for all the important confounding domains and for time-varying confounding?	NA	
	1.8. If Y/PY to 1.7: Were confounding domains that were controlled for measured validly and reliably by the variables available in this study?	NA	
	**Risk of bias judgement**	**Low**	
**Bias in selection of participants into the study**	2.1 Was selection of participants into the study (or into the analysis) based on participant characteristics observed after the start of intervention?	N	
	2.2 If Y/PY to 2.1: Were the post-intervention variables that influenced selection likely to be associated with intervention?	NA	
	2.3 If Y/PY to 2.2: Were the post-intervention variables that influenced selection likely to be influenced by the outcome or a cause of the outcome?	NA	
	2.4 If N/PN to 2.1: Do start of follow-up and start of intervention coincide for most participants?	PY	
	2.5. If Y/PY to 2.2 and 2.3, or N/PN to 2.4: Were adjustment techniques used that are likely to correct for the presence of selection biases?	NA	
	**Risk of bias judgement**	**Low**	
**Bias in classification of interventions**	3.1 Were intervention groups clearly defined?	Y	
	3.2 Was the information used to define intervention groups recorded at the start of the intervention?	Y	
	3.3 Could classification of intervention status have been affected by knowledge of the outcome or risk of the outcome?	N	

	**Risk of bias judgement**	**Low**	
**Bias due to deviations from intended interventions**	**If your aim for this study is to assess the effect of assignment to intervention, answer questions 4.1 and 4.2**		
	4.1. Were there deviations from the intended intervention beyond what would be expected in usual practice?	N	Adjuvant chemotherapy, P>0.99.
	4.2. If Y/PY to 4.1: Were these deviations from intended intervention unbalanced between groups and likely to have affected the outcome?	NA	
	**If your aim for this study is to assess the effect of starting and adhering to intervention, answer questions 4.3 to 4.6**		
	4.3. Were important co-interventions balanced across intervention groups?	NA	
	4.4. Was the intervention implemented successfully for most participants?	NA	
	4.5. Did study participants adhere to the assigned intervention regimen?	NA	
	4.6. If N/PN to 4.3, 4.4 or 4.5: Was an appropriate analysis used to estimate the effect of starting and adhering to the intervention?	NA	
	**Risk of bias judgement**	**Low**	
**Bias due to missing data**	5.1 Were outcome data available for all, or nearly all, participants?	Y	
	5.2 Were participants excluded due to missing data on intervention status?	PN	
	5.3 Were participants excluded due to missing data on other variables needed for the analysis?	PN	
	5.4 If PN/N to 5.1, or Y/PY to 5.2 or 5.3: Are the proportion of participants and reasons for missing data similar across interventions?	NA	
	5.5 If PN/N to 5.1, or Y/PY to 5.2 or 5.3: Is there evidence that results were robust to the presence of missing data?	NA	
	**Risk of bias judgement**	**Low**	
**Bias in measurement of the outcome**	6.1 Could the outcome measure have been influenced by knowledge of the intervention received?	N	
	6.2 Were outcome assessors aware of the intervention received by study participants?	PY	
	6.3 Were the methods of outcome assessment comparable across intervention groups?	Y	
	6.4 Were any systematic errors in measurement of the outcome related to intervention received?	PN	
	**Risk of bias judgement**	**Low**	
**Bias in selection of the reported result**	Is the reported effect estimate likely to be selected, on the basis of the results, from…		
	7.1. ... multiple outcome measurements within the outcome domain?	PN	
	7.2 ... multiple analyses of the intervention-outcome relationship?	PN	
	7.3 ... different subgroups?	PN	
	**Risk of bias judgement**	**Low**	
**Overall bias**	**Risk of bias judgement**	**Low**	

Unique ID	Hedback 2018	Ref or Label	Reprod Biomed Online 2018;37(1):71-76.	Design	Individually randomized
Participants	Patients with epithelial ovarian carcinoma	Experimental	FSS	Comparator	RS
Aim	assignment to intervention (the 'intention-to-treat' effect)	Outcome	DFS	Result	The disease-specific survival was 100% in the FSS group and 91% in the RS group.

Confounding domains listed in the review protocol	Measured variable(s)	Is there evidence that controlling for this variable was unnecessary?	Is the confounding domain measured validly and reliably by this variable?	
Age	NA	No	Yes	
Race	NA	No	Yes	
Stage	Pathology report	No	Yes	
Grade	Pathology report	No	Yes	
Histology	Pathology report	No	Yes	
Tomor size	Clinical data	No	Yes	
Elevated serum CA125	Clinical data	No	Yes	
Intraoperative rupture	Clinical data	No	Yes	
Co-morbidities	Clinical data	No	Yes	

Additional confounding domains	Measured variable(s)	Is there evidence that controlling for this variable was unnecessary?	Is the confounding domain measured validly and reliably by this variable?	OPTIONAL: Is failure to adjust for this variable (alone) expected to favour the experimental intervention or the comparator?
NA	NA	NA	NA	NA

Co-interventions listed in the review protocol	Is there evidence that controlling for this co-intervention was unnecessary (e.g. because it was not administered)?	Is presence of this co-intervention likely to favour outcomes in the experimental intervention or the comparator
Chemotherapy	No	No information

Additional co-interventions	Is there evidence that controlling for this co-intervention was unnecessary (e.g. because it was not administered)?	Is presence of this co-intervention likely to favour outcomes in the experimental intervention or the comparator
NA	NA	NA

Domain	Signalling questions	Response options	Description
Bias due to confounding	1.1 Is there potential for confounding of the effect of intervention in this study?	Y	
	1.2 If Y/PY to 1.1: Was the analysis based on splitting participants' follow up time according to intervention received?	N	
	1.3 If Y/PY to 1.2: Were intervention discontinuations or switches likely to be related to factors that are prognostic for the outcome?	NA	
	**Questions relating to baseline confounding only**		
	1.4 If N/PN to 1.2 or 1.3: Did the authors use an appropriate analysis method that controlled for all the important confounding domains?	Y	There were no significant differences in the baseline data between the two groups except for age.
	1.5 If Y/PY to 1.4: Were confounding domains that were controlled for measured validly and reliably by the variables available in this study?	Y	
	1.6. If N/PN to 1.2 or 1.3: Did the authors control for any post-intervention variables that could have been affected by the intervention?	Y	
	**Questions relating to baseline and time-varying confounding**		
	1.7. If Y/PY to 1.3: Did the authors use an appropriate analysis method that controlled for all the important confounding domains and for time-varying confounding?	NA	
	1.8. If Y/PY to 1.7: Were confounding domains that were controlled for measured validly and reliably by the variables available in this study?	NA	
	**Risk of bias judgement**	**Moderate**	
Bias in selection of participants into the study	2.1 Was selection of participants into the study (or into the analysis) based on participant characteristics observed after the start of intervention?	N	
	2.2 If Y/PY to 2.1: Were the post-intervention variables that influenced selection likely to be associated with intervention?	NA	
	2.3 If Y/PY to 2.2: Were the post-intervention variables that influenced selection likely to be influenced by the outcome or a cause of the outcome?	NA	
	2.4 If N/PN to 2.1: Do start of follow-up and start of intervention coincide for most participants?	PY	
	2.5. If Y/PY to 2.2 and 2.3, or N/PN to 2.4: Were adjustment techniques used that are likely to correct for the presence of selection biases?	NA	
	**Risk of bias judgement**	**Low**	
Bias in classification of interventions	3.1 Were intervention groups clearly defined?	Y	FSS was defined as an operation that resulted in preservation of the uterus and the contralateral ovary. Patients who had undergone a prior hysterectomy and/or removal of the contralateral ovary were not included in the FSS group, and therefore included in the RS group.
	3.2 Was the information used to define intervention groups recorded at the start of the intervention?	Y	
	3.3 Could classification of intervention status have been affected by knowledge of the outcome or risk of the outcome?	N	
	**Risk of bias judgement**	**Low**	
Bias due to deviations from intended interventions	**If your aim for this study is to assess the effect of assignment to intervention, answer questions 4.1 and 4.2**		
	4.1. Were there deviations from the intended intervention beyond what would be expected in usual practice?	Y	Forty-seven per cent (n = 50) of all evaluated patients received adjuvant chemotherapy. The rate of adjuvant treatment was 38% (n = 5) in the FSS group and 48% (n = 45) in the RS group (Table 1).
	4.2. If Y/PY to 4.1: Were these deviations from intended intervention unbalanced between groups and likely to have affected the outcome?	Y	
	**If your aim for this study is to assess the effect of starting and adhering to intervention, answer questions 4.3 to 4.6**		
	4.3. Were important co-interventions balanced across intervention groups?	NA	
	4.4. Was the intervention implemented successfully for most participants?	NA	
	4.5. Did study participants adhere to the assigned intervention regimen?	NA	
	4.6. If N/PN to 4.3, 4.4 or 4.5: Was an appropriate analysis used to estimate the effect of starting and adhering to the intervention?	NA	
	**Risk of bias judgement**	**Moderate**	
Bias due to missing data	5.1 Were outcome data available for all, or nearly all, participants?	Y	The Surveillance, Epidemiology, and End Results (SEER) database was utilized to identify patients diagnosed with stage I FEOC OCCC and MOC between January 2000 and December
	5.2 Were participants excluded due to missing data on intervention status?	PN	
	5.3 Were participants excluded due to missing data on other variables needed for the analysis?	PN	
	5.4 If PN/N to 5.1, or Y/PY to 5.2 or 5.3: Are the proportion of participants and reasons for missing data similar across interventions?	NA	

	5.5 If PN/N to 5.1, or Y/PY to 5.2 or 5.3: Is there evidence that results were robust to the presence of missing data?	NA	
	**Risk of bias judgement**	**Low**	
**Bias in measurement of the outcome**	6.1 Could the outcome measure have been influenced by knowledge of the intervention received?	N	
	6.2 Were outcome assessors aware of the intervention received by study participants?	PY	
	6.3 Were the methods of outcome assessment comparable across intervention groups?	Y	
	6.4 Were any systematic errors in measurement of the outcome related to intervention received?	PN	
	**Risk of bias judgement**	**Low**	
**Bias in selection of the reported result**	Is the reported effect estimate likely to be selected, on the basis of the results, from…		
	7.1. ... multiple outcome measurements within the outcome domain?	PN	
	7.2 ... multiple analyses of the intervention-outcome relationship?	PN	
	7.3 ... different subgroups?	PN	
	**Risk of bias judgement**	**Low**	
**Overall bias**	**Risk of bias judgement**	**Moderate**	

**Unique ID**	Jiang 2017	**Ref or Label**	World Journal of Surgical Oncology 2017;15:154	**Design**	Individually randomized
**Participants**	Patients with epithelial ovarian carcinoma	**Experimental**	FSS	**Comparator**	RS
**Aim**	assignment to intervention (the 'intention-to-treat' effect)	**Outcome**	DFS	**Result**	RS and FSS patients had a 5-year DFS rate of 83.0 and 91.0%, respectively.

Confounding domains listed in the review protocol	Measured variable(s)	Is there evidence that controlling for this variable was unnecessary?	Is the confounding domain measured validly and reliably by this variable?	
Age	NA	No	Yes	
Race	NA	No	Yes	
Stage	Pathology report	No	Yes	
Grade	Pathology report	No	Yes	
Histology	Pathology report	No	Yes	
Tomor size	Clinical data	No	Yes	
Elevated serum CA125	Clinical data	No	Yes	
Intraoperative rupture	Clinical data	No	Yes	
Co-morbidities	Clinical data	No	Yes	

Additional confounding domains	Measured variable(s)	Is there evidence that controlling for this variable was unnecessary?	Is the confounding domain measured validly and reliably by this variable?	OPTIONAL: Is failure to adjust for this variable (alone) expected to favour the experimental intervention or the comparator?
NA	NA	NA	NA	NA

Co-interventions listed in the review protocol	Is there evidence that controlling for this co-intervention was unnecessary (e.g. because it was not administered)?	Is presence of this co-intervention likely to favour outcomes in the experimental intervention or the comparator
Chemotherapy	No	No information

Additional co-interventions	Is there evidence that controlling for this co-intervention was unnecessary (e.g. because it was not administered)?	Is presence of this co-intervention likely to favour outcomes in the experimental intervention or the comparator
NA	NA	NA

Domain	Signalling questions	Response options	Description
**Bias due to confounding**	1.1 Is there potential for confounding of the effect of intervention in this study?	Y	
	1.2 If Y/PY to 1.1: Was the analysis based on splitting participants' follow up time according to intervention received?	N	
	1.3 If Y/PY to 1.2: Were intervention discontinuations or switches likely to be related to factors that are prognostic for the outcome?	NA	
	**Questions relating to baseline confounding only**		
	1.4 If N/PN to 1.2 or 1.3: Did the authors use an appropriate analysis method that controlled for all the important confounding domains?	Y	The median age of patients at diagnosis who underwent FSS was significantly younger (by 10 years), compared with those who underwent RS (p < 0.001). Patients in the RS group were more likely to have coexisting endometriosis (p = 0.006), which could be attributed to the higher proportion of clear-cell
	1.5 If Y/PY to 1.4: Were confounding domains that were controlled for measured validly and reliably by the variables available in this study?	N	
	1.6. If N/PN to 1.2 or 1.3: Did the authors control for any post-intervention variables that could have been affected by the intervention?	Y	
	**Questions relating to baseline and time-varying confounding**		
	1.7. If Y/PY to 1.3: Did the authors use an appropriate analysis method that controlled for all the important confounding domains and for time-varying confounding?	NA	
	1.8. If Y/PY to 1.7: Were confounding domains that were controlled for measured validly and reliably by the variables available in this study?	NA	
	**Risk of bias judgement**	**Serious**	
**Bias in selection of participants into the study**	2.1 Was selection of participants into the study (or into the analysis) based on participant characteristics observed after the start of intervention?	N	Patients with incomplete clinical and pathological or follow-up information and those with disease extending beyond stage I and undergoing FSS were also excluded
	2.2 If Y/PY to 2.1: Were the post-intervention variables that influenced selection likely to be associated with intervention?	NA	
	2.3 If Y/PY to 2.2: Were the post-intervention variables that influenced selection likely to be influenced by the outcome or a cause of the outcome?	NA	
	2.4 If N/PN to 2.1: Do start of follow-up and start of intervention coincide for most participants?	PY	
	2.5. If Y/PY to 2.2 and 2.3, or N/PN to 2.4: Were adjustment techniques used that are likely to correct for the presence of selection biases?	NA	
	**Risk of bias judgement**	**Low**	
**Bias in classification of interventions**	3.1 Were intervention groups clearly defined?	Y	Fertility-sparing surgery included ipsilateral adnexectomy and biopsy or wedge excision of contralateral ovary. Radical surgery included hysterectomy and bilateral adnexectomy. Two independent pathologists with extensive experience in gynecological pathology reviewed all of the pathological slides
	3.2 Was the information used to define intervention groups recorded at the start of the intervention?	Y	
	3.3 Could classification of intervention status have been affected by knowledge of the outcome or risk of the outcome?	N	
	**Risk of bias judgement**	**Low**	
**Bias due to deviations from intended interventions**	**If your aim for this study is to assess the effect of assignment to intervention, answer questions 4.1 and 4.2**		
	4.1. Were there deviations from the intended intervention beyond what would be expected in usual practice?	Y	Because the RS group included more high-grade and clear-cell tumors (p < 0.001) than the FSS group, the proportion of patients receiving adjuvant chemotherapy (p = 0.006) were higher in the RS group.
	4.2. If Y/PY to 4.1: Were these deviations from intended intervention unbalanced between groups and likely to have affected the outcome?	Y	
	**If your aim for this study is to assess the effect of starting and adhering to intervention, answer questions 4.3 to 4.6**		
	4.3. Were important co-interventions balanced across intervention groups?	NA	
	4.4. Was the intervention implemented successfully for most participants?	NA	
	4.5. Did study participants adhere to the assigned intervention regimen?	NA	
	4.6. If N/PN to 4.3, 4.4 or 4.5: Was an appropriate analysis used to estimate the effect of starting and adhering to the intervention?	NA	
	**Risk of bias judgement**	**Serious**	
**Bias due to missing data**	5.1 Were outcome data available for all, or nearly all, participants?	Y	Patients were contacted by telephone or letter to obtain regular follow-up information when it was not available.
	5.2 Were participants excluded due to missing data on intervention status?	PN	
	5.3 Were participants excluded due to missing data on other variables needed for the analysis?	PN	
	5.4 If PN/N to 5.1, or Y/PY to 5.2 or 5.3: Are the proportion of participants and reasons for missing data similar across interventions?	NA	

	5.5 If PN/N to 5.1, or Y/PY to 5.2 or 5.3: Is there evidence that results were robust to the presence of missing data?	NA	
	**Risk of bias judgement**	**Low**	
**Bias in measurement of the outcome**	6.1 Could the outcome measure have been influenced by knowledge of the intervention received?	N	Disease-free survival (DFS) was defined as the time interval from the date of primary surgery to the date of disease recurrence or censoring during the last followup
	6.2 Were outcome assessors aware of the intervention received by study participants?	PY	
	6.3 Were the methods of outcome assessment comparable across intervention groups?	Y	
	6.4 Were any systematic errors in measurement of the outcome related to intervention received?	PN	
	**Risk of bias judgement**	**Low**	
**Bias in selection of the reported result**	Is the reported effect estimate likely to be selected, on the basis of the results, from…		
	7.1. ... multiple outcome measurements within the outcome domain?	PN	
	7.2 ... multiple analyses of the intervention-outcome relationship?	PN	
	7.3 ... different subgroups?	PN	
	**Risk of bias judgement**	**Low**	
**Overall bias**	**Risk of bias judgement**	**Serious**	

**Unique ID**	Jiang 2017	**Ref or Label**	World Journal of Surgical Oncology 2017;15:154	**Design**	Individually randomized
**Participants**	Patients with epithelial ovarian carcinoma	**Experimental**	FSS	**Comparator**	RS
**Aim**	assignment to intervention (the 'intention-to-treat' effect)	**Outcome**	TSS	**Result**	RS and FSS patients had a 5-year TSS rate of 89.3 and 97.3%, respectively.
**Confounding domains listed in the review protocol**		**Measured variable(s)**	**Is there evidence that controlling for this variable was unnecessary?**	**Is the confounding domain measured validly and reliably by this variable?**	
Age		NA	No	Yes	
Race		NA	No	Yes	
Stage		Pathology report	No	Yes	
Grade		Pathology report	No	Yes	
Histology		Pathology report	No	Yes	
Tomor size		Clinical data	No	Yes	
Elevated serum CA125		Clinical data	No	Yes	
Intraoperative rupture		Clinical data	No	Yes	
Co-morbidities		Clinical data	No	Yes	
**Additional confounding domains**		**Measured variable(s)**	**Is there evidence that controlling for this variable was unnecessary?**	**Is the confounding domain measured validly and reliably by this variable?**	**OPTIONAL: Is failure to adjust for this variable (alone) expected to favour the experimental intervention or the comparator?**
NA		NA	NA	NA	NA
**Co-interventions listed in the review protocol**		**Is there evidence that controlling for this co-intervention was unnecessary (e.g. because it was not administered)?**		**Is presence of this co-intervention likely to favour outcomes in the experimental intervention or the comparator**	
Chemotherapy		No		No information	
**Additional co-interventions**		**Is there evidence that controlling for this co-intervention was unnecessary (e.g. because it was not administered)?**		**Is presence of this co-intervention likely to favour outcomes in the experimental intervention or the comparator**	
NA		NA		NA	

**Domain**	**Signalling questions**	**Response options**	**Description**
**Bias due to confounding**	1.1 Is there potential for confounding of the effect of intervention in this study?	Y	
	1.2 If Y/PY to 1.1: Was the analysis based on splitting participants' follow up time according to intervention received?	N	
	1.3 If Y/PY to 1.2: Were intervention discontinuations or switches likely to be related to factors that are prognostic for the outcome?	NA	
	**Questions relating to baseline confounding only**		
	1.4 If N/PN to 1.2 or 1.3: Did the authors use an appropriate analysis method that controlled for all the important confounding domains?	Y	The median age of patients at diagnosis who underwent FSS was significantly younger (by 10 years), compared with those who underwent RS (p < 0.001). Patients in the RS group were more likely to have coexisting endometriosis (p = 0.006), which could be attributed to the higher proportion of clear-cell
	1.5 If Y/PY to 1.4: Were confounding domains that were controlled for measured validly and reliably by the variables available in this study?	N	
	1.6. If N/PN to 1.2 or 1.3: Did the authors control for any post-intervention variables that could have been affected by the intervention?	Y	
	**Questions relating to baseline and time-varying confounding**		
	1.7. If Y/PY to 1.3: Did the authors use an appropriate analysis method that controlled for all the important confounding domains and for time-varying confounding?	NA	
	1.8. If Y/PY to 1.7: Were confounding domains that were controlled for measured validly and reliably by the variables available in this study?	NA	
	**Risk of bias judgement**	**Serious**	
**Bias in selection of participants into the study**	2.1 Was selection of participants into the study (or into the analysis) based on participant characteristics observed after the start of intervention?	N	Patients with incomplete clinical and pathological or follow-up information and those with disease extending beyond stage I and undergoing FSS were also excluded.
	2.2 If Y/PY to 2.1: Were the post-intervention variables that influenced selection likely to be associated with intervention?	NA	
	2.3 If Y/PY to 2.2: Were the post-intervention variables that influenced selection likely to be influenced by the outcome or a cause of the outcome?	NA	
	2.4 If N/PN to 2.1: Do start of follow-up and start of intervention coincide for most participants?	PY	
	2.5. If Y/PY to 2.2 and 2.3, or N/PN to 2.4: Were adjustment techniques used that are likely to correct for the presence of selection biases?	NA	
	**Risk of bias judgement**	**Low**	
**Bias in classification of interventions**	3.1 Were intervention groups clearly defined?	Y	Fertility-sparing surgery included ipsilateral adnexectomy and biopsy or wedge excision of contralateral ovary. Radical surgery included hysterectomy and bilateral adnexectomy. Two independent pathologists with extensive experience in gynecological pathology reviewed all of the pathological slides
	3.2 Was the information used to define intervention groups recorded at the start of the intervention?	Y	
	3.3 Could classification of intervention status have been affected by knowledge of the outcome or risk of the outcome?	N	

	**Risk of bias judgement**	**Low**	
**Bias due to deviations from intended interventions**	**If your aim for this study is to assess the effect of assignment to intervention, answer questions 4.1 and 4.2**		
	4.1. Were there deviations from the intended intervention beyond what would be expected in usual practice?	Y	Because the RS group included more high-grade and clear-cell tumors (p < 0.001) than the FSS group, the proportion of patients receiving adjuvant chemotherapy (p = 0.006) were higher in the RS group. Lymphadenectomy P=0.317
	4.2. If Y/PY to 4.1: Were these deviations from intended intervention unbalanced between groups and likely to have affected the outcome?	Y	
	**If your aim for this study is to assess the effect of starting and adhering to intervention, answer questions 4.3 to 4.6**		
	4.3. Were important co-interventions balanced across intervention groups?	NA	
	4.4. Was the intervention implemented successfully for most participants?	NA	
	4.5. Did study participants adhere to the assigned intervention regimen?	NA	
	4.6. If N/PN to 4.3, 4.4 or 4.5: Was an appropriate analysis used to estimate the effect of starting and adhering to the intervention?	NA	
	**Risk of bias judgement**	**Serious**	
**Bias due to missing data**	5.1 Were outcome data available for all, or nearly all, participants?	Y	Patients were contacted by telephone or letter to obtain regular follow-up information when it was not available.
	5.2 Were participants excluded due to missing data on intervention status?	PN	
	5.3 Were participants excluded due to missing data on other variables needed for the analysis?	PN	
	5.4 If PN/N to 5.1, or Y/PY to 5.2 or 5.3: Are the proportion of participants and reasons for missing data similar across interventions?	NA	
	5.5 If PN/N to 5.1, or Y/PY to 5.2 or 5.3: Is there evidence that results were robust to the presence of missing data?	NA	
	**Risk of bias judgement**	**Low**	
**Bias in measurement of the outcome**	6.1 Could the outcome measure have been influenced by knowledge of the intervention received?	N	Tumor-specific survival (TSS) was defined as the time interval from the date of the primary surgery to the date of death or censoring during the last follow-up
	6.2 Were outcome assessors aware of the intervention received by study participants?	PY	
	6.3 Were the methods of outcome assessment comparable across intervention groups?	Y	
	6.4 Were any systematic errors in measurement of the outcome related to intervention received?	PN	
	**Risk of bias judgement**	**Low**	
**Bias in selection of the reported result**	Is the reported effect estimate likely to be selected, on the basis of the results, from…		
	7.1. ... multiple outcome measurements within the outcome domain?	PN	
	7.2 ... multiple analyses of the intervention-outcome relationship?	PN	
	7.3 ... different subgroups?	PN	
	**Risk of bias judgement**	**Low**	
**Overall bias**	**Risk of bias judgement**	**Serious**	

**Unique ID**	Jiang 2017	**Ref or Label**	World Journal of Surgical Oncology 2017;15:154	**Design**	Individually randomized
**Participants**	Patients with epithelial ovarian carcinoma	**Experimental**	FSS	**Comparator**	RS
**Aim**	assignment to intervention (the 'intention-to-treat' effect)	**Outcome**	Recurrence rate	**Result**	5 of 52 patients in the FSS group had relapses and 9 of 56 patients in the RS group had relapses.
**Confounding domains listed in the review protocol**		**Measured variable(s)**	**Is there evidence that controlling for this variable was unnecessary?**	**Is the confounding domain measured validly and reliably by this variable?**	
Age		NA	No	Yes	
Race		NA	No	Yes	
Stage		Pathology report	No	Yes	
Grade		Pathology report	No	Yes	
Histology		Pathology report	No	Yes	
Tomor size		Clinical data	No	Yes	
Elevated serum CA125		Clinical data	No	Yes	
Intraoperative rupture		Clinical data	No	Yes	
Co-morbidities		Clinical data	No	Yes	
**Additional confounding domains**		**Measured variable(s)**	**Is there evidence that controlling for this variable was unnecessary?**	**Is the confounding domain measured validly and reliably by this variable?**	**OPTIONAL: Is failure to adjust for this variable (alone) expected to favour the experimental intervention or the comparator?**
NA		NA	NA	NA	NA
**Co-interventions listed in the review protocol**		**Is there evidence that controlling for this co-intervention was unnecessary (e.g. because it was not administered)?**		**Is presence of this co-intervention likely to favour outcomes in the experimental intervention or the comparator**	
Chemotherapy		No		No information	
**Additional co-interventions**		**Is there evidence that controlling for this co-intervention was unnecessary (e.g. because it was not administered)?**		**Is presence of this co-intervention likely to favour outcomes in the experimental intervention or the comparator**	
NA		NA		NA	

**Domain**	**Signalling questions**	**Response options**	**Description**
**Bias due to confounding**	1.1 Is there potential for confounding of the effect of intervention in this study?	Y	
	1.2 If Y/PY to 1.1: Was the analysis based on splitting participants' follow up time according to intervention received?	N	
	1.3 If Y/PY to 1.2: Were intervention discontinuations or switches likely to be related to factors that are prognostic for the outcome?	NA	
	**Questions relating to baseline confounding only**		
	1.4 If N/PN to 1.2 or 1.3: Did the authors use an appropriate analysis method that controlled for all the important confounding domains?	Y	The median age of patients at diagnosis who underwent FSS was significantly younger (by 10 years), compared with those who underwent RS (p < 0.001). Patients in the RS group were more likely to have coexisting endometriosis (p = 0.006), which could be attributed to the higher proportion of clear-cell
	1.5 If Y/PY to 1.4: Were confounding domains that were controlled for measured validly and reliably by the variables available in this study?	N	

	1.6. If N/PN to 1.2 or 1.3: Did the authors control for any post-intervention variables that could have been affected by the intervention?	Y	
	**Questions relating to baseline and time-varying confounding**		
	1.7. If Y/PY to 1.3: Did the authors use an appropriate analysis method that controlled for all the important confounding domains and for time-varying confounding?	NA	
	1.8. If Y/PY to 1.7: Were confounding domains that were controlled for measured validly and reliably by the variables available in this study?	NA	
	**Risk of bias judgement**	**Serious**	
**Bias in selection of participants into the study**	2.1 Was selection of participants into the study (or into the analysis) based on participant characteristics observed after the start of intervention?	N	Patients with incomplete clinical and pathological or follow-up information and those with disease extending beyond stage I and undergoing FSS were also excluded.
	2.2 If Y/PY to 2.1: Were the post-intervention variables that influenced selection likely to be associated with intervention?	NA	
	2.3 If Y/PY to 2.2: Were the post-intervention variables that influenced selection likely to be influenced by the outcome or a cause of the outcome?	NA	
	2.4 If N/PN to 2.1: Do start of follow-up and start of intervention coincide for most participants?	PY	
	2.5. If Y/PY to 2.2 and 2.3, or N/PN to 2.4: Were adjustment techniques used that are likely to correct for the presence of selection biases?	NA	
	**Risk of bias judgement**	**Low**	
**Bias in classification of interventions**	3.1 Were intervention groups clearly defined?	Y	Fertility-sparing surgery included ipsilateral adnexectomy and biopsy or wedge excision of contralateral ovary. Radical surgery included hysterectomy and bilateral adnexectomy. Two independent pathologists with extensive experience in gynecological pathology reviewed all of the pathological slides
	3.2 Was the information used to define intervention groups recorded at the start of the intervention?	Y	
	3.3 Could classification of intervention status have been affected by knowledge of the outcome or risk of the outcome?	N	
	**Risk of bias judgement**	**Low**	
**Bias due to deviations from intended interventions**	**If your aim for this study is to assess the effect of assignment to intervention, answer questions 4.1 and 4.2**		
	4.1. Were there deviations from the intended intervention beyond what would be expected in usual practice?	Y	Because the RS group included more high-grade and clear-cell tumors (p < 0.001) than the FSS group, the proportion of patients receiving adjuvant chemotherapy (p = 0.006) were higher in the RS group. Lymphadenectomy P=0.317
	4.2. If Y/PY to 4.1: Were these deviations from intended intervention unbalanced between groups and likely to have affected the outcome?	Y	
	**If your aim for this study is to assess the effect of starting and adhering to intervention, answer questions 4.3 to 4.6**		
	4.3. Were important co-interventions balanced across intervention groups?	NA	
	4.4. Was the intervention implemented successfully for most participants?	NA	
	4.5. Did study participants adhere to the assigned intervention regimen?	NA	
	4.6. If N/PN to 4.3, 4.4 or 4.5: Was an appropriate analysis used to estimate the effect of starting and adhering to the intervention?	NA	
	**Risk of bias judgement**	**Serious**	
**Bias due to missing data**	5.1 Were outcome data available for all, or nearly all, participants?	Y	Patients were contacted by telephone or letter to obtain regular follow-up information when it was not available.
	5.2 Were participants excluded due to missing data on intervention status?	PN	
	5.3 Were participants excluded due to missing data on other variables needed for the analysis?	PN	
	5.4 If PN/N to 5.1, or Y/PY to 5.2 or 5.3: Are the proportion of participants and reasons for missing data similar across interventions?	NA	
	5.5 If PN/N to 5.1, or Y/PY to 5.2 or 5.3: Is there evidence that results were robust to the presence of missing data?	NA	
	**Risk of bias judgement**	**Low**	
**Bias in measurement of the outcome**	6.1 Could the outcome measure have been influenced by knowledge of the intervention received?	N	Recurrence was documented using histologic evidence of disease via tumor biopsy, fine-needle biopsy, or the appearance of new lesions on imaging examination
	6.2 Were outcome assessors aware of the intervention received by study participants?	PY	
	6.3 Were the methods of outcome assessment comparable across intervention groups?	Y	
	6.4 Were any systematic errors in measurement of the outcome related to intervention received?	PN	
	**Risk of bias judgement**	**Low**	
**Bias in selection of the reported result**	Is the reported effect estimate likely to be selected, on the basis of the results, from…		
	7.1. ... multiple outcome measurements within the outcome domain?	PN	
	7.2 ... multiple analyses of the intervention-outcome relationship?	PN	
	7.3 ... different subgroups?	PN	
	**Risk of bias judgement**	**Low**	
**Overall bias**	**Risk of bias judgement**	**Serious**	

**Unique ID**	Jobo 2000	**Ref or Label**	Int J Clin Oncol 2000;5:41–47	**Design**	Individually randomized
**Participants**	Patients with epithelial ovarian carcinoma	**Experimental**	FSS	**Comparator**	RS
**Aim**	assignment to intervention (the 'intention-to-treat' effect)	**Outcome**	OS	**Result**	The 10-year survival rate was 65% for the conservative surgery group and 91% for the radical surgery group.
**Confounding domains listed in the review protocol**		**Measured variable(s)**	**Is there evidence that controlling for this variable was unnecessary?**	**Is the confounding domain measured validly and reliably by this variable?**	
Age		NA	No	Yes	
Race		NA	No	Yes	
Stage		Pathology report	No	Yes	
Grade		Pathology report	No	Yes	
Histology		Pathology report	No	Yes	
Tomor size		Clinical data	No	Yes	
Elevated serum CA125		Clinical data	No	Yes	
Intraoperative rupture		Clinical data	No	Yes	
Co-morbidities		Clinical data	No	Yes	
**Additional confounding domains**		**Measured variable(s)**	**Is there evidence that controlling for this variable was unnecessary?**	**Is the confounding domain measured validly and reliably by this variable?**	**OPTIONAL: Is failure to adjust for this variable (alone) expected to favour the experimental intervention or the comparator?**
NA		NA	NA	NA	NA
**Co-interventions listed in the review protocol**		**Is there evidence that controlling for this co-intervention was unnecessary (e.g. because it was not administered)?**		**Is presence of this co-intervention likely to favour outcomes in the experimental intervention or the comparator**	
Chemotherapy		No		No information	
**Additional co-interventions**		**Is there evidence that controlling for this co-intervention was unnecessary (e.g. because it was not administered)?**		**Is presence of this co-intervention likely to favour outcomes in the experimental intervention or the comparator**	
NA		NA		NA	

**Domain**	**Signalling questions**	**Response options**	**Description**
**Bias due to confounding**	1.1 Is there potential for confounding of the effect of intervention in this study?	Y	
	1.2 If Y/PY to 1.1: Was the analysis based on splitting participants' follow up time according to intervention received?	N	
	1.3 If Y/PY to 1.2: Were intervention discontinuations or switches likely to be related to factors that are prognostic for the outcome?	NA	
	**Questions relating to baseline confounding only**		
	1.4 If N/PN to 1.2 or 1.3: Did the authors use an appropriate analysis method that controlled for all the important confounding domains?	N	
	1.5 If Y/PY to 1.4: Were confounding domains that were controlled for measured validly and reliably by the variables available in this study?	NA	
	1.6. If N/PN to 1.2 or 1.3: Did the authors control for any post-intervention variables that could have been affected by the intervention?	Y	
	**Questions relating to baseline and time-varying confounding**		
	1.7. If Y/PY to 1.3: Did the authors use an appropriate analysis method that controlled for all the important confounding domains and for time-varying confounding?	NA	
	1.8. If Y/PY to 1.7: Were confounding domains that were controlled for measured validly and reliably by the variables available in this study?	NA	
	**Risk of bias judgement**	**Serious**	
**Bias in selection of participants into the study**	2.1 Was selection of participants into the study (or into the analysis) based on participant characteristics observed after the start of intervention?	N	
	2.2 If Y/PY to 2.1: Were the post-intervention variables that influenced selection likely to be associated with intervention?	NA	
	2.3 If Y/PY to 2.2: Were the post-intervention variables that influenced selection likely to be influenced by the outcome or a cause of the outcome?	NA	
	2.4 If N/PN to 2.1: Do start of follow-up and start of intervention coincide for most participants?	PY	
	2.5. If Y/PY to 2.2 and 2.3, or N/PN to 2.4: Were adjustment techniques used that are likely to correct for the presence of selection biases?	NA	
	**Risk of bias judgement**	**Low**	
**Bias in classification of interventions**	3.1 Were intervention groups clearly defined?	Y	Conservative surgical procedures are to preserve fertility (ie, unilateral salpingo-oophorectomy, wedge resection of the contralateral ovary, and omentectomy). Radical operations consist of simple total hysterectomy, bilateral salpingo-oophorectomy, and omentectomy, along with pelvic and
	3.2 Was the information used to define intervention groups recorded at the start of the intervention?	Y	
	3.3 Could classification of intervention status have been affected by knowledge of the outcome or risk of the outcome?	N	
	**Risk of bias judgement**	**Low**	
**Bias due to deviations from intended interventions**	**If your aim for this study is to assess the effect of assignment to intervention, answer questions 4.1 and 4.2**		
	4.1. Were there deviations from the intended intervention beyond what would be expected in usual practice?	N	The treatment of chemotherapy was similar.
	4.2. If Y/PY to 4.1: Were these deviations from intended intervention unbalanced between groups and likely to have affected the outcome?	NA	
	**If your aim for this study is to assess the effect of starting and adhering to intervention, answer questions 4.3 to 4.6**		
	4.3. Were important co-interventions balanced across intervention groups?	NA	
	4.4. Was the intervention implemented successfully for most participants?	NA	
	4.5. Did study participants adhere to the assigned intervention regimen?	NA	
	4.6. If N/PN to 4.3, 4.4 or 4.5: Was an appropriate analysis used to estimate the effect of starting and adhering to the intervention?	NA	
	**Risk of bias judgement**	**Low**	
**Bias due to missing data**	5.1 Were outcome data available for all, or nearly all, participants?	Y	No patients were lost to follow-up.
	5.2 Were participants excluded due to missing data on intervention status?	PN	
	5.3 Were participants excluded due to missing data on other variables needed for the analysis?	PN	
	5.4 If PN/N to 5.1, or Y/PY to 5.2 or 5.3: Are the proportion of participants and reasons for missing data similar across interventions?	NA	

	5.5 If PN/N to 5.1, or Y/PY to 5.2 or 5.3: Is there evidence that results were robust to the presence of missing data?	NA	
	**Risk of bias judgement**	**Low**	
**Bias in measurement of the outcome**	6.1 Could the outcome measure have been influenced by knowledge of the intervention received?	N	
	6.2 Were outcome assessors aware of the intervention received by study participants?	PY	
	6.3 Were the methods of outcome assessment comparable across intervention groups?	Y	
	6.4 Were any systematic errors in measurement of the outcome related to intervention received?	PN	
	**Risk of bias judgement**	**Low**	
**Bias in selection of the reported result**	Is the reported effect estimate likely to be selected, on the basis of the results, from…		
	7.1. ... multiple outcome measurements within the outcome domain?	PN	
	7.2 ... multiple analyses of the intervention-outcome relationship?	PN	
	7.3 ... different subgroups?	PN	
	**Risk of bias judgement**	**Low**	
**Overall bias**	**Risk of bias judgement**	**Serious**	

Unique ID	Johansen 2020	Ref or Label	BMC Cancer 2020;20:1009	Design	Individually randomized
Participants	Patients with epithelial ovarian carcinoma	Experimental	FSS	Comparator	RS
Aim	assignment to intervention (the 'intention-to-treat' effect)	Outcome	DFS	Result	The 5-year DFS rate was 93 and 82% for FSS and RS, respectively.

Confounding domains listed in the review protocol	Measured variable(s)	Is there evidence that controlling for this variable was unnecessary?	Is the confounding domain measured validly and reliably by this variable?	
Age	NA	No	Yes	
Race	NA	No	Yes	
Stage	Pathology report	No	Yes	
Grade	Pathology report	No	Yes	
Histology	Pathology report	No	Yes	
Tomor size	Clinical data	No	Yes	
Elevated serum CA125	Clinical data	No	Yes	
Intraoperative rupture	Clinical data	No	Yes	
Co-morbidities	Clinical data	No	Yes	

Additional confounding domains	Measured variable(s)	Is there evidence that controlling for this variable was unnecessary?	Is the confounding domain measured validly and reliably by this variable?	OPTIONAL: Is failure to adjust for this variable (alone) expected to favour the experimental intervention or the comparator?
NA	NA	NA	NA	NA

Co-interventions listed in the review protocol	Is there evidence that controlling for this co-intervention was unnecessary (e.g. because it was not administered)?	Is presence of this co-intervention likely to favour outcomes in the experimental intervention or the comparator
Chemotherapy	No	No information

Additional co-interventions	Is there evidence that controlling for this co-intervention was unnecessary (e.g. because it was not administered)?	Is presence of this co-intervention likely to favour outcomes in the experimental intervention or the comparator
NA	NA	NA

Domain	Signalling questions	Response options	Description
Bias due to confounding	1.1 Is there potential for confounding of the effect of intervention in this study?	Y	
	1.2 If Y/PY to 1.1: Was the analysis based on splitting participants' follow up time according to intervention received?	N	
	1.3 If Y/PY to 1.2: Were intervention discontinuations or switches likely to be related to factors that are prognostic for the outcome?	NA	
	**Questions relating to baseline confounding only**		
	1.4 If N/PN to 1.2 or 1.3: Did the authors use an appropriate analysis method that controlled for all the important confounding domains?	N	These women were significantly younger than the 47 patients who underwent RS (p < 0.001), and had a lower previous parity (p < 0.001).The women undergoing RS were more often diagnosed in stage IC—62% (29/47) vs. 25% (9/36) (p = 0.002). They also more often had tumors with highly aggressive
	1.5 If Y/PY to 1.4: Were confounding domains that were controlled for measured validly and reliably by the variables available in this study?	NA	
	1.6. If N/PN to 1.2 or 1.3: Did the authors control for any post-intervention variables that could have been affected by the intervention?	Y	
	**Questions relating to baseline and time-varying confounding**		
	1.7. If Y/PY to 1.3: Did the authors use an appropriate analysis method that controlled for all the important confounding domains and for time-varying confounding?	NA	
	1.8. If Y/PY to 1.7: Were confounding domains that were controlled for measured validly and reliably by the variables available in this study?	NA	
	**Risk of bias judgement**	**Serious**	
Bias in selection of participants into the study	2.1 Was selection of participants into the study (or into the analysis) based on participant characteristics observed after the start of intervention?	N	
	2.2 If Y/PY to 2.1: Were the post-intervention variables that influenced selection likely to be associated with intervention?	NA	
	2.3 If Y/PY to 2.2: Were the post-intervention variables that influenced selection likely to be influenced by the outcome or a cause of the outcome?	NA	
	2.4 If N/PN to 2.1: Do start of follow-up and start of intervention coincide for most participants?	PY	
	2.5. If Y/PY to 2.2 and 2.3, or N/PN to 2.4: Were adjustment techniques used that are likely to correct for the presence of selection biases?	NA	
	**Risk of bias judgement**	**Low**	
Bias in classification of interventions	3.1 Were intervention groups clearly defined?	Y	FSS was defined as the preservation of the uterus and at least part of one ovary [16]. RS was defined as hysterectomy with bilateral oophorectomy.
	3.2 Was the information used to define intervention groups recorded at the start of the intervention?	Y	
	3.3 Could classification of intervention status have been affected by knowledge of the outcome or risk of the outcome?	N	
	**Risk of bias judgement**	**Low**	
Bias due to deviations from intended interventions	**If your aim for this study is to assess the effect of assignment to intervention, answer questions 4.1 and 4.2**		
	4.1. Were there deviations from the intended intervention beyond what would be expected in usual practice?	Y	They also more often had tumors with highly aggressive potential, were more often surgically staged with lymph node dissections, and were more likely to receive adjuvant chemotherapy (p = 0.003).
	4.2. If Y/PY to 4.1: Were these deviations from intended intervention unbalanced between groups and likely to have affected the outcome?	Y	
	**If your aim for this study is to assess the effect of starting and adhering to intervention, answer questions 4.3 to 4.6**		
	4.3. Were important co-interventions balanced across intervention groups?	NA	
	4.4. Was the intervention implemented successfully for most participants?	NA	
	4.5. Did study participants adhere to the assigned intervention regimen?	NA	
	4.6. If N/PN to 4.3, 4.4 or 4.5: Was an appropriate analysis used to estimate the effect of starting and adhering to the intervention?	NA	
	**Risk of bias judgement**	**Serious**	
Bias due to missing data	5.1 Were outcome data available for all, or nearly all, participants?	Y	During the study period 83 women aged 18–40 years diagnosed with EOC and with complete data, were identified in the SORGC. There were 11 patients who were excluded due to
	5.2 Were participants excluded due to missing data on intervention status?	PN	
	5.3 Were participants excluded due to missing data on other variables needed for the analysis?	PN	
	5.4 If PN/N to 5.1, or Y/PY to 5.2 or 5.3: Are the proportion of participants and reasons for missing data similar across interventions?	NA	

	5.5 If PN/N to 5.1, or Y/PY to 5.2 or 5.3: Is there evidence that results were robust to the presence of missing data?	NA	
	**Risk of bias judgement**	**Low**	
**Bias in measurement of the outcome**	6.1 Could the outcome measure have been influenced by knowledge of the intervention received?	N	Disease-free survival (DFS), defined as the time from diagnosis to either the first appearance of relapse or the date of death from any cause
	6.2 Were outcome assessors aware of the intervention received by study participants?	PY	
	6.3 Were the methods of outcome assessment comparable across intervention groups?	Y	
	6.4 Were any systematic errors in measurement of the outcome related to intervention received?	PN	
	**Risk of bias judgement**	**Low**	
**Bias in selection of the reported result**	Is the reported effect estimate likely to be selected, on the basis of the results, from…		
	7.1. ... multiple outcome measurements within the outcome domain?	PN	
	7.2 ... multiple analyses of the intervention-outcome relationship?	PN	
	7.3 ... different subgroups?	PN	
	**Risk of bias judgement**	**Low**	
**Overall bias**	**Risk of bias judgement**	**Serious**	

**Unique ID**	Johansen 2020	**Ref or Label**	BMC Cancer 2020;20:1009	**Design**	Individually randomized
**Participants**	Patients with epithelial ovarian carcinoma	**Experimental**	FSS	**Comparator**	RS
**Aim**	assignment to intervention (the 'intention-to-treat' effect)	**Outcome**	OS	**Result**	The 5-year OS rates comparison between FSS and RS were 97 and 89% respectively.

**Confounding domains listed in the review protocol**	**Measured variable(s)**	**Is there evidence that controlling for this variable was unnecessary?**	**Is the confounding domain measured validly and reliably by this variable?**	
Age	NA	No	Yes	
Race	NA	No	Yes	
Stage	Pathology report	No	Yes	
Grade	Pathology report	No	Yes	
Histology	Pathology report	No	Yes	
Tomor size	Clinical data	No	Yes	
Elevated serum CA125	Clinical data	No	Yes	
Intraoperative rupture	Clinical data	No	Yes	
Co-morbidities	Clinical data	No	Yes	
**Additional confounding domains**	**Measured variable(s)**	**Is there evidence that controlling for this variable was unnecessary?**	**Is the confounding domain measured validly and reliably by this variable?**	**OPTIONAL: Is failure to adjust for this variable (alone) expected to favour the experimental intervention or the comparator?**
NA	NA	NA	NA	NA

**Co-interventions listed in the review protocol**	**Is there evidence that controlling for this co-intervention was unnecessary (e.g. because it was not administered)?**	**Is presence of this co-intervention likely to favour outcomes in the experimental intervention or the comparator**
Chemotherapy	No	No information
**Additional co-interventions**	**Is there evidence that controlling for this co-intervention was unnecessary (e.g. because it was not administered)?**	**Is presence of this co-intervention likely to favour outcomes in the experimental intervention or the comparator**
NA	NA	NA

**Domain**	**Signalling questions**	**Response options**	**Description**
**Bias due to confounding**	1.1 Is there potential for confounding of the effect of intervention in this study?	Y	
	1.2 If Y/PY to 1.1: Was the analysis based on splitting participants' follow up time according to intervention received?	N	
	1.3 If Y/PY to 1.2: Were intervention discontinuations or switches likely to be related to factors that are prognostic for the outcome?	NA	
	**Questions relating to baseline confounding only**		
	1.4 If N/PN to 1.2 or 1.3: Did the authors use an appropriate analysis method that controlled for all the important confounding domains?	N	These women were significantly younger than the 47 patients who underwent RS (p < 0.001), and had a lower previous parity (p < 0.001).The women undergoing RS were more often diagnosed in stage IC—62% (29/47) vs. 25% (9/36) (p = 0.002). They also more often had tumors with highly aggressive
	1.5 If Y/PY to 1.4: Were confounding domains that were controlled for measured validly and reliably by the variables available in this study?	NA	
	1.6. If N/PN to 1.2 or 1.3: Did the authors control for any post-intervention variables that could have been affected by the intervention?	Y	
	**Questions relating to baseline and time-varying confounding**		
	1.7. If Y/PY to 1.3: Did the authors use an appropriate analysis method that controlled for all the important confounding domains and for time-varying confounding?	NA	
	1.8. If Y/PY to 1.7: Were confounding domains that were controlled for measured validly and reliably by the variables available in this study?	NA	
	**Risk of bias judgement**	**Serious**	
**Bias in selection of participants into the study**	2.1 Was selection of participants into the study (or into the analysis) based on participant characteristics observed after the start of intervention?	N	
	2.2 If Y/PY to 2.1: Were the post-intervention variables that influenced selection likely to be associated with intervention?	NA	
	2.3 If Y/PY to 2.2: Were the post-intervention variables that influenced selection likely to be influenced by the outcome or a cause of the outcome?	NA	
	2.4 If N/PN to 2.1: Do start of follow-up and start of intervention coincide for most participants?	PY	
	2.5. If Y/PY to 2.2 and 2.3, or N/PN to 2.4: Were adjustment techniques used that are likely to correct for the presence of selection biases?	NA	
	**Risk of bias judgement**	**Low**	
**Bias in classification of interventions**	3.1 Were intervention groups clearly defined?	Y	FSS was defined as the preservation of the uterus and at least part of one ovary [16]. RS was defined as hysterectomy with bilateral oophorectomy.
	3.2 Was the information used to define intervention groups recorded at the start of the intervention?	Y	
	3.3 Could classification of intervention status have been affected by knowledge of the outcome or risk of the outcome?	N	

	**Risk of bias judgement**	**Low**	
**Bias due to deviations from intended interventions**	**If your aim for this study is to assess the effect of assignment to intervention, answer questions 4.1 and 4.2**		
	4.1. Were there deviations from the intended intervention beyond what would be expected in usual practice?	Y	They also more often had tumors with highly aggressive potential, were more often surgically staged with lymph node dissections, and were more likely to receive adjuvant chemotherapy (p = 0.003).
	4.2. If Y/PY to 4.1: Were these deviations from intended intervention unbalanced between groups and likely to have affected the outcome?	Y	
	**If your aim for this study is to assess the effect of starting and adhering to intervention, answer questions 4.3 to 4.6**		
	4.3. Were important co-interventions balanced across intervention groups?	NA	
	4.4. Was the intervention implemented successfully for most participants?	NA	
	4.5. Did study participants adhere to the assigned intervention regimen?	NA	
	4.6. If N/PN to 4.3, 4.4 or 4.5: Was an appropriate analysis used to estimate the effect of starting and adhering to the intervention?	NA	
	**Risk of bias judgement**	**Serious**	
**Bias due to missing data**	5.1 Were outcome data available for all, or nearly all, participants?	Y	During the study period 83 women aged 18–40 years diagnosed with EOC and with complete data, were identified in the SORGC. There were 11 patients who were excluded due to
	5.2 Were participants excluded due to missing data on intervention status?	PN	
	5.3 Were participants excluded due to missing data on other variables needed for the analysis?	PN	
	5.4 If PN/N to 5.1, or Y/PY to 5.2 or 5.3: Are the proportion of participants and reasons for missing data similar across interventions?	NA	
	5.5 If PN/N to 5.1, or Y/PY to 5.2 or 5.3: Is there evidence that results were robust to the presence of missing data?	NA	
	**Risk of bias judgement**	**Low**	
**Bias in measurement of the outcome**	6.1 Could the outcome measure have been influenced by knowledge of the intervention received?	N	Survival estimates included overall survival (OS), calculated from the date of diagnosis to either the date of death from any cause or the date of data retrieval
	6.2 Were outcome assessors aware of the intervention received by study participants?	PY	
	6.3 Were the methods of outcome assessment comparable across intervention groups?	Y	
	6.4 Were any systematic errors in measurement of the outcome related to intervention received?	PN	
	**Risk of bias judgement**	**Low**	
**Bias in selection of the reported result**	Is the reported effect estimate likely to be selected, on the basis of the results, from…		
	7.1. ... multiple outcome measurements within the outcome domain?	PN	
	7.2 ... multiple analyses of the intervention-outcome relationship?	PN	
	7.3 ... different subgroups?	PN	
	**Risk of bias judgement**	**Low**	
**Overall bias**	**Risk of bias judgement**	**Serious**	

**Unique ID**	Li 2023	**Ref or Label**	Eur J Surg Oncol. Published online November 14, 2023.	**Design**	Individually randomized
**Participants**	Patients with epithelial ovarian carcinoma	**Experimental**	FSS	**Comparator**	RS
**Aim**	assignment to intervention (the 'intention-to-treat' effect)	**Outcome**	OS	**Result**	FSS did not significantly affect the 5-year OS of patients with EEOC (94.8% vs. 95.4%, p = 0.687) or MOC (95.9% vs. 92.3%, p = 0.071)

Confounding domains listed in the review protocol	Measured variable(s)	Is there evidence that controlling for this variable was unnecessary?	Is the confounding domain measured validly and reliably by this variable?	
Age	NA	No	Yes	
Race	NA	No	Yes	
Stage	Pathology report	No	Yes	
Grade	Pathology report	No	Yes	
Histology	Pathology report	No	Yes	
Tomor size	Clinical data	No	Yes	
Elevated serum CA125	Clinical data	No	Yes	
Intraoperative rupture	Clinical data	No	Yes	
Co-morbidities	Clinical data	No	Yes	

Additional confounding domains	Measured variable(s)	Is there evidence that controlling for this variable was unnecessary?	Is the confounding domain measured validly and reliably by this variable?	OPTIONAL: Is failure to adjust for this variable (alone) expected to favour the experimental intervention or the comparator?
NA	NA	NA	NA	NA

Co-interventions listed in the review protocol	Is there evidence that controlling for this co-intervention was unnecessary (e.g. because it was not administered)?	Is presence of this co-intervention likely to favour outcomes in the experimental intervention or the comparator
Chemotherapy	No	No information

Additional co-interventions	Is there evidence that controlling for this co-intervention was unnecessary (e.g. because it was not administered)?	Is presence of this co-intervention likely to favour outcomes in the experimental intervention or the comparator
NA	NA	NA

Domain	Signalling questions	Response options	Description
**Bias due to confounding**	1.1 Is there potential for confounding of the effect of intervention in this study?	Y	
	1.2 If Y/PY to 1.1: Was the analysis based on splitting participants' follow up time according to intervention received?	N	
	1.3 If Y/PY to 1.2: Were intervention discontinuations or switches likely to be related to factors that are prognostic for the outcome?	NA	
	**Questions relating to baseline confounding only**		
	1.4 If N/PN to 1.2 or 1.3: Did the authors use an appropriate analysis method that controlled for all the important confounding domains?	Y	Further subgroup analysis according to tumor stage and histological grade did not show a worse OS with FSS in stage I EEOC patients.Further subgroup analysis did not show a worse OS with FSS in stage I MOC patients with any substage or histological grade. In the univariate analysis of the cohort of
	1.5 If Y/PY to 1.4: Were confounding domains that were controlled for measured validly and reliably by the variables available in this study?	Y	
	1.6. If N/PN to 1.2 or 1.3: Did the authors control for any post-intervention variables that could have been affected by the intervention?	Y	
	**Questions relating to baseline and time-varying confounding**		
	1.7. If Y/PY to 1.3: Did the authors use an appropriate analysis method that controlled for all the important confounding domains and for time-varying confounding?	NA	
	1.8. If Y/PY to 1.7: Were confounding domains that were controlled for measured validly and reliably by the variables available in this study?	NA	
	**Risk of bias judgement**	**Moderate**	
**Bias in selection of participants into the study**	2.1 Was selection of participants into the study (or into the analysis) based on participant characteristics observed after the start of intervention?	N	Patients were excluded if they met any of the following criteria: lack of histologic confirmation, stage II-IV or unknown stage, receipt of radiation therapy, recommended surgery not
	2.2 If Y/PY to 2.1: Were the post-intervention variables that influenced selection likely to be associated with intervention?	NA	
	2.3 If Y/PY to 2.2: Were the post-intervention variables that influenced selection likely to be influenced by the outcome or a cause of the outcome?	NA	
	2.4 If N/PN to 2.1: Do start of follow-up and start of intervention coincide for most participants?	PY	
	2.5. If Y/PY to 2.2 and 2.3, or N/PN to 2.4: Were adjustment techniques used that are likely to correct for the presence of selection biases?	NA	
	**Risk of bias judgement**	**Low**	
**Bias in classification of interventions**	3.1 Were intervention groups clearly defined?	Y	For the subgroup analysis, we selected patients who underwent unilateral salpingo-oophorectomy and uterine preservation for the FSS group and patients who underwent bilateral salpingo-oophorectomy or hysterectomy for the non-FSS group based on the surgical codes for ovarian cancer
	3.2 Was the information used to define intervention groups recorded at the start of the intervention?	Y	
	3.3 Could classification of intervention status have been affected by knowledge of the outcome or risk of the outcome?	N	
	**Risk of bias judgement**	**Low**	
**Bias due to deviations from intended interventions**	**If your aim for this study is to assess the effect of assignment to intervention, answer questions 4.1 and 4.2**		
	4.1. Were there deviations from the intended intervention beyond what would be expected in usual practice?	Y	In the univariate analysis of the cohort of EEOC, age and lymph node dissection were associated with OS (p < 0.05). In the multivariate analysis, younger age (HR 0.942; 95% CI: 0.919–0.965; P = 0.000) and lymph node dissection (HR 0.494; 95% CI: 0.310–0.785;P = 0.003) were associated with improved OS
	4.2. If Y/PY to 4.1: Were these deviations from intended intervention unbalanced between groups and likely to have affected the outcome?	Y	
	**If your aim for this study is to assess the effect of starting and adhering to intervention, answer questions 4.3 to 4.6**		
	4.3. Were important co-interventions balanced across intervention groups?	NA	
	4.4. Was the intervention implemented successfully for most participants?	NA	
	4.5. Did study participants adhere to the assigned intervention regimen?	NA	
	4.6. If N/PN to 4.3, 4.4 or 4.5: Was an appropriate analysis used to estimate the effect of starting and adhering to the intervention?	NA	
	**Risk of bias judgement**	**Serious**	
**Bias due to missing data**	5.1 Were outcome data available for all, or nearly all, participants?	Y	The Surveillance, Epidemiology, and End Results (SEER) database was utilized to identify patients diagnosed with stage I EEOC,OCCC, and MOC between January 2000 and December
	5.2 Were participants excluded due to missing data on intervention status?	PN	
	5.3 Were participants excluded due to missing data on other variables needed for the analysis?	PN	
	5.4 If PN/N to 5.1, or Y/PY to 5.2 or 5.3: Are the proportion of participants and reasons for missing data similar across interventions?	NA	

	5.5 If PN/N to 5.1, or Y/PY to 5.2 or 5.3: Is there evidence that results were robust to the presence of missing data?	NA	
	**Risk of bias judgement**	**Low**	
**Bias in measurement of the outcome**	6.1 Could the outcome measure have been influenced by knowledge of the intervention received?	N	
	6.2 Were outcome assessors aware of the intervention received by study participants?	PY	
	6.3 Were the methods of outcome assessment comparable across intervention groups?	Y	
	6.4 Were any systematic errors in measurement of the outcome related to intervention received?	PN	
	**Risk of bias judgement**	**Low**	
**Bias in selection of the reported result**	Is the reported effect estimate likely to be selected, on the basis of the results, from…		
	7.1. ... multiple outcome measurements within the outcome domain?	PN	
	7.2 ... multiple analyses of the intervention-outcome relationship?	PN	
	7.3 ... different subgroups?	PN	
	**Risk of bias judgement**	**Low**	
**Overall bias**	**Risk of bias judgement**	**Serious**	

**Unique ID**	Lin 2022	**Ref or Label**	Front Oncol. 2022;12:856818.	**Design**	Individually randomized
**Participants**	Patients with epithelial ovarian carcinoma	**Experimental**	FSS	**Comparator**	RS
**Aim**	assignment to intervention (the 'intention-to-treat' effect)	**Outcome**	DFS	**Result**	The 5-year DFS rates for the whole cohort, FSS group, and RS group were 88.6%, 82.5%, and 94.5% (P=0.207), respectively, and the corresponding 5-year OS rates were 99.3%, 98.6% and

Confounding domains listed in the review protocol	Measured variable(s)	Is there evidence that controlling for this variable was unnecessary?	Is the confounding domain measured validly and reliably by this variable?	
Age	NA	No	Yes	
Race	NA	No	Yes	
Stage	Pathology report	No	Yes	
Grade	Pathology report	No	Yes	
Histology	Pathology report	No	Yes	
Tomor size	Clinical data	No	Yes	
Elevated serum CA125	Clinical data	No	Yes	
Intraoperative rupture	Clinical data	No	Yes	
Co-morbidities	Clinical data	No	Yes	
**Additional confounding domains**	**Measured variable(s)**	**Is there evidence that controlling for this variable was unnecessary?**	**Is the confounding domain measured validly and reliably by this variable?**	
NA	NA	NA	NA	NA

Co-interventions listed in the review protocol	Is there evidence that controlling for this co-intervention was unnecessary (e.g. because it was not administered)?	Is presence of this co-intervention likely to favour outcomes in the experimental intervention or the comparator
Chemotherapy	No	No information
**Additional co-interventions**	**Is there evidence that controlling for this co-intervention was unnecessary (e.g. because it was not administered)?**	**Is presence of this co-intervention likely to favour outcomes in the experimental intervention or the comparator**
NA	NA	NA

Domain	Signalling questions	Response options	Description
Bias due to confounding	1.1 Is there potential for confounding of the effect of intervention in this study?	N	There was no significant difference between the FSS group and RS group regarding preoperative CA125 value, frequency of complete staging surgery, distribution of FIGO stage, rate of
	1.2 If Y/PY to 1.1: Was the analysis based on splitting participants' follow up time according to intervention received?	NA	
	1.3 If Y/PY to 1.2: Were intervention discontinuations or switches likely to be related to factors that are prognostic for the outcome?	NA	
	**Questions relating to baseline confounding only**		
	1.4 If N/PN to 1.2 or 1.3: Did the authors use an appropriate analysis method that controlled for all the important confounding domains?	NA	
	1.5 If Y/PY to 1.4: Were confounding domains that were controlled for measured validly and reliably by the variables available in this study?	NA	
	1.6. If N/PN to 1.2 or 1.3: Did the authors control for any post-intervention variables that could have been affected by the intervention?	NA	
	**Questions relating to baseline and time-varying confounding**		
	1.7. If Y/PY to 1.3: Did the authors use an appropriate analysis method that controlled for all the important confounding domains and for time-varying confounding?	NA	
	1.8. If Y/PY to 1.7: Were confounding domains that were controlled for measured validly and reliably by the variables available in this study?	NA	
	**Risk of bias judgement**	**Low**	
Bias in selection of participants into the study	2.1 Was selection of participants into the study (or into the analysis) based on participant characteristics observed after the start of intervention?	N	This retrospective cohort study was carried out using data from the computerized database at Peking Union Medical College Hospital (PUMCH). One hundred and fifty-nine consecutive
	2.2 If Y/PY to 2.1: Were the post-intervention variables that influenced selection likely to be associated with intervention?	NA	
	2.3 If Y/PY to 2.2: Were the post-intervention variables that influenced selection likely to be influenced by the outcome or a cause of the outcome?	NA	
	2.4 If N/PN to 2.1: Do start of follow-up and start of intervention coincide for most participants?	PY	
	2.5. If Y/PY to 2.2 and 2.3, or N/PN to 2.4: Were adjustment techniques used that are likely to correct for the presence of selection biases?	NA	
	**Risk of bias judgement**	**Low**	
Bias in classification of interventions	3.1 Were intervention groups clearly defined?	Y	FSS, defined as conservation of the uterus and at least part of one ovary, included unilateral cystectomy (UC) and unilateral salpingo-oophorectomy (USO). Radical surgery (RS) was defined as bilateral salpingo-oophorectomy or hysterectomy (with unilateral or bilateral salpingo-oophorectomy). All patients
	3.2 Was the information used to define intervention groups recorded at the start of the intervention?	Y	
	3.3 Could classification of intervention status have been affected by knowledge of the outcome or risk of the outcome?	N	
	**Risk of bias judgement**	**Low**	
Bias due to deviations from intended interventions	**If your aim for this study is to assess the effect of assignment to intervention, answer questions 4.1 and 4.2**		
	4.1. Were there deviations from the intended intervention beyond what would be expected in usual practice?	Y	There was no significant difference between the FSS group and RS group regarding rate of chemotherapy. Appendectomy and lymphadenectomy were optional and carried out according to the surgeons' experience and intraoperative findings. One hundred and thirtyfive (84.9%) patients underwent
	4.2. If Y/PY to 4.1: Were these deviations from intended intervention unbalanced between groups and likely to have affected the outcome?	PN	
	**If your aim for this study is to assess the effect of starting and adhering to intervention, answer questions 4.3 to 4.6**		
	4.3. Were important co-interventions balanced across intervention groups?	NA	
	4.4. Was the intervention implemented successfully for most participants?	NA	
	4.5. Did study participants adhere to the assigned intervention regimen?	NA	
	4.6. If N/PN to 4.3, 4.4 or 4.5: Was an appropriate analysis used to estimate the effect of starting and adhering to the intervention?	NA	
	**Risk of bias judgement**	**Low**	
Bias due to missing data	5.1 Were outcome data available for all, or nearly all, participants?	Y	Patient data were obtained from inpatient and outpatient records, including demographics, clinical features, surgical procedures, pathological findings, adjuvant chemotherapy
	5.2 Were participants excluded due to missing data on intervention status?	PN	
	5.3 Were participants excluded due to missing data on other variables needed for the analysis?	PN	
	5.4 If PN/N to 5.1, or Y/PY to 5.2 or 5.3: Are the proportion of participants and reasons for missing data similar across interventions?	NA	

	5.5 If PN/N to 5.1, or Y/PY to 5.2 or 5.3: Is there evidence that results were robust to the presence of missing data?	NA	
	**Risk of bias judgement**	**Low**	
**Bias in measurement of the outcome**	6.1 Could the outcome measure have been influenced by knowledge of the intervention received?	N	DFS was defined as the time interval from the date of diagnosis to the date of recurrence or censoring.
	6.2 Were outcome assessors aware of the intervention received by study participants?	PY	
	6.3 Were the methods of outcome assessment comparable across intervention groups?	Y	
	6.4 Were any systematic errors in measurement of the outcome related to intervention received?	PN	
	**Risk of bias judgement**	**Low**	
**Bias in selection of the reported result**	Is the reported effect estimate likely to be selected, on the basis of the results, from…		
	7.1. ... multiple outcome measurements within the outcome domain?	PN	
	7.2 ... multiple analyses of the intervention-outcome relationship?	PN	
	7.3 ... different subgroups?	PN	
	**Risk of bias judgement**	**Low**	
**Overall bias**	**Risk of bias judgement**	**Low**	

**Unique ID**	Lin 2022	**Ref or Label**	Front Oncol. 2022;12:856818.	**Design**	Individually randomized
**Participants**	Patients with epithelial ovarian carcinoma	**Experimental**	FSS	**Comparator**	RS
**Aim**	assignment to intervention (the 'intention-to-treat' effect)	**Outcome**	OS	**Result**	The 5-year DFS rates for the whole cohort, FSS group, and RS group were 88.6%, 82.5%, and 94.5% (P=0.207), respectively, and the corresponding 5-year OS rates were 99.3%, 98.6% and

**Confounding domains listed in the review protocol**	**Measured variable(s)**	**Is there evidence that controlling for this variable was unnecessary?**	**Is the confounding domain measured validly and reliably by this variable?**	
Age	NA	No	Yes	
Race	NA	No	Yes	
Stage	Pathology report	No	Yes	
Grade	Pathology report	No	Yes	
Histology	Pathology report	No	Yes	
Tomor size	Clinical data	No	Yes	
Elevated serum CA125	Clinical data	No	Yes	
Intraoperative rupture	Clinical data	No	Yes	
Co-morbidities	Clinical data	No	Yes	

**Additional confounding domains**	**Measured variable(s)**	**Is there evidence that controlling for this variable was unnecessary?**	**Is the confounding domain measured validly and reliably by this variable?**	**OPTIONAL: Is failure to adjust for this variable (alone) expected to favour the experimental intervention or the comparator?**
NA	NA	NA	NA	NA

**Co-interventions listed in the review protocol**	**Is there evidence that controlling for this co-intervention was unnecessary (e.g. because it was not administered)?**	**Is presence of this co-intervention likely to favour outcomes in the experimental intervention or the comparator**
Chemotherapy	No	No information

**Additional co-interventions**	**Is there evidence that controlling for this co-intervention was unnecessary (e.g. because it was not administered)?**	**Is presence of this co-intervention likely to favour outcomes in the experimental intervention or the comparator**
NA	NA	NA

**Domain**	**Signalling questions**	**Response options**	**Description**
**Bias due to confounding**	1.1 Is there potential for confounding of the effect of intervention in this study?	N	There was no significant difference between the FSS group and RS group regarding preoperative CA125 value, frequency of complete staging surgery, distribution of FIGO stage, rate of
	1.2 If Y/PY to 1.1: Was the analysis based on splitting participants' follow up time according to intervention received?	NA	
	1.3 If Y/PY to 1.2: Were intervention discontinuations or switches likely to be related to factors that are prognostic for the outcome?	NA	
	**Questions relating to baseline confounding only**		
	1.4 If N/PN to 1.2 or 1.3: Did the authors use an appropriate analysis method that controlled for all the important confounding domains?	NA	
	1.5 If Y/PY to 1.4: Were confounding domains that were controlled for measured validly and reliably by the variables available in this study?	NA	
	1.6. If N/PN to 1.2 or 1.3: Did the authors control for any post-intervention variables that could have been affected by the intervention?	NA	
	**Questions relating to baseline and time-varying confounding**		
	1.7. If Y/PY to 1.3: Did the authors use an appropriate analysis method that controlled for all the important confounding domains and for time-varying confounding?	NA	
	1.8. If Y/PY to 1.7: Were confounding domains that were controlled for measured validly and reliably by the variables available in this study?	NA	
	**Risk of bias judgement**	**Low**	
**Bias in selection of participants into the study**	2.1 Was selection of participants into the study (or into the analysis) based on participant characteristics observed after the start of intervention?	N	This retrospective cohort study was carried out using data from the computerized database at Peking Union Medical College Hospital (PUMCH). One hundred and fifty-nine consecutive
	2.2 If Y/PY to 2.1: Were the post-intervention variables that influenced selection likely to be associated with intervention?	NA	
	2.3 If Y/PY to 2.2: Were the post-intervention variables that influenced selection likely to be influenced by the outcome or a cause of the outcome?	NA	
	2.4 If N/PN to 2.1: Do start of follow-up and start of intervention coincide for most participants?	PY	
	2.5. If Y/PY to 2.2 and 2.3, or N/PN to 2.4: Were adjustment techniques used that are likely to correct for the presence of selection biases?	NA	
	**Risk of bias judgement**	**Low**	
**Bias in classification of interventions**	3.1 Were intervention groups clearly defined?	Y	FSS, defined as conservation of the uterus and at least part of one ovary, included unilateral cystectomy (UC) and unilateral salpingo-oophorectomy (USO). Radical surgery (RS) was defined as bilateral salpingo-oophorectomy or hysterectomy (with unilateral or bilateral salpingo-oophorectomy). All patients
	3.2 Was the information used to define intervention groups recorded at the start of the intervention?	Y	
	3.3 Could classification of intervention status have been affected by knowledge of the outcome or risk of the outcome?	N	

	**Risk of bias judgement**	**Low**	
**Bias due to deviations from intended interventions**	**If your aim for this study is to assess the effect of assignment to intervention, answer questions 4.1 and 4.2**		
	4.1. Were there deviations from the intended intervention beyond what would be expected in usual practice?	Y	There was no significant difference between the FSS group and RS group regarding rate of chemotherapy. Appendectomy and lymphadenectomy were optional and carried out according to the surgeons' experience and intraoperative findings. One hundred and thirtyfive (84.9%) patients underwent
	4.2. If Y/PY to 4.1: Were these deviations from intended intervention unbalanced between groups and likely to have affected the outcome?	PN	
	**If your aim for this study is to assess the effect of starting and adhering to intervention, answer questions 4.3 to 4.6**		
	4.3. Were important co-interventions balanced across intervention groups?	NA	
	4.4. Was the intervention implemented successfully for most participants?	NA	
	4.5. Did study participants adhere to the assigned intervention regimen?	NA	
	4.6. If N/PN to 4.3, 4.4 or 4.5: Was an appropriate analysis used to estimate the effect of starting and adhering to the intervention?	NA	
	**Risk of bias judgement**	**Low**	
**Bias due to missing data**	5.1 Were outcome data available for all, or nearly all, participants?	Y	Patient data were obtained from inpatient and outpatient records, including demographics, clinical features, surgical procedures, pathological findings, adjuvant chemotherapy
	5.2 Were participants excluded due to missing data on intervention status?	PN	
	5.3 Were participants excluded due to missing data on other variables needed for the analysis?	PN	
	5.4 If PN/N to 5.1, or Y/PY to 5.2 or 5.3: Are the proportion of participants and reasons for missing data similar across interventions?	NA	
	5.5 If PN/N to 5.1, or Y/PY to 5.2 or 5.3: Is there evidence that results were robust to the presence of missing data?	NA	
	**Risk of bias judgement**	**Low**	
**Bias in measurement of the outcome**	6.1 Could the outcome measure have been influenced by knowledge of the intervention received?	N	OS was calculated as the time interval from the date of diagnosis to the date of death or censoring.
	6.2 Were outcome assessors aware of the intervention received by study participants?	PY	
	6.3 Were the methods of outcome assessment comparable across intervention groups?	Y	
	6.4 Were any systematic errors in measurement of the outcome related to intervention received?	PN	
	**Risk of bias judgement**	**Low**	
**Bias in selection of the reported result**	Is the reported effect estimate likely to be selected, on the basis of the results, from…		
	7.1. ... multiple outcome measurements within the outcome domain?	PN	
	7.2 ... multiple analyses of the intervention-outcome relationship?	PN	
	7.3 ... different subgroups?	PN	
	**Risk of bias judgement**	**Low**	
**Overall bias**	**Risk of bias judgement**	**Low**	

**Unique ID**	Lin 2022	**Ref or Label**	Front Oncol. 2022;12:856818.	**Design**	Individually randomized
**Participants**	Patients with epithelial ovarian carcinoma	**Experimental**	FSS	**Comparator**	RS
**Aim**	assignment to intervention (the 'intention-to-treat' effect)	**Outcome**	Recurrence rate	**Result**	Eighteen (11.3%) patients developed at least one recurrence, including 12 (15.4%) patients in the FSS group and six (7.4%) in the RS group (P=0.112). The median times to recurrence

**Confounding domains listed in the review protocol**	**Measured variable(s)**	**Is there evidence that controlling for this variable was unnecessary?**	**Is the confounding domain measured validly and reliably by this variable?**	
Age	NA	No	Yes	
Race	NA	No	Yes	
Stage	Pathology report	No	Yes	
Grade	Pathology report	No	Yes	
Histology	Pathology report	No	Yes	
Tomor size	Clinical data	No	Yes	
Elevated serum CA125	Clinical data	No	Yes	
Intraoperative rupture	Clinical data	No	Yes	
Co-morbidities	Clinical data	No	Yes	

**Additional confounding domains**	**Measured variable(s)**	**Is there evidence that controlling for this variable was unnecessary?**	**Is the confounding domain measured validly and reliably by this variable?**	**OPTIONAL: Is failure to adjust for this variable (alone) expected to favour the experimental intervention or the comparator?**
NA	NA	NA	NA	NA

**Co-interventions listed in the review protocol**	**Is there evidence that controlling for this co-intervention was unnecessary (e.g. because it was not administered)?**	**Is presence of this co-intervention likely to favour outcomes in the experimental intervention or the comparator**
Chemotherapy	No	No information

**Additional co-interventions**	**Is there evidence that controlling for this co-intervention was unnecessary (e.g. because it was not administered)?**	**Is presence of this co-intervention likely to favour outcomes in the experimental intervention or the comparator**
NA	NA	NA

**Domain**	**Signalling questions**	**Response options**	**Description**
**Bias due to confounding**	1.1 Is there potential for confounding of the effect of intervention in this study?	N	There was no significant difference between the FSS group and RS group regarding preoperative CA125 value, frequency of complete staging surgery, distribution of FIGO stage, rate of
	1.2 If Y/PY to 1.1: Was the analysis based on splitting participants' follow up time according to intervention received?	NA	
	1.3 If Y/PY to 1.2: Were intervention discontinuations or switches likely to be related to factors that are prognostic for the outcome?	NA	
	**Questions relating to baseline confounding only**		
	1.4 If N/PN to 1.2 or 1.3: Did the authors use an appropriate analysis method that controlled for all the important confounding domains?	NA	
	1.5 If Y/PY to 1.4: Were confounding domains that were controlled for measured validly and reliably by the variables available in this study?	NA	

	1.6. If N/PN to 1.2 or 1.3: Did the authors control for any post-intervention variables that could have been affected by the intervention?	NA	
	**Questions relating to baseline and time-varying confounding**		
	1.7. If Y/PY to 1.3: Did the authors use an appropriate analysis method that controlled for all the important confounding domains and for time-varying confounding?	NA	
	1.8. If Y/PY to 1.7: Were confounding domains that were controlled for measured validly and reliably by the variables available in this study?	NA	
	**Risk of bias judgement**	**Low**	
**Bias in selection of participants into the study**	2.1 Was selection of participants into the study (or into the analysis) based on participant characteristics observed after the start of intervention?	N	This retrospective cohort study was carried out using data from the computerized database at Peking Union Medical College Hospital (PUMCH). One hundred and fifty-nine consecutive
	2.2 If Y/PY to 2.1: Were the post-intervention variables that influenced selection likely to be associated with intervention?	NA	
	2.3 If Y/PY to 2.2: Were the post-intervention variables that influenced selection likely to be influenced by the outcome or a cause of the outcome?	NA	
	2.4 If N/PN to 2.1: Do start of follow-up and start of intervention coincide for most participants?	PY	
	2.5. If Y/PY to 2.2 and 2.3, or N/PN to 2.4: Were adjustment techniques used that are likely to correct for the presence of selection biases?	NA	
	**Risk of bias judgement**	**Low**	
**Bias in classification of interventions**	3.1 Were intervention groups clearly defined?	Y	FSS, defined as conservation of the uterus and at least part of one ovary, included unilateral cystectomy (UC) and unilateral salpingo-oophorectomy (USO). Radical surgery (RS) was defined as bilateral salpingo-oophorectomy or hysterectomy (with unilateral or bilateral salpingo-oophorectomy). All patients
	3.2 Was the information used to define intervention groups recorded at the start of the intervention?	Y	
	3.3 Could classification of intervention status have been affected by knowledge of the outcome or risk of the outcome?	N	
	**Risk of bias judgement**	**Low**	
**Bias due to deviations from intended interventions**	**If your aim for this study is to assess the effect of assignment to intervention, answer questions 4.1 and 4.2**		
	4.1. Were there deviations from the intended intervention beyond what would be expected in usual practice?	Y	There was no significant difference between the FSS group and RS group regarding rate of chemotherapy.
	4.2. If Y/PY to 4.1: Were these deviations from intended intervention unbalanced between groups and likely to have affected the outcome?	PN	
	**If your aim for this study is to assess the effect of starting and adhering to intervention, answer questions 4.3 to 4.6**		
	4.3. Were important co-interventions balanced across intervention groups?	NA	
	4.4. Was the intervention implemented successfully for most participants?	NA	
	4.5. Did study participants adhere to the assigned intervention regimen?	NA	
	4.6. If N/PN to 4.3, 4.4 or 4.5: Was an appropriate analysis used to estimate the effect of starting and adhering to the intervention?	NA	
	**Risk of bias judgement**	**Low**	
**Bias due to missing data**	5.1 Were outcome data available for all, or nearly all, participants?	Y	Patient data were obtained from inpatient and outpatient records, including demographics, clinical features, surgical procedures, pathological findings, adjuvant chemotherapy
	5.2 Were participants excluded due to missing data on intervention status?	PN	
	5.3 Were participants excluded due to missing data on other variables needed for the analysis?	PN	
	5.4 If PN/N to 5.1, or Y/PY to 5.2 or 5.3: Are the proportion of participants and reasons for missing data similar across interventions?	NA	
	5.5 If PN/N to 5.1, or Y/PY to 5.2 or 5.3: Is there evidence that results were robust to the presence of missing data?	NA	
	**Risk of bias judgement**	**Low**	
**Bias in measurement of the outcome**	6.1 Could the outcome measure have been influenced by knowledge of the intervention received?	N	
	6.2 Were outcome assessors aware of the intervention received by study participants?	PY	
	6.3 Were the methods of outcome assessment comparable across intervention groups?	Y	
	6.4 Were any systematic errors in measurement of the outcome related to intervention received?	PN	
	**Risk of bias judgement**	**Low**	
**Bias in selection of the reported result**	Is the reported effect estimate likely to be selected, on the basis of the results, from…		
	7.1. ... multiple outcome measurements within the outcome domain?	PN	
	7.2 ... multiple analyses of the intervention-outcome relationship?	PN	
	7.3 ... different subgroups?	PN	
	**Risk of bias judgement**	**Low**	
**Overall bias**	**Risk of bias judgement**	**Low**	

**Unique ID**	Nasioudis 2022	**Ref or Label**	Int J Gynecol Cancer. 2022;32(2):165-171.	**Design**	Individually randomized
**Participants**	Patients with epithelial ovarian carcinoma	**Experimental**	FSS	**Comparator**	RS
**Aim**	assignment to intervention (the 'intention-to-treat' effect)	**Outcome**	OS	**Result**	There was no difference in overall survival between the fertility-sparing and radical surgery groups (p=0.37; 5-year overall survival rates 90.2% and 85%, respectively).

**Confounding domains listed in the review protocol**	**Measured variable(s)**	**Is there evidence that controlling for this variable was unnecessary?**	**Is the confounding domain measured validly and reliably by this variable?**	
Age	NA	No	Yes	
Race	NA	No	Yes	
Stage	Pathology report	No	Yes	
Grade	Pathology report	No	Yes	
Histology	Pathology report	No	Yes	
Tomor size	Clinical data	No	Yes	
Elevated serum CA125	Clinical data	No	Yes	
Intraoperative rupture	Clinical data	No	Yes	
Co-morbidities	Clinical data	No	Yes	
**Additional confounding domains**	**Measured variable(s)**	**Is there evidence that controlling for this variable was unnecessary?**	**Is the confounding domain measured validly and reliably by this variable?**	**OPTIONAL: Is failure to adjust for this variable (alone) expected to favour the experimental intervention or the comparator?**
NA	NA	NA	NA	NA

**Co-interventions listed in the review protocol**	**Is there evidence that controlling for this co-intervention was unnecessary (e.g. because it was not administered)?**	**Is presence of this co-intervention likely to favour outcomes in the experimental intervention or the comparator**
Chemotherapy	No	No information
**Additional co-interventions**	**Is there evidence that controlling for this co-intervention was unnecessary (e.g. because it was not administered)?**	**Is presence of this co-intervention likely to favour outcomes in the experimental intervention or the comparator**
NA	NA	NA

**Domain**	**Signalling questions**	**Response options**	**Description**
**Bias due to confounding**	1.1 Is there potential for confounding of the effect of intervention in this study?	Y	
	1.2 If Y/PY to 1.1: Was the analysis based on splitting participants' follow up time according to intervention received?	N	
	1.3 If Y/PY to 1.2: Were intervention discontinuations or switches likely to be related to factors that are prognostic for the outcome?	NA	
	**Questions relating to baseline confounding only**		
	1.4 If N/PN to 1.2 or 1.3: Did the authors use an appropriate analysis method that controlled for all the important confounding domains?	Y	RACE: There were no differences between the two groups in terms of race (p=0.07). CO-MORBIDITIES: There were no differences between the two groups in terms of presence of co-morbidities (p=0.17). GRADE: After controlling for tumor grade and performance of lymphadenectomy, fertility-sparing surgery
	1.5 If Y/PY to 1.4: Were confounding domains that were controlled for measured validly and reliably by the variables available in this study?	Y	
	1.6. If N/PN to 1.2 or 1.3: Did the authors control for any post-intervention variables that could have been affected by the intervention?	Y	
	**Questions relating to baseline and time-varying confounding**		
	1.7. If Y/PY to 1.3: Did the authors use an appropriate analysis method that controlled for all the important confounding domains and for time-varying confounding?	NA	
	1.8. If Y/PY to 1.7: Were confounding domains that were controlled for measured validly and reliably by the variables available in this study?	NA	
	**Risk of bias judgement**	**Moderate**	
**Bias in selection of participants into the study**	2.1 Was selection of participants into the study (or into the analysis) based on participant characteristics observed after the start of intervention?	N	
	2.2 If Y/PY to 2.1: Were the post-intervention variables that influenced selection likely to be associated with intervention?	NA	
	2.3 If Y/PY to 2.2: Were the post-intervention variables that influenced selection likely to be influenced by the outcome or a cause of the outcome?	NA	
	2.4 If N/PN to 2.1: Do start of follow-up and start of intervention coincide for most participants?	PY	
	2.5. If Y/PY to 2.2 and 2.3, or N/PN to 2.4: Were adjustment techniques used that are likely to correct for the presence of selection biases?	NA	
	**Risk of bias judgement**	**Low**	
**Bias in classification of interventions**	3.1 Were intervention groups clearly defined?	Y	In the present study, fertility-sparing surgery was defined as preservation of the uterus and one ovary while radical surgery was defined as hysterectomy with bilateral salpingo-oophorectomy.
	3.2 Was the information used to define intervention groups recorded at the start of the intervention?	Y	
	3.3 Could classification of intervention status have been affected by knowledge of the outcome or risk of the outcome?	N	
	**Risk of bias judgement**	**Low**	
**Bias due to deviations from intended interventions**	**If your aim for this study is to assess the effect of assignment to intervention, answer questions 4.1 and 4.2**		
	4.1. Were there deviations from the intended intervention beyond what would be expected in usual practice?	Y	Patients who had fertility-sparing surgery were less likely to receive chemotherapy (42.9% vs 69.2%, p<0.001).
	4.2. If Y/PY to 4.1: Were these deviations from intended intervention unbalanced between groups and likely to have affected the outcome?	Y	
	**If your aim for this study is to assess the effect of starting and adhering to intervention, answer questions 4.3 to 4.6**		
	4.3. Were important co-interventions balanced across intervention groups?	NA	
	4.4. Was the intervention implemented successfully for most participants?	NA	
	4.5. Did study participants adhere to the assigned intervention regimen?	NA	
	4.6. If N/PN to 4.3, 4.4 or 4.5: Was an appropriate analysis used to estimate the effect of starting and adhering to the intervention?	NA	
	**Risk of bias judgement**	**Serious**	
**Bias due to missing data**	5.1 Were outcome data available for all, or nearly all, participants?	Y	The National Cancer Database was accessed, and a cohort of patients diagnosed between January 2004 and December 2015 with a pathologically confirmed primary ovarian carcinoma
	5.2 Were participants excluded due to missing data on intervention status?	PN	
	5.3 Were participants excluded due to missing data on other variables needed for the analysis?	PN	
	5.4 If PN/N to 5.1, or Y/PY to 5.2 or 5.3: Are the proportion of participants and reasons for missing data similar across interventions?	NA	

	5.5 If PN/N to 5.1, or Y/PY to 5.2 or 5.3: Is there evidence that results were robust to the presence of missing data?	NA	
	**Risk of bias judgement**	**Low**	
**Bias in measurement of the outcome**	6.1 Could the outcome measure have been influenced by knowledge of the intervention received?	N	
	6.2 Were outcome assessors aware of the intervention received by study participants?	PY	
	6.3 Were the methods of outcome assessment comparable across intervention groups?	Y	
	6.4 Were any systematic errors in measurement of the outcome related to intervention received?	PN	
	**Risk of bias judgement**	**Low**	
**Bias in selection of the reported result**	Is the reported effect estimate likely to be selected, on the basis of the results, from…		
	7.1. ... multiple outcome measurements within the outcome domain?	PN	
	7.2 ... multiple analyses of the intervention-outcome relationship?	PN	
	7.3 ... different subgroups?	PN	
	**Risk of bias judgement**	**Low**	
**Overall bias**	**Risk of bias judgement**	**Serious**	

Unique ID	Park 2016	Ref or Label	Int J Gynaecol Obstet 2016;134(1):49-52.	Design	Individually randomized
Participants	Patients with epithelial ovarian carcinoma	Experimental	FSS	Comparator	RS
Aim	assignment to intervention (the 'intention-to-treat' effect)	Outcome	DFS	Result	The 5-year disease-free survival and overall survival of all patients were 81% and 89%, respectively. There was no difference in 5-year disease-free survival (77% vs 84%; P =

Confounding domains listed in the review protocol	Measured variable(s)	Is there evidence that controlling for this variable was unnecessary?	Is the confounding domain measured validly and reliably by this variable?	
Age	NA	No	Yes	
Race	NA	No	Yes	
Stage	Pathology report	No	Yes	
Grade	Pathology report	No	Yes	
Histology	Pathology report	No	Yes	
Tomor size	Clinical data	No	Yes	
Elevated serum CA125	Clinical data	No	Yes	
Intraoperative rupture	Clinical data	No	Yes	
Co-morbidities	Clinical data	No	Yes	

Additional confounding domains	Measured variable(s)	Is there evidence that controlling for this variable was unnecessary?	Is the confounding domain measured validly and reliably by this variable?	OPTIONAL: Is failure to adjust for this variable (alone) expected to favour the experimental intervention or the comparator?
NA	NA	NA	NA	NA

Co-interventions listed in the review protocol	Is there evidence that controlling for this co-intervention was unnecessary (e.g. because it was not administered)?	Is presence of this co-intervention likely to favour outcomes in the experimental intervention or the comparator
Chemotherapy	No	No information

Additional co-interventions	Is there evidence that controlling for this co-intervention was unnecessary (e.g. because it was not administered)?	Is presence of this co-intervention likely to favour outcomes in the experimental intervention or the comparator
NA	NA	NA

Domain	Signalling questions	Response options	Description
Bias due to confounding	1.1 Is there potential for confounding of the effect of intervention in this study?	Y	
	1.2 If Y/PY to 1.1: Was the analysis based on splitting participants' follow up time according to intervention received?	N	
	1.3 If Y/PY to 1.2: Were intervention discontinuations or switches likely to be related to factors that are prognostic for the outcome?	NA	
	**Questions relating to baseline confounding only**		
	1.4 If N/PN to 1.2 or 1.3: Did the authors use an appropriate analysis method that controlled for all the important confounding domains?	Y	Patients who underwent fertility-sparing surgery were significantly younger than were those who underwent radical surgery (P =0.001)(Table 1). More patients in the radical surgery group had a history of abdominal surgery (P = 0.010) and a parity of at least one (P b 0.001), but no other significant
	1.5 If Y/PY to 1.4: Were confounding domains that were controlled for measured validly and reliably by the variables available in this study?	Y	
	1.6. If N/PN to 1.2 or 1.3: Did the authors control for any post-intervention variables that could have been affected by the intervention?	Y	
	**Questions relating to baseline and time-varying confounding**		
	1.7. If Y/PY to 1.3: Did the authors use an appropriate analysis method that controlled for all the important confounding domains and for time-varying confounding?	NA	
	1.8. If Y/PY to 1.7: Were confounding domains that were controlled for measured validly and reliably by the variables available in this study?	NA	
	**Risk of bias judgement**	**Moderate**	
Bias in selection of participants into the study	2.1 Was selection of participants into the study (or into the analysis) based on participant characteristics observed after the start of intervention?	N	
	2.2 If Y/PY to 2.1: Were the post-intervention variables that influenced selection likely to be associated with intervention?	NA	
	2.3 If Y/PY to 2.2: Were the post-intervention variables that influenced selection likely to be influenced by the outcome or a cause of the outcome?	NA	
	2.4 If N/PN to 2.1: Do start of follow-up and start of intervention coincide for most participants?	PY	
	2.5. If Y/PY to 2.2 and 2.3, or N/PN to 2.4: Were adjustment techniques used that are likely to correct for the presence of selection biases?	NA	
	**Risk of bias judgement**	**Low**	
Bias in classification of interventions	3.1 Were intervention groups clearly defined?	Y	
	3.2 Was the information used to define intervention groups recorded at the start of the intervention?	Y	
	3.3 Could classification of intervention status have been affected by knowledge of the outcome or risk of the outcome?	N	
	**Risk of bias judgement**	**Low**	
Bias due to deviations from intended interventions	**If your aim for this study is to assess the effect of assignment to intervention, answer questions 4.1 and 4.2**		
	4.1. Were there deviations from the intended intervention beyond what would be expected in usual practice?	Y	Adjuvant chemotherapy were significantly associated with disease-free and overall survival (data not shown).
	4.2. If Y/PY to 4.1: Were these deviations from intended intervention unbalanced between groups and likely to have affected the outcome?	Y	
	**If your aim for this study is to assess the effect of starting and adhering to intervention, answer questions 4.3 to 4.6**		
	4.3. Were important co-interventions balanced across intervention groups?	NA	
	4.4. Was the intervention implemented successfully for most participants?	NA	
	4.5. Did study participants adhere to the assigned intervention regimen?	NA	
	4.6. If N/PN to 4.3, 4.4 or 4.5: Was an appropriate analysis used to estimate the effect of starting and adhering to the intervention?	NA	
	**Risk of bias judgement**	**Serious**	
Bias due to missing data	5.1 Were outcome data available for all, or nearly all, participants?	Y	
	5.2 Were participants excluded due to missing data on intervention status?	PN	
	5.3 Were participants excluded due to missing data on other variables needed for the analysis?	PN	
	5.4 If PN/N to 5.1, or Y/PY to 5.2 or 5.3: Are the proportion of participants and reasons for missing data similar across interventions?	NA	

	5.5 If PN/N to 5.1, or Y/PY to 5.2 or 5.3: Is there evidence that results were robust to the presence of missing data?	NA	
	**Risk of bias judgement**	**Low**	
**Bias in measurement of the outcome**	6.1 Could the outcome measure have been influenced by knowledge of the intervention received?	N	Disease-free survival time was defined as the time, in months, from the date of surgery to the date of recurrence, date of last follow-up, or censored date
	6.2 Were outcome assessors aware of the intervention received by study participants?	PY	
	6.3 Were the methods of outcome assessment comparable across intervention groups?	Y	
	6.4 Were any systematic errors in measurement of the outcome related to intervention received?	PN	
	**Risk of bias judgement**	**Low**	
**Bias in selection of the reported result**	Is the reported effect estimate likely to be selected, on the basis of the results, from…		
	7.1. ... multiple outcome measurements within the outcome domain?	PN	
	7.2 ... multiple analyses of the intervention-outcome relationship?	PN	
	7.3 ... different subgroups?	PN	
	**Risk of bias judgement**	**Low**	
**Overall bias**	**Risk of bias judgement**	**Serious**	

**Unique ID**	Park 2016	**Ref or Label**	Int J Gynaecol Obstet 2016;134(1):49-52.	**Design**	Individually randomized
**Participants**	Patients with epithelial ovarian carcinoma	**Experimental**	FSS	**Comparator**	RS
**Aim**	assignment to intervention (the 'intention-to-treat' effect)	**Outcome**	OS	**Result**	The 5-year disease-free survival and overall survival of all patients were 81% and 89%, respectively. There was no difference in 5-year disease-free survival (77% vs 84%; P =
**Confounding domains listed in the review protocol**		**Measured variable(s)**	**Is there evidence that controlling for this variable was unnecessary?**	**Is the confounding domain measured validly and reliably by this variable?**	
Age		NA	No	Yes	
Race		NA	No	Yes	
Stage		Pathology report	No	Yes	
Grade		Pathology report	No	Yes	
Histology		Pathology report	No	Yes	
Tomor size		Clinical data	No	Yes	
Elevated serum CA125		Clinical data	No	Yes	
Intraoperative rupture		Clinical data	No	Yes	
Co-morbidities		Clinical data	No	Yes	
**Additional confounding domains**		**Measured variable(s)**	**Is there evidence that controlling for this variable was unnecessary?**	**Is the confounding domain measured validly and reliably by this variable?**	**OPTIONAL: Is failure to adjust for this variable (alone) expected to favour the experimental intervention or the comparator?**
NA		NA	NA	NA	NA
**Co-interventions listed in the review protocol**		**Is there evidence that controlling for this co-intervention was unnecessary (e.g. because it was not administered)?**		**Is presence of this co-intervention likely to favour outcomes in the experimental intervention or the comparator**	
Chemotherapy		No		No information	
**Additional co-interventions**		**Is there evidence that controlling for this co-intervention was unnecessary (e.g. because it was not administered)?**		**Is presence of this co-intervention likely to favour outcomes in the experimental intervention or the comparator**	
NA		NA		NA	

Domain	Signalling questions	Response options	Description
**Bias due to confounding**	1.1 Is there potential for confounding of the effect of intervention in this study?	Y	
	1.2 If Y/PY to 1.1: Was the analysis based on splitting participants' follow up time according to intervention received?	N	
	1.3 If Y/PY to 1.2: Were intervention discontinuations or switches likely to be related to factors that are prognostic for the outcome?	NA	
	**Questions relating to baseline confounding only**		
	1.4 If N/PN to 1.2 or 1.3: Did the authors use an appropriate analysis method that controlled for all the important confounding domains?	Y	Patients who underwent fertility-sparing surgery were significantly younger than were those who underwent radical surgery (P =0.001)(Table 1). More patients in the radical surgery group had a history of abdominal surgery (P = 0.010) and a parity of at least one (P b 0.001), but no other significant
	1.5 If Y/PY to 1.4: Were confounding domains that were controlled for measured validly and reliably by the variables available in this study?	Y	
	1.6. If N/PN to 1.2 or 1.3: Did the authors control for any post-intervention variables that could have been affected by the intervention?	Y	
	**Questions relating to baseline and time-varying confounding**		
	1.7. If Y/PY to 1.3: Did the authors use an appropriate analysis method that controlled for all the important confounding domains and for time-varying confounding?	NA	
	1.8. If Y/PY to 1.7: Were confounding domains that were controlled for measured validly and reliably by the variables available in this study?	NA	
	**Risk of bias judgement**	**Moderate**	
**Bias in selection of participants into the study**	2.1 Was selection of participants into the study (or into the analysis) based on participant characteristics observed after the start of intervention?	N	
	2.2 If Y/PY to 2.1: Were the post-intervention variables that influenced selection likely to be associated with intervention?	NA	
	2.3 If Y/PY to 2.2: Were the post-intervention variables that influenced selection likely to be influenced by the outcome or a cause of the outcome?	NA	
	2.4 If N/PN to 2.1: Do start of follow-up and start of intervention coincide for most participants?	PY	
	2.5. If Y/PY to 2.2 and 2.3, or N/PN to 2.4: Were adjustment techniques used that are likely to correct for the presence of selection biases?	NA	
	**Risk of bias judgement**	**Low**	
**Bias in classification of interventions**	3.1 Were intervention groups clearly defined?	Y	
	3.2 Was the information used to define intervention groups recorded at the start of the intervention?	Y	
	3.3 Could classification of intervention status have been affected by knowledge of the outcome or risk of the outcome?	N	

	**Risk of bias judgement**	**Low**	
**Bias due to deviations from intended interventions**	**If your aim for this study is to assess the effect of assignment to intervention, answer questions 4.1 and 4.2**		
	4.1. Were there deviations from the intended intervention beyond what would be expected in usual practice?	Y	Adjuvant chemotherapy were significantly associated with disease-free and overall survival (data not shown).
	4.2. If Y/PY to 4.1: Were these deviations from intended intervention unbalanced between groups and likely to have affected the outcome?	Y	
	**If your aim for this study is to assess the effect of starting and adhering to intervention, answer questions 4.3 to 4.6**		
	4.3. Were important co-interventions balanced across intervention groups?	NA	
	4.4. Was the intervention implemented successfully for most participants?	NA	
	4.5. Did study participants adhere to the assigned intervention regimen?	NA	
	4.6. If N/PN to 4.3, 4.4 or 4.5: Was an appropriate analysis used to estimate the effect of starting and adhering to the intervention?	NA	
	**Risk of bias judgement**	**Serious**	
**Bias due to missing data**	5.1 Were outcome data available for all, or nearly all, participants?	Y	
	5.2 Were participants excluded due to missing data on intervention status?	PN	
	5.3 Were participants excluded due to missing data on other variables needed for the analysis?	PN	
	5.4 If PN/N to 5.1, or Y/PY to 5.2 or 5.3: Are the proportion of participants and reasons for missing data similar across interventions?	NA	
	5.5 If PN/N to 5.1, or Y/PY to 5.2 or 5.3: Is there evidence that results were robust to the presence of missing data?	NA	
	**Risk of bias judgement**	**Low**	
**Bias in measurement of the outcome**	6.1 Could the outcome measure have been influenced by knowledge of the intervention received?	N	The overall survival time was defined as the time, in months, from the date of surgery to the date of death, date of last follow-up, or censored date
	6.2 Were outcome assessors aware of the intervention received by study participants?	PY	
	6.3 Were the methods of outcome assessment comparable across intervention groups?	Y	
	6.4 Were any systematic errors in measurement of the outcome related to intervention received?	PN	
	**Risk of bias judgement**	**Low**	
**Bias in selection of the reported result**	Is the reported effect estimate likely to be selected, on the basis of the results, from…		
	7.1. ... multiple outcome measurements within the outcome domain?	PN	
	7.2 ... multiple analyses of the intervention-outcome relationship?	PN	
	7.3 ... different subgroups?	PN	
	**Risk of bias judgement**	**Low**	
**Overall bias**	**Risk of bias judgement**	**Serious**	

**Unique ID**	Park 2016	**Ref or Label**	Int J Gynaecol Obstet 2016;134(1):49-52.	**Design**	Individually randomized
**Participants**	Patients with epithelial ovarian carcinoma	**Experimental**	FSS	**Comparator**	RS
**Aim**	assignment to intervention (the 'intention-to-treat' effect)	**Outcome**	Recurrence rate	**Result**	Recurrent disease was noted in 5 (23%) patients in the fertility-sparing surgery group and 5 (20%) patients in the radical surgery group (P =0.820)

**Confounding domains listed in the review protocol**	**Measured variable(s)**	**Is there evidence that controlling for this variable was unnecessary?**	**Is the confounding domain measured validly and reliably by this variable?**	
Age	NA	No	Yes	
Race	NA	No	Yes	
Stage	Pathology report	No	Yes	
Grade	Pathology report	No	Yes	
Histology	Pathology report	No	Yes	
Tomor size	Clinical data	No	Yes	
Elevated serum CA125	Clinical data	No	Yes	
Intraoperative rupture	Clinical data	No	Yes	
Co-morbidities	Clinical data	No	Yes	

**Additional confounding domains**	**Measured variable(s)**	**Is there evidence that controlling for this variable was unnecessary?**	**Is the confounding domain measured validly and reliably by this variable?**	**OPTIONAL: Is failure to adjust for this variable (alone) expected to favour the experimental intervention or the comparator?**
NA	NA	NA	NA	NA

**Co-interventions listed in the review protocol**	**Is there evidence that controlling for this co-intervention was unnecessary (e.g. because it was not administered)?**	**Is presence of this co-intervention likely to favour outcomes in the experimental intervention or the comparator**
Chemotherapy	No	No information

**Additional co-interventions**	**Is there evidence that controlling for this co-intervention was unnecessary (e.g. because it was not administered)?**	**Is presence of this co-intervention likely to favour outcomes in the experimental intervention or the comparator**
NA	NA	NA

**Domain**	**Signalling questions**	**Response options**	**Description**
**Bias due to confounding**	1.1 Is there potential for confounding of the effect of intervention in this study?	Y	
	1.2 If Y/PY to 1.1: Was the analysis based on splitting participants' follow up time according to intervention received?	N	
	1.3 If Y/PY to 1.2: Were intervention discontinuations or switches likely to be related to factors that are prognostic for the outcome?	NA	
	**Questions relating to baseline confounding only**		
	1.4 If N/PN to 1.2 or 1.3: Did the authors use an appropriate analysis method that controlled for all the important confounding domains?	Y	Patients who underwent fertility-sparing surgery were significantly younger than were those who underwent radical surgery (P =0.001)(Table 1). More patients in the radical surgery group had a history of abdominal surgery (P = 0.010) and a parity of at least one (P b 0.001), but no other significant
	1.5 If Y/PY to 1.4: Were confounding domains that were controlled for measured validly and reliably by the variables available in this study?	Y	

	1.6. If N/PN to 1.2 or 1.3: Did the authors control for any post-intervention variables that could have been affected by the intervention?	Y	
	**Questions relating to baseline and time-varying confounding**		
	1.7. If Y/PY to 1.3: Did the authors use an appropriate analysis method that controlled for all the important confounding domains and for time-varying confounding?	NA	
	1.8. If Y/PY to 1.7: Were confounding domains that were controlled for measured validly and reliably by the variables available in this study?	NA	
	**Risk of bias judgement**	**Moderate**	
**Bias in selection of participants into the study**	2.1 Was selection of participants into the study (or into the analysis) based on participant characteristics observed after the start of intervention?	N	
	2.2 If Y/PY to 2.1: Were the post-intervention variables that influenced selection likely to be associated with intervention?	NA	
	2.3 If Y/PY to 2.2: Were the post-intervention variables that influenced selection likely to be influenced by the outcome or a cause of the outcome?	NA	
	2.4 If N/PN to 2.1: Do start of follow-up and start of intervention coincide for most participants?	PY	
	2.5. If Y/PY to 2.2 and 2.3, or N/PN to 2.4: Were adjustment techniques used that are likely to correct for the presence of selection biases?	NA	
	**Risk of bias judgement**	**Low**	
**Bias in classification of interventions**	3.1 Were intervention groups clearly defined?	Y	
	3.2 Was the information used to define intervention groups recorded at the start of the intervention?	Y	
	3.3 Could classification of intervention status have been affected by knowledge of the outcome or risk of the outcome?	N	
	**Risk of bias judgement**	**Low**	
**Bias due to deviations from intended interventions**	**If your aim for this study is to assess the effect of assignment to intervention, answer questions 4.1 and 4.2**		
	4.1. Were there deviations from the intended intervention beyond what would be expected in usual practice?	Y	Adjuvant chemotherapy were significantly associated with disease-free and overall survival (data not shown).
	4.2. If Y/PY to 4.1: Were these deviations from intended intervention unbalanced between groups and likely to have affected the outcome?	Y	
	**If your aim for this study is to assess the effect of starting and adhering to intervention, answer questions 4.3 to 4.6**		
	4.3. Were important co-interventions balanced across intervention groups?	NA	
	4.4. Was the intervention implemented successfully for most participants?	NA	
	4.5. Did study participants adhere to the assigned intervention regimen?	NA	
	4.6. If N/PN to 4.3, 4.4 or 4.5: Was an appropriate analysis used to estimate the effect of starting and adhering to the intervention?	NA	
	**Risk of bias judgement**	**Serious**	
**Bias due to missing data**	5.1 Were outcome data available for all, or nearly all, participants?	Y	
	5.2 Were participants excluded due to missing data on intervention status?	PN	
	5.3 Were participants excluded due to missing data on other variables needed for the analysis?	PN	
	5.4 If PN/N to 5.1, or Y/PY to 5.2 or 5.3: Are the proportion of participants and reasons for missing data similar across interventions?	NA	
	5.5 If PN/N to 5.1, or Y/PY to 5.2 or 5.3: Is there evidence that results were robust to the presence of missing data?	NA	
	**Risk of bias judgement**	**Low**	
**Bias in measurement of the outcome**	6.1 Could the outcome measure have been influenced by knowledge of the intervention received?	N	
	6.2 Were outcome assessors aware of the intervention received by study participants?	PY	
	6.3 Were the methods of outcome assessment comparable across intervention groups?	Y	
	6.4 Were any systematic errors in measurement of the outcome related to intervention received?	PN	
	**Risk of bias judgement**	**Low**	
**Bias in selection of the reported result**	Is the reported effect estimate likely to be selected, on the basis of the results, from…		
	7.1. ... multiple outcome measurements within the outcome domain?	PN	
	7.2 ... multiple analyses of the intervention-outcome relationship?	PN	
	7.3 ... different subgroups?	PN	
	**Risk of bias judgement**	**Low**	
**Overall bias**	**Risk of bias judgement**	**Serious**	
