# Supplementary material for: Oncological outcomes of fertility-sparing surgery versus radical surgery in stage - epithelial ovarian cancer: a systematic review and meta-analysis
Source: World J Surg Oncol. 2024 Jun 25;22:170. doi: 10.1186/s12957-024-03440-3 (PMC11201297; doi:10.1186/s12957-024-03440-3)
Supplement: Supplementary file 2 — Supplementary Material 2 [file 12957_2024_3440_MOESM2_ESM.pdf]

A

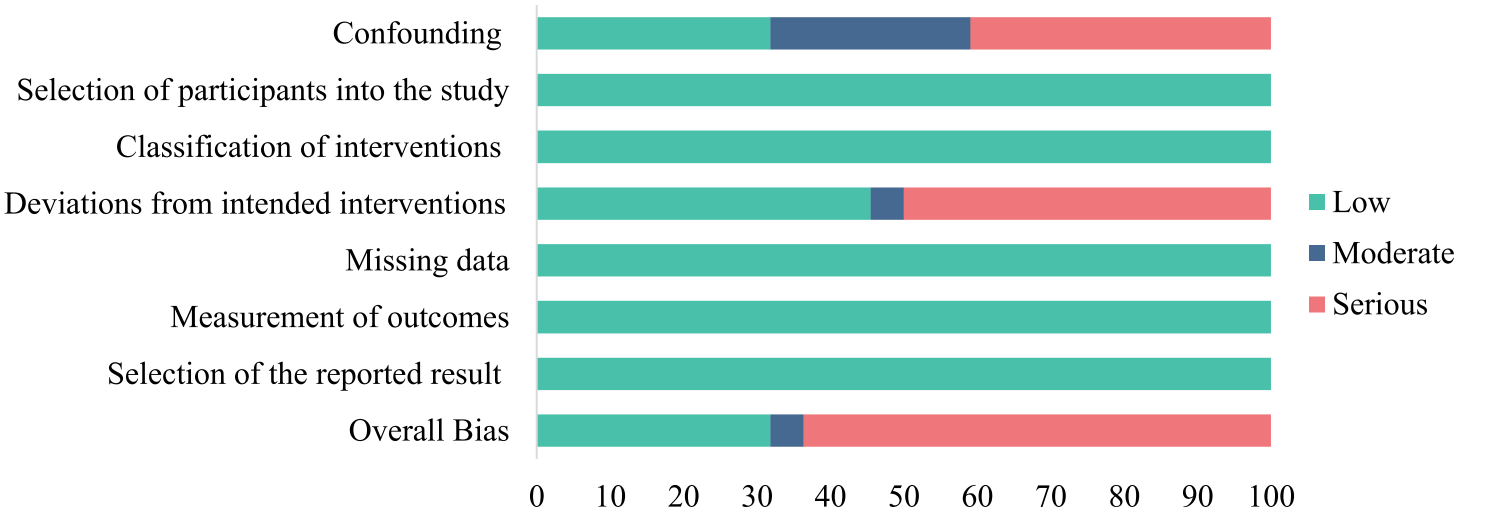

B

Unique ID	Outcome	A	B	C	D	E	F	G	H
Chen 2020	DFS	Low	Low	Low	Low	Low	Low	Low	Low
Chen 2020	Recurrence rate	Low	Low	Low	Low	Low	Low	Low	Low
Chen 2021	CSS	Serious	Low	Low	Low	Low	Low	Low	Serious
Chen 2021	OS	Serious	Low	Low	Low	Low	Low	Low	Serious
Colombo 1994	Recurrence rate	Serious	Low	Low	Serious	Low	Low	Low	Serious
Ditto 2014	Recurrence rate	Low	Low	Low	Low	Low	Low	Low	Low
Ditto 2014	DFS	Low	Low	Low	Low	Low	Low	Low	Low
Hedback 2018	DFS	Moderate	Low	Low	Moderate	Low	Low	Low	Moderate
Jiang 2017	DFS	Serious	Low	Low	Serious	Low	Low	Low	Serious
Jiang 2017	TSS	Serious	Low	Low	Serious	Low	Low	Low	Serious
Jiang 2017	Recurrence rate	Serious	Low	Low	Serious	Low	Low	Low	Serious
Jobo 2000	OS	Serious	Low	Low	Serious	Low	Low	Low	Serious
Johansen 2020	DFS	Serious	Low	Low	Serious	Low	Low	Low	Serious
Johansen 2020	OS	Serious	Low	Low	Serious	Low	Low	Low	Serious
Li 2023	OS	Moderate	Low	Low	Serious	Low	Low	Low	Serious
Lin 2022	DFS	Low	Low	Low	Low	Low	Low	Low	Low
Lin 2022	OS	Low	Low	Low	Low	Low	Low	Low	Low
Lin 2022	Recurrence rate	Low	Low	Low	Low	Low	Low	Low	Low
Nasioudis 2022	OS	Moderate	Low	Low	Serious	Low	Low	Low	Serious
Park 2016	DFS	Moderate	Low	Low	Serious	Low	Low	Low	Serious
Park 2016	OS	Moderate	Low	Low	Serious	Low	Low	Low	Serious
Park 2016	Recurrence rate	Moderate	Low	Low	Serious	Low	Low	Low	Serious

Low
Moderate
Serious

A Confounding
B Selection of participants into the study
C Classification of interventions
D Deviations from intended interventions
E Missing data
F Measurement of outcomes
G Selection of the reported result
H Overall Bias
