# Supplementary figures and images for: Oncological outcomes of fertility-sparing surgery versus radical surgery in stage - epithelial ovarian cancer: a systematic review and meta-analysis
Source: World J Surg Oncol. 2024 Jun 25;22:170. doi: 10.1186/s12957-024-03440-3 (PMC11201297; doi:10.1186/s12957-024-03440-3)

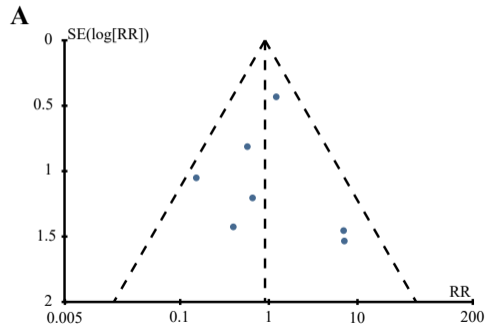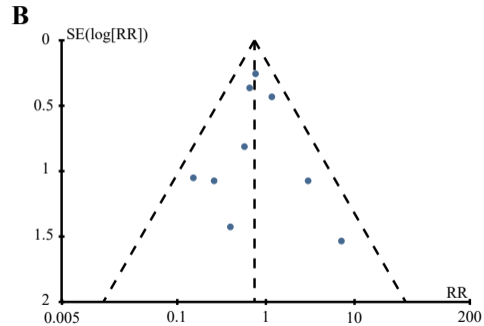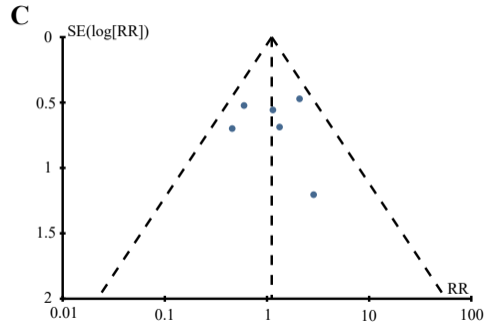

Supplement: Supplementary file 3 — Supplementary Material 3 [file 12957_2024_3440_MOESM3_ESM.pdf]
