# Supplementary material for: Oncological outcomes of fertility-sparing surgery versus radical surgery in stage - epithelial ovarian cancer: a systematic review and meta-analysis
Source: World J Surg Oncol. 2024 Jun 25;22:170. doi: 10.1186/s12957-024-03440-3 (PMC11201297; doi:10.1186/s12957-024-03440-3)
Supplement: Supplementary file 4 — Supplementary Material 4 [file 12957_2024_3440_MOESM4_ESM.pdf]

Search strategy in PUBMED

#1	((((((((((((((((((((((((((((((((((Carcinoma, Ovarian Epithelial[MeSH Terms]) OR (Epithelial Carcinoma, Ovarian[Title/Abstract])) OR (Ovarian Epithelial Carcinomas[Title/Abstract])) OR (Epithelial Ovarian Cancer[Title/Abstract])) OR (Ovarian Epithelial Cancer[Title/Abstract])) OR (Cancer, Ovarian Epithelial[Title/Abstract])) OR (Epithelial Cancer, Ovarian[Title/Abstract])) OR (Ovarian Epithelial Cancers[Title/Abstract])) OR (Ovarian Cancer, Epithelial[Title/Abstract])) OR (Cancer, Epithelial Ovarian[Title/Abstract])) OR (Epithelial Ovarian Cancers[Title/Abstract])) OR (Ovarian Epithelial Carcinoma[Title/Abstract])) OR (Epithelial Ovarian Carcinoma[Title/Abstract])) OR (Carcinoma, Epithelial Ovarian[Title/Abstract])) OR (Epithelial Ovarian Carcinomas[Title/Abstract])) OR (Ovarian Carcinoma, Epithelial[Title/Abstract])) OR (advanced ovary carcinoma[Title/Abstract])) OR (carcinoma in ovary[Title/Abstract])) OR (carcinoma in the ovary[Title/Abstract])) OR (carcinoma of ovaries[Title/Abstract])) OR (carcinoma of ovary[Title/Abstract])) OR (carcinoma of the ovaries[Title/Abstract])) OR (carcinoma of the ovary[Title/Abstract])) OR (carcinoma ovarii[Title/Abstract])) OR (carcinoma, ovarian epithelial[Title/Abstract])) OR (carcinomatous ovarian[Title/Abstract])) OR (epithelial ovary cancer[Title/Abstract])) OR (ovarian carcinoma[Title/Abstract])) OR (ovarian carcinomata[Title/Abstract])) OR (ovarian carcinomatosis[Title/Abstract])) OR (ovarian epithelial carcinogenesis[Title/Abstract])) OR (ovarium carcinoma[Title/Abstract])) OR (ovary epithelial cancer[Title/Abstract])) OR (primary ovary carcinoma[Title/Abstract])) OR (ovary carcinoma[Title/Abstract])
#2	(((((((((((Fertility Preservation[MeSH Terms]) OR (Fertility Preservations[Title/Abstract])) OR (Preservation, Fertility[Title/Abstract])) OR (Fertility Sparing[Title/Abstract])) OR (conservative surgery[Title/Abstract])) OR (conservative treatment[Title/Abstract])) OR (conservative issue[Title/Abstract])) OR (conservative management[Title/Abstract])) OR (conservative operation[Title/Abstract])) OR (radical operation[Title/Abstract])) OR (radical surgery[Title/Abstract])) OR (radical treatment[Title/Abstract])
#3	#1 AND #2

Search strategy in WEB OF SCIENCE

#1	(((((((((((TS=(Fertility Preservation)) OR TS=(Fertility Preservations)) OR TS=(Preservation, Fertility)) OR TS=(Fertility Sparing)) OR TS=(conservative surgery)) OR TS=(conservative treatment)) OR TS=(conservative issue)) OR TS=(conservative management)) OR TS=(conservative operation)) OR TS=(radical operation)) OR TS=(radical surgery)) OR TS=(radical treatment) and Preprint Citation Index (Exclude – Database)
#2	((((((((((((((((((((((((((((((((((TS=(Carcinoma, Ovarian Epithelial)) OR TS=(Epithelial Carcinoma, Ovarian)) OR TS=(Ovarian Epithelial Carcinomas)) OR TS=(Epithelial Ovarian Cancer)) OR TS=(Ovarian Epithelial Cancer)) OR TS=(Cancer, Ovarian

	Epithelial)) OR TS=(Epithelial Cancer, Ovarian)) OR TS=(Ovarian Epithelial Cancers)) OR TS=(Ovarian Cancer, Epithelial)) OR TS=(Cancer, Epithelial Ovarian)) OR TS=(Epithelial Ovarian Cancers)) OR TS=(Ovarian Epithelial Carcinoma)) OR TS=(Epithelial Ovarian Carcinoma)) OR TS=(Carcinoma, Epithelial Ovarian)) OR TS=(Epithelial Ovarian Carcinomas)) OR TS=(Ovarian Carcinoma, Epithelial)) OR TS=(advanced ovary carcinoma)) OR TS=(carcinoma in ovary)) OR TS=(carcinoma in the ovary)) OR TS=(carcinoma of ovaries)) OR TS=(carcinoma of ovary)) OR TS=(carcinoma of the ovaries)) OR TS=(carcinoma of the ovary)) OR TS=(carcinoma ovarii)) OR TS=(carcinoma, ovarian epithelial)) OR TS=(carcinomatous ovarian)) OR TS=(epithelial ovary cancer)) OR TS=(ovarian carcinoma)) OR TS=(ovarian carcinomata)) OR TS=(ovarian carcinomatosis)) OR TS=(ovarian epithelial carcinogenesis)) OR TS=(ovarium carcinoma)) OR TS=(ovary epithelial cancer)) OR TS=(primary ovary carcinoma)) OR TS=(ovary carcinoma) and Preprint Citation Index (Exclude – Database)
#3	#1 AND #2

Search strategy in EMBASE

#1	('carcinoma,'/exp OR carcinoma,) AND ovarian AND epithelial
#2	epithelial AND carcinoma, AND ovarian
#3	ovarian AND epithelial AND carcinomas
#4	epithelial AND ovarian AND cancer
#5	ovarian AND epithelial AND cancer
#6	cancer, AND ovarian AND epithelial
#7	epithelial AND cancer, AND ovarian
#8	ovarian AND epithelial AND cancers
#9	ovarian AND cancer, AND epithelial
#10	cancer, AND epithelial AND ovarian
#11	epithelial AND ovarian AND cancers
#12	ovarian AND epithelial AND carcinoma
#13	epithelial AND ovarian AND carcinoma
#14	carcinoma, AND epithelial AND ovarian
#15	epithelial AND ovarian AND carcinomas
#16	ovarian AND carcinoma, AND epithelial
#17	advanced AND ovary AND carcinoma
#18	carcinoma AND in AND ovary
#19	carcinoma AND in AND the AND ovary
#20	carcinoma AND of AND ovaries
#21	carcinoma AND of AND ovary
#22	carcinoma AND of AND the AND ovaries
#23	carcinoma AND of AND the AND ovary
#24	carcinoma AND ovarii
#25	carcinoma, AND ovarian AND epithelial

#26	carcinomatous AND ovarian
#27	epithelial AND ovary AND cancer
#28	ovarian AND carcinoma
#29	ovarian AND carcinomata
#30	ovarian AND carcinomatosis
#31	ovarian AND epithelial AND carcinogenesis
#32	ovarium AND carcinoma
#33	ovary AND epithelial AND cancer
#34	primary AND ovary AND carcinoma
#35	ovary AND carcinoma
#36	#1 OR #2 OR #3 OR #4 OR #5 OR #6 OR #7 OR #8 OR #9 OR #10 OR #11 OR #12 OR #13 OR #14 OR #15 OR #16 OR #17 OR #18 OR #19 OR #20 OR #21 OR #22 OR #23 OR #24 OR #25 OR #26 OR #27 OR #28 OR #29 OR #30 OR #31 OR #32 OR #33 OR #34 OR #35
#37	fertility AND preservation
#38	fertility AND preservations
#39	preservation, AND fertility
#40	fertility AND sparing
#41	conservative AND surgery
#42	conservative AND treatment
#43	conservative AND issue
#44	conservative AND management
#45	conservative AND operation
#46	radical AND operation
#47	radical AND surgery
#48	radical AND treatment
#49	#37 OR #38 OR #39 OR #40 OR #41 OR #42 OR #43 OR #44 OR #45 OR #46 OR #47 OR #48
#50	#36 AND #49
