# Supplementary material for: Oncological outcomes of fertility-sparing surgery versus radical surgery in stage - epithelial ovarian cancer: a systematic review and meta-analysis
Source: World J Surg Oncol. 2024 Jun 25;22:170. doi: 10.1186/s12957-024-03440-3 (PMC11201297; doi:10.1186/s12957-024-03440-3)
Supplement: Supplementary file 5 — Supplementary Material 5 [file 12957_2024_3440_MOESM5_ESM.docx]

**Table. S1** Preferred Reporting Items for Systematic Review and Meta-analyses (PRISMA) checklist.

**Table. S2** Preferred Reporting Items for Systematic Review and Meta-analyses (PRISMA) abstract checklist.

**Table. S3** Search strategy.

**Table. S4** Detailed quality assessment using the Cochrane Risk of Bias in Non-randomized Studies of Interventions (ROBINS-I) tool for each study and outcome.

**Figure. S5** Final results of quality assessment using the Cochrane Risk of Bias in Non-randomized Studies of Interventions (ROBINS-I) tool for **(A)** each study and **(B)** each outcome. OS, overall survival; DFS, disease-free survival; TSS, tumor-specific survival; CSS, cancer-specific survival. The color of each cell indicates the risk of bias for each study and outcome. Overall, the reporting of studies was good, the selection of participants, classification of interventions, management of missing data, measurement of outcomes, and selection of reported results were generally clearly stated. Due to incomplete consideration of confounding factors and inappropriate deviations from the intended interventions, one study was evaluated as having a moderate risk of bias, and seven studies were evaluated as having a serious risk of bias.

**Figure. S6** Funnel plots of studies comparing disease-free survival, overall survival, and recurrence rate of fertility-sparing surgery with radical surgery. **(A)** There is no evidence of publication bias in the meta-analyses of disease-free survival. **(B)** There is no evidence of publication bias in the meta-analyses for overall survival. **(C)** There is no evidence of publication bias in the meta-analysis of recurrence rate.
